# Integration mapping of cardiac fibroblast single-cell transcriptomes elucidates cellular principles of fibrosis in diverse pathologies

**DOI:** 10.1126/sciadv.adk8501

**Published:** 2024-06-21

**Authors:** Ralph Patrick, Vaibhao Janbandhu, Vikram Tallapragada, Shannon S. M. Tan, Emily E. McKinna, Osvaldo Contreras, Shila Ghazanfar, David T. Humphreys, Nicholas J. Murray, Yen T. H. Tran, Robert D. Hume, James J. H. Chong, Richard P. Harvey

**Affiliations:** ^1^ Victor Chang Cardiac Research Institute, Darlinghurst, NSW 2010, Australia.; ^2^ School of Clinical Medicine, UNSW Sydney, Kensington, NSW 2052, Australia.; ^3^ Institute for Molecular Bioscience, The University of Queensland, St. Lucia, QLD 4072, Australia.; ^4^ Westmead Institute for Medical Research, The University of Sydney, Westmead, NSW 2145, Australia.; ^5^ School of Mathematics and Statistics, The University of Sydney, Camperdown, NSW 2006, Australia.; ^6^ Charles Perkins Centre, The University of Sydney, Camperdown, NSW 2006, Australia.; ^7^ Sydney Precision Data Science Centre, The University of Sydney, Camperdown, NSW 2006, Australia.; ^8^ School of Medical Science, The University of Sydney, Camperdown, NSW 2006, Australia.; ^9^ Centre for Heart Failure and Diseases of the Aorta, The Baird Institute, Sydney, NSW 2042, Australia.; ^10^ Department of Cardiology, Westmead Hospital, Westmead, NSW 2145, Australia.; ^11^ School of Biotechnology and Biomolecular Science, UNSW Sydney, Kensington, NSW 2052, Australia.

## Abstract

Single-cell technology has allowed researchers to probe tissue complexity and dynamics at unprecedented depth in health and disease. However, the generation of high-dimensionality single-cell atlases and virtual three-dimensional tissues requires integrated reference maps that harmonize disparate experimental designs, analytical pipelines, and taxonomies. Here, we present a comprehensive single-cell transcriptome integration map of cardiac fibrosis, which underpins pathophysiology in most cardiovascular diseases. Our findings reveal similarity between cardiac fibroblast (CF) identities and dynamics in ischemic versus pressure overload models of cardiomyopathy. We also describe timelines for commitment of activated CFs to proliferation and myofibrogenesis, profibrotic and antifibrotic polarization of myofibroblasts and matrifibrocytes, and CF conservation across mouse and human healthy and diseased hearts. These insights have the potential to inform knowledge-based therapies.

## Teaser

We reveal cardiac fibroblast identity and flux in health and disease by integration of diverse single-cell transcriptome datasets.

## INTRODUCTION

The single-cell revolution has prompted reevaluation of cellular and epigenetic mechanisms underpinning tissue development, homeostasis, aging, disease, and regeneration, agnostic to previously held notions of cell origin and fate (*1*). One of the key goals of this field is to develop single-cell, high-dimensionality atlases for defining cell phenotypes that will form a new framework for understanding animal biology, with the promise of major clinical impacts (*2*, *3*).

Cardiovascular (CV) disease represents the lead cause of death and disability worldwide. A consistent feature of CV disease is cardiac fibrosis, which leads to the excessive deposition of disorganized extracellular matrix (ECM) due to unrestrained or inappropriate activation of reparative pathways (*4*). The major drivers of cardiac fibrosis are activated fibroblasts and contractile myofibroblasts (MYOs). Cardiac fibroblasts (CFs) are highly plastic cells that act as sentinels and maintain tissue integrity through their roles as paracrine signaling hubs, lineage progenitors, and electrical and mechanical transducers (*5*–*10*). However, in disease, they morph into profibrotic cells acting as inflammatory and vascular modulators and ECM factories (*4*, *11*–*13*). Although fibrosis may initially be protective and reparative (*14*, *15*), an inability to resolve injurious stimuli leads to a self-perpetuating and amplifying fibrotic cascade, with heart failure as the end stage of progression (*16*). The notion of pathological fibrosis as dysregulated tissue repair presents a duality that has implications for how we understand and treat fibrosis, and the notable failure of antifibrotic drug discovery efforts to date has highlighted the inadequacy of our current models of fibroblast-to-MYO transition (*12*, *17*).

Single-cell RNA sequencing (scRNA-seq) has revealed unexpected heterogeneity of cardiac cell populations (*9*, *18*–*24*). However, the origins and three-dimensional (3D) spatial dynamics of identified populations remain poorly understood. One promise of this field is that proregenerative and pathological fibrosis become distinguishable at the cellular and molecular level and could be targeted selectively. A key hurdle to developing meaningful high-dimensionality single-cell tissue atlases and reconstructed 3D tissue spaces is that individual studies vary with respect to species, disease model, experimental design, time points, depth of cell and sequence coverage, bioinformatic pipelines, and cell taxonomies. A high priority, therefore, is to develop integration reference maps that overcome these hurdles and drive deeper interrogation of biology (*25*, *26*).

Here, we report a comprehensive integration analysis of fibroblast states in cardiac ventricles in homeostasis and after ischemic and nonischemic injury in mice and humans. We confirm CF diversity and reveal conservation of resting, activated, and differentiated states across mammalian species. We uncover insights that consolidate our understanding of cardiac fibrosis progression and resolution in diverse CV disease models, providing a stronger framework for knowledge-based therapeutics.

We created CardiacFibroAtlas, an enhanced ShinyCell web application (*27*) as a public resource, enabling visualization and analysis of gene expression in both myocardial infarction (MI) and cross-disease integrated datasets (available at http://CardiacFibroAtlas.victorchang.edu.au).

## RESULTS

**Fig. 1.**
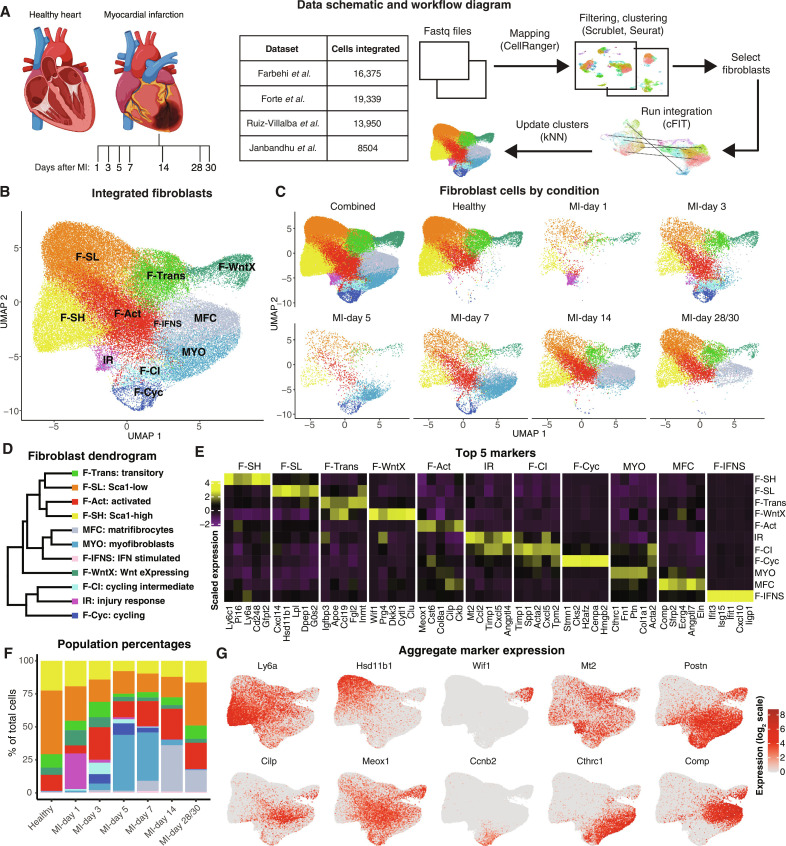
MI integration of CFs. (**A**) Schematic and table of datasets used and workflow for integrative analysis. Created with BioRender.com. (**B**) UMAP plot showing an aggregate of CFs across conditions. (**C**) UMAP plot showing CFs according to condition. (**D**) Dendrogram of CF subtypes determined by average batch-corrected expression in populations. (**E**) Heatmap of top 5 marker genes per CF population [MAST testing; *P*_adj_ < 1 × 10^−05^; log_2_(fold change) > 0.5]. (**F**) Percentage of cells in each population according to experimental condition. (**G**) Expression of select genes in different CF populations as visualized on UMAP coordinates.

We first built a comprehensive single-cell transcriptomics map incorporating four foundation studies covering seven time points of MI and associated uninjured/sham-operated controls (Fig. 1A). All studies were from dissected ventricles. Three studies used genetic lineage tagging to enrich for CFs at single or multiple time points (*8*, *9*, *28*), and in an additional study conducted on total cardiac interstitial (noncardiomyocyte) cells at days 1, 3, 5, 7, 14, and 28 after MI, CFs were selected in silico (Fig. 1A) (*10*). These datasets were processed uniformly incorporating initial clustering of CFs, with cell types manually annotated according to previously described CF subtypes defined in pioneering studies by Farbehi *et al.* (*9*) and Forte *et al.* (*10*) based on marker gene expression (Materials and Methods; fig. S1, A to F). Integration was performed using common factor integration and transfer learning (cFIT) (*25*), with Uniform Manifold Approximation and Projection (UMAP) visualization performed on the integrated low-dimensional space returned by cFIT. We compared results from cFIT to other integration methods, including Seurat (*26*), Harmony (*29*), and Robust Integration of Single-Cell RNA-seq data (RISC) (*30*), and found that cFIT was best able to remove batch effects while retaining expected biological heterogeneity (fig. S2, A to H). For example, when overlapping cell types among experimental conditions, the expected segregation of MYOs and matrifibrocytes (MFCs) (see below) was strongest with cFIT as indicated by a nearest neighbor analysis (fig. S2E). Last, we used a *k*-nearest neighbors (kNN) approach to refine original cluster identifiers (IDs) based on the majority 25 nearest cells (Fig. 1, A and B).

Thus far, CF nomenclature is neither systematic nor intuitive. To avoid further complexity, we have used nomenclatures aligned with foundation studies (*9*, *10*), however, prioritizing our own labels for clarification of population complexity. Table 1 is a resource that shows study details and cross-references study-specific population taxonomies and top up-regulated markers.

**Table 1.** Cross-comparison of major CF subpopulations in healthy and diseased mouse hearts. Fibroblast abbreviations—Farbehi *et al.* study (*9*): F-SH, Fibroblast-Sca1^high^; F-SL, Fibroblast-Sca1^low^; F-Trans, Fibroblast-transitory; F-IFNS, Fibroblast-interferon stimulated; F-WntX, Fibroblast-Wnt expressing; F-Act, Fibroblast-activated; F-CI, Fibroblast-cycling intermediate; F-Cyc, Fibroblast cycling; MYO, myofibroblast. Fibroblast abbreviations—Forte *et al.* study (*10*): PLS, progenitor-like state fibroblast; HEpiD, homeostatic epicardial-derived fibroblasts; IFNr, interferon response; EndD, endocardial-derived fibroblasts; LR, late-resolution fibroblasts; IR, IR fibroblasts; ProlifMyofb, proliferating myofibroblasts; Myofb, myofibroblasts; MFC, matrifibrocytes. Other abbreviations: aCSC, activated cardiac stromal cells; AngII, AngII-induced heart failure model; CSC, cardiac stromal cells; ns, not specified; RCF, reparative CF.

Study	Farbehi *et al.* (*9*)	Forte *et al.* (*10*)	Janbandhu *et al.* (*8*)	Ruiz-Villalba *et al.* (*28*)	Hesse *et al.* (*62*)	McLellan *et al.* (*63*)	Alexanian *et al.* (*51*)	
**Model**	Sham + MI^*^	Uninjured + MI^†^	Uninjured + sham + MI^‡^	Uninjured + MI^§^	Sham + IRI	Untreated + Saline + AngII	Sham + TAC	
**CF enrichment**	*Pdgfra*-GFP^+^	In silico from nonmyocytes	*Pdgfra*-lineage^+^CD31^−^CD45^−−^	*Col1a1*-GFP^+^	In silico from CD31^−^CD45^−^ nonmyocytes	In silico from myocytes and nonmyocytes	In silico from nonmyocyte	
**After injury days**	3, 7	1, 3, 5, 7, 14, 28	3	7, 14, 30	5	14	62	
**Total cells integrated**	16,375	19,339	8504	13,950	26,828	14,046	9477	
**Male/female**	Male	Male and female	Male	ns	Male	Male and female	ns	
**Strain**	C57Bl/6J	C57Bl/6J	C57Bl/6J	C3H/C57B1^¶^	C57Bl/6J	C57Bl/6J	ns	
								**Top enriched markers** ^**^
**Populations**	F-SH	PLS^#^	F-SH	Cluster D^#^	CSC-4 and aCSC-4^#^	Fibroblast-6^#^	ns^#^	*Ly6c1;Pi16;Ly6a(Sca1);Cd248;Gfpt2*
	F-SL	HEpiD	F-SL	Cluster A/C	CSC-1 and CSC-2	ns	ns	*Cxcl14;Hsd11b1;Lpl;Dpep1;G0s2*
	F-Trans	ns	F-Trans	Cluster H	CSC-5	Fibroblast-2	ns	*Igfbp3;Apoe;Ccl19;Fgl2;Inmt*
	F-IFNS	IFNr	F-IFNS	Cluster I	CSC-9 and aCSC-8	Fibroblast-9	ns	*Ifit3;Isg15;Ifit1;Cxcl10;Ligp1*
	F-WntX	EndD	F-WntX	Cluster H	CSC-11 and aCSC-11	Fibroblast-Wif1	ns	*Wif1*;*Prg4;Dkk3*; *Cytl1;Clu*
	F-Act	LR	F-Act	Cluster F	CSC-3	Fibroblast-Clip	ns	*Meox1;Cst6;Col8a1;Cilp*;*Ckb*
	F-CI	ns	F-CI	ns	ns	ns	ns	*Timp1;Spp1;Acta2;Cxcl5;Tmp2*
	ns	IR	ns	ns	ns	ns	ns	*Mt2*;*Ccl2*;*Timp1;Cxcl5;Angptl4*
	F-Cyc	ProlifMyofb	F-Cyc	ns	aCSC-7 and aCSC-10	ns	ns	*Stmn1;Cks2;H2afz;Cenpa;Hmgb2*
	MYO	Myofb	ns	Cluster B (RCF)	aCSC-1, aCSC-2, and aCSC-3	Absent	ns	MYO: *Cthrc1;Fn1,Ptn;Col1a1;Acta2*
	MYO-1	ns	ns	ns		ns	ns	MYO-1: *Wisp2*;*Sfrp2*;*Sfrp1,Ccn5,Fbln1*
	MYO-2	ns	ns	ns		ns	ns	MYO-2: *Tgfb1*;*Thbs4;Crlf1,Col15a1*
	ns	MFC	ns	Cluster B (RCF) day 14/30	ns	Fibroblast-Thbs4	ns	*Comp*;*Sfrp2*;*Ecrg4*;*Angptl7*;*Eln*

*Uninjured and MI mice were on a heterozygous *Pdgfra^GFP^* background.

†Uninjured and MI mice were on a *TgWt1^IRES-EGFP-Cre^;RosaZsGreen^f/+^* background.

‡Uninjured, sham and MI mice were on conditional (*Pdgfra^merCRE-mer^*) heterozygous *Hif1a* knockout background.

§Uninjured and MI mice were on a *TgCol1a1^GFP^* background.

¶Transgene background strain not specified in the Ruiz-Villalba *et al.* study (*28*); original background cited as C3H/C57B1.

#CF sub-populations were inferred by comparison with reported marker gene lists where available.

**Top 5 enriched markers taken from integrated data (see Fig. 1F and table S1). See text for MYO-1 and MYO-2 markers.

### Definition of major CF subpopulations in healthy and MI hearts

Following integration, we defined 11 CF subpopulations consistent with previous reports (Fig. 1, B to D) (*9*, *10*). Subpopulations are summarized below.

#### F-SH and F-SL

Healthy hearts were characterized by five main CF subtypes (Fig. 1, B to F). The two largest populations—Fibroblast-*Sca1*^high^ (F-SH) and Fibrobast-*Sca1*^low^ (F-SL) expressed *Pdgfra* (*5*, *12*) and are distinguished on the basis of high and low expression of *Ly6a/Sca1* and other stem cell markers (*9*). We have previously shown that F-SH correlates to the PDGFRα^+^SCA1^+^ (S^+^P^+^) subfraction of adult CFs defined by flow cytometry and enriched for cardiac mesenchymal stem/stromal cell (MSC) colony-forming units (*5*). We anticipate that cardiac MSCs represent an immature reserve population for proliferation and generation of specialized CFs in homeostasis and after injury (*6*, *8*, *31*, *32*), akin to the formation of fibro-adipocyte-chondrocyte-osteocyte lineage derivatives from bona fide MSCs in other tissues (*33*–*38*). Cardiac S^+^P^+^ cells have also been referred to as cardiac fibro/adipogenic progenitors as they likely give rise to both fibrotic and adipose infiltrations in homeostasis and different disease states, as shown in mouse arrhythmogenic cardiomyopathy models (*6*, *39*). The other major resting population, F-SL (*Ly6a*/*Sca1*^low^), is marked by higher levels of *Cxcl14* and *Hsd11b1* (Fig. 1E and Table 1) and may arise from F-SH in dynamic equilibrium (*6*).

#### F-WntX and F-Trans

The most transcriptionally distinct CF population was Fibroblast-Wnt–expressing (F-WntX) (Fig. 1, B to F) (*9*), present in both healthy and MI hearts (Fig. 1, C and F) and defined by high expression of WNT pathway genes (*9*) including secreted WNT signaling pathway inhibitors WIF1, DKK3, and SFRP2 (Fig. 1, E and G). F-WntX expresses high levels of activation markers and lower levels of stem cell markers (Fig. 1G) (*9*, *10*), and its signature has been noted to be similar to specialized fibroblasts present within cardiac valves (*10*, *40*). Like valvular CFs, F-WntX may have an epicardial origin (*10*). Fibroblast-transitory (F-Trans), marked by up-regulation of *Fgl2* and *Igfbp3* (Table 1 and table S1), is also present in uninjured and MI hearts and appears to be an intermediary population between F-SL and F-WntX, although it is most related to F-SL (Fig. 1, B and D) (*9*).

#### IR

Integration analysis recovered injury response (IR) CFs (Fig. 1, C and F), initially found by Forte *et al.* (*10*) in MI hearts, peaking at MI-day 1 and persisting through MI-days 3 and 5 (representing ~60, ~19, and ~16% of total CFs at these respective times). In our integration data, IR was more narrowly defined being limited principally to MI-day 1 (Fig. 1F and fig. S2I), corresponding to the peak of neutrophil infiltration (*41*). IR expresses higher levels of metallothionein antioxidant genes, *Mt1* and *Mt2* (*10*), which can modulate injury-induced oxidative stress and fibrosis in the heart (*42*), as well as neutrophil and monocyte/macrophage chemoattractants and activators including CCL7, CCL2, CXCL5, and MIF (Fig. 1, E and G, and table S1) (*10*), suggesting that IR is a proinflammatory CF population, potentially related to those reported previously (*43*). Analysis of differential mRNA 3′ untranslated region (3′UTR) use with Sierra (*44*) revealed that IR at MI-day 1 shows global shortening of 3′UTRs (fig. S3A), a feature of dividing cells (*45*) including cycling CF (*44*), indicating that IR has engaged a preproliferative transcriptional state.

#### F-Act

Fibroblast-activated (F-Act) was found in moderate abundance in healthy hearts, expanding two- to threefold after MI, consistent with our previous report (Fig. 1, B to F) (*9*). F-Act overall shows increased expression of ECM and tissue development genes yet retains a transcriptome signature reflecting multilineage priming (a feature of stemness), as found in the quiescent F-SH population (*8*). F-Act is seen throughout the MI time course (Fig. 1, C and F) (*10*), well beyond the CF proliferative window (*12*), potentially due to continuous induction. A key feature of F-Act, increasingly so in injured hearts, is the graded expression of canonical CF activation markers *Postn*, *Cilp*, *Meox1*, *Col8a1*, and others across UMAP space antithetical to diminishing stem cell–related markers such as *Ly6a/Sca1* (Fig. 1, C, E, and G) (*9*). However, F-Act segments as a distinct state from MYOs, showing limited expression of *Acta2* [encoding α–smooth muscle actin (α-SMA)] and the MYO signature gene *Cthrc1* (*9*) and lower levels of ECM genes (e.g., *Col1a1* and *Fn1*).

#### F-CI and F-Cyc

A previously described early injury–dependent subpopulation was Fibroblast-cycling intermediate (F-CI) (Fig. 1, B to F), present predominantly at MI-day 3 and diminishing thereafter. The time course of F-CI resembles that of proliferating CFs [Fibroblasts-cycling (F-Cyc)], although they are proportionally more abundant than F-Cyc at the peak of proliferation at MI-day 3 (*46*), compared to later stages. F-CI cells show a close transcriptional relationship to F-Cyc (*9*) and also global shortening of mRNA 3′UTRs, as found for IR and F-Cyc (*44*). Compared to resting fibroblasts, F-CI and F-Cyc show strong up-regulation of a protein biosynthetic gene program (*9*), also a signature of cell cycle engagement (*44*). Previous cell trajectory and RNA velocity analyses suggest that F-CI represents preproliferative and postproliferative CFs (*8*, *9*).

#### MYO and MFC

We identified MYOs from MI-days 5–7 and MFCs from MI-day 7, consistent with previous reports (Fig. 1, C and F, and fig. S2I) (*9*, *10*). MYO is marked by the expression of *Postn*, *Acta2*, *Cthrc1*, *Tgfb1*, *Scx*, and other profibrotic genes and shows up-regulation of numerous collagen and ECM remodeling genes compared to other fibroblast states (Fig. 1, E and G, and table S1) (*9*). MFCs are a recently described CF population, which persist within MI scar in mouse, likely participating in its remodeling (*46*), although the function of MFC is unknown. Lineage tracing shows that MFC arise from MYOs during MI repair and they are unable to proliferate after an additional stimulus, with molecular profiling suggesting that they acquire a less activated, noncontractile (α-SMA^−^) state, however, emphasizing chondrogenic and osteogenic ECM gene signatures (*46*). Figure 1F indicates that MFCs first appear in small numbers at MI-day 7, increase substantially by MI-day 14, and persist at diminished levels out to MI-days 28/30, times at which MYOs have completely resolved. Overall, MFCs are distinguished by up-regulation of antifibrotic genes including *Comp* and *Sfrp2* (Fig. 1, E and G, and table S1).

#### F-IFNS

Our integration analysis also recovered the rare inflammatory CF population, Fibroblast-interferon stimulated (F-IFNS) (*9*, *18*). F-IFNS show a strong type I interferon–responsive signature. Interferon signatures have also been seen in endothelial cells and pericytes in a pressure overload model (*47*). The low abundance, interferon signature, and presence throughout the MI time course distinguish F-IFNS from IR (also inflammatory), which shows a sharp peak at MI-day 1 (Fig. 1, D and F).

### Trajectory analysis

**Fig. 2.**
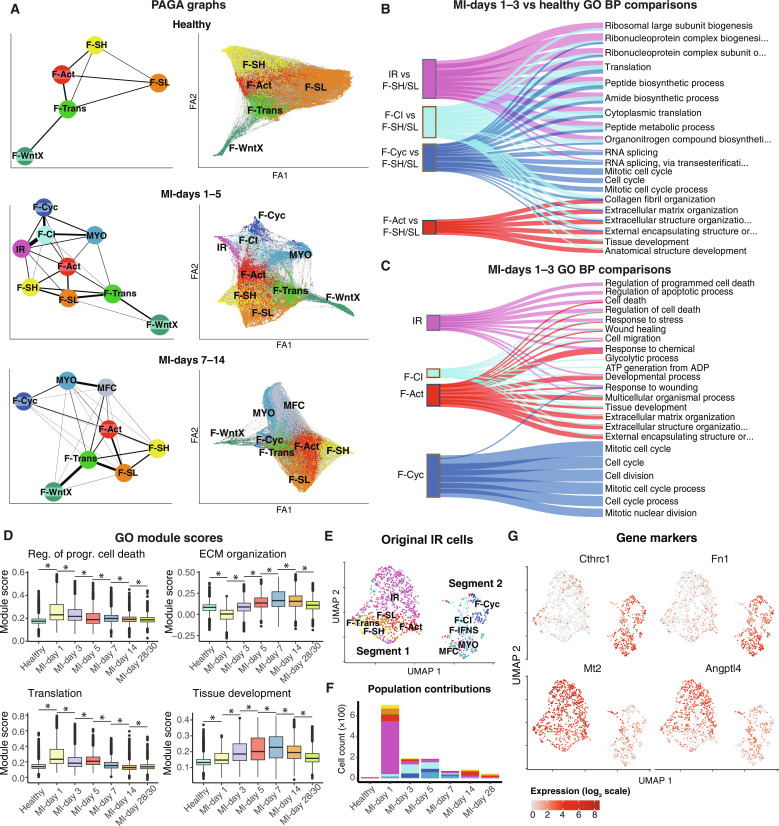
Trajectory analysis of the MI time course. (**A**) PAGA graphs of cells from healthy hearts, MI-days 1 to 5, or MI-days 7 to 14. Shown are (left) PAGA trajectories between cell types and (right) the force atlas (FA) layout of cells with top 10 nearest neighbor connections. (**B**) Sankey plot of top 6 GO BP terms among up-regulated genes [MAST testing; *P*_adj_ < 1 × 10^−05^; log_2_(fold change) > 0.5] per population at MI-days 1 and 3 in comparison to resting (F-SH and F-SL) fibroblasts from uninjured hearts. (**C**) Sankey plot of top 6 GO BP terms comparing each of the indicated activated populations to the remainder at MI-days 1 and 3. (**D**) Module scores for DEGs within select GO terms across the MI time points. * indicates a statistically significant difference (Bonferroni-adjusted *P* < 0.05) according to a two-sided Wilcoxon rank sum test. (**E**) UMAP of IR cells as identified by initial clustering of the Forte *et al.* data, with updated cell labels following the kNN analysis incorporating all MI datasets. (**F**) Population proportion breakdown of (E) according to time point. (**G**) Expression of indicated genes on the UMAP coordinates of the IR cells from the initial clustering.

We applied partition-based graph abstraction (PAGA) (*48*) to infer cell differentiation trajectories across our integrated MI time course. We performed analyses in healthy hearts as well as at early and late time points of MI as aggregates (MI-days 1 to 5 and 7 to 14) to help resolve transition points to MYO and MFC, respectively (Fig. 2A). Trajectories between different stages of CF activation, proliferation, and differentiation were nonlinear, as previously predicted (*9*). There were several notable features. For example, multiple direct interconnections existed between all major resting CFs in healthy hearts, apart from F-WntX. At MI-days 1 to 5, the strongest connection to proliferating CFs (F-Cyc) was from F-CI, which was also strongly connected to IR (present earlier at MI-day 1). Collectively, this supports F-CI as a key stepping stone from IR and F-Cyc and other states at early phases. Although the strongest connection to MYO was from F-CI, F-Cyc could also directly connect, suggesting that MYO can arise via nonproliferative and proliferative routes, also evident at MI-days 7 to 14. The strongest connection to MFC came from MYO, as expected (Fig. 2A). We observed similar trajectory trends based on analysis with Monocle 3 (fig. S3B) (*49*).

### Differential gene expression analysis

To further understand the differences between the four main early activated CF subpopulations (F-Act, IR, F-CI, and F-Cyc), we calculated differentially expressed genes (DEGs) [Model-based Analysis of Single-cell Transcriptomics (MAST) testing (*50*); *P*_adj_ < 1 × 10^−05^; log_2_(fold change) > 0.5] between these and the main resting populations from healthy hearts (F-SH and F-SL combined) and calculated Gene Ontology (GO) biological process (BP) terms (Fig. 2B). Early injury–dependent populations IR, F-CI, and F-Cyc all up-regulated genes overrepresented for protein biosynthesis terms (Fig. 2B), as reported previously (*9*, *10*), aligning with the dendrogram analysis (Fig. 1D). F-Act did not show this signature, highlighting its unique identity. The top GO BP terms characterizing F-Act were ECM organization and collagen fibril organization, terms overlapping with F-CI and F-Cyc (Fig. 2B). As expected, F-Cyc was uniquely characterized by GO BP terms related to mitotic cell cycle progression. These data and trajectory analyses reinforce the close relationship between IR and F-CI with respect to protein biosynthetic state and commitment to proliferation.

We also calculated DEGs and GO BP terms between activated states (F-Act, IR, F-CI, and F-Cyc) at MI-days 1 to 3, comparing each to the remainder (Fig. 2C and fig. S3, C and D). In contrast to the protein biosynthetic signature observed for IR when compared to resting fibroblasts, the unique GO BP signatures for IR were predominantly related to regulation of apoptosis, including high overlap with negative regulation of cell death and responses to stress terms (Fig. 2C and fig. S3C), as well as diverse prosurvival, growth, and metabolic genes, including *Tgfb*, *Igfbp3*, *Ncl*, *Eif4a1*, and *Tomm40* (table S2), suggesting that IR up-regulates transcriptional programs that protect against apoptosis and stress. Cell death, wound healing, and cell migration terms were shared between IR, F-Act, and F-CI (Fig. 2C), indicating broader activation of these programs. Several terms related to nucleotide/nucleoside synthetic pathways were overrepresented in F-CI, consistent with its close relationship to proliferating CF.

To assess the temporal dynamics of the above states more broadly, we created Seurat module scores for DEGs covering the most significant GO BP terms relevant to the four early activated populations (IR, F-CI, F-Cyc, and F-Act) discussed above. The module score for regulation of programmed cell death, primarily associated with IR (Fig. 2C), increased from healthy hearts to a peak at MI-day 1, before decreasing to near healthy levels at later MI time points (Fig. 2D and fig. S3E). Scores for protein biosynthesis/translation terms, features of IR, F-CI, and F-Cyc (Fig. 2B), showed a similar pattern, peaking at MI-day 1 (Fig. 2D). By contrast, module scores for ECM organization and tissue development, most strongly associated with F-Act and F-CI (Fig. 2C and fig. S3F), showed a gradual increase to MI-day 7 (after the peak of MYO) followed by a decline (Fig. 2D). The tissue development term, mostly containing genes for ECM (*Col1a1*, *Col1a2*, *Cthrc1*, *Tnc Lox*, *Loxl3*, *Timp1*, *Sdc1*, and *Fn1*), cytoskeleton (*Acta2*, *Palld*, *Actg1*, *Tpm1*, and *Tagln*), and secreted factors (*Spp1*, *Cxcl12*, and *Ptn*), showed a similar trend.

### Origin and fate of IR

In our integration study, IR was limited principally to MI-day 1. However, in their original study, Forte *et al.* (*10*) found persistence of IR at MI-days 3 and 5 (~19 and 16% of total CFs, respectively) and expressing progressively higher levels of MYO genes including *Acta2 and Cthrc1*. It was inferred that IR rapidly transitions to MYOs at MI-day 3. However, our previous work and this integration study have shown that differentiated MYOs do not substantially accumulate until MI-day 5 (Fig. 1F) (*8*, *9*).

To explore IR origin and fate in greater depth, we extracted cells originally classified as IR from our unbiased reclustering of the Forte *et al.* data (*10*) and regenerated a UMAP plot with cell identities based on integration analysis. This revealed two well-separated populations (Fig. 2E) with segment 1 (larger; left) comprising primarily cells that retained the IR identity and minor proportions of F-SH, F-SL, F-Trans, and F-Act. Segment 2 (smaller; right) comprised cells reclassified here as F-CI, F-Cyc, or MYO, with only rare IR cells. Segment 1 (mostly IR) contained cells from MI-day 1, whereas segment 2 was specific to MI-days 3 and 5 (Fig. 2F and fig. S3G). Cells at MI-day 3 were mostly F-CI, whereas MYO cells were generated predominantly at MI-day 5. Early IR-enriched markers including *Mt2* and *Angptl4* were up-regulated in segment 1 and substantially down-regulated in segment 2, whereas ECM and MYO-related markers, including *Cthrc1* and *Fn1*, were absent in segment 1 and up-regulated in segment 2 (Fig. 2G). Thus, our integration approach can more accurately assign CF states. In our refined data, IR is largely restricted to MI-day 1, corresponding to the peak of neutrophils (*41*), and is clearly distinct from MYO. Cells classified as IR on MI-day 3 corresponded mostly to CFs primed to undergo proliferation, represented by F-CI and F-Cyc in our data. MYO appeared predominantly at MI-day 5, consistent with the larger time course and trajectory analyses (Figs. 1C and 2A).

### Commitment to MYO differentiation

Given the complex transitions between resting and early activation states, we next asked if proliferative phase cells (IR, F-CI, and F-Cyc) have committed to an MYO fate or if they act as transit amplifying cells. To address this, we explored the dynamics of markers for fibroblast activation (*Meox1*) and myofibrogenesis (*Acta2*, *Cthrc1*, *Col1a1*, and *Scx*) across multiple MI time points in our integrated data (fig. S4 and table S3). *Meox1*, although known as a key early driver of CF activation (*51*), was expressed across different resting and activated CF populations (fig. S4, A and B) and was therefore uninformative. *Scx*, encoding the profibrotic transcription factor (TF) Scleraxis (*52*), was lowly expressed in the integrated data. However, a notable finding was that *Acta2* and *Cthrc1* were expressed at much higher levels in F-CI and F-Cyc compared to resting CFs and F-Act, from MI-days 3 to 7 [fig. S4A and table S3; MAST testing; *P*_adj_ < 1 × 10^−05^; log_2_(fold change) > 0.5]. This was also not apparent in IR. *Cthrc1* was expressed in 88 and 68% of F-CI and F-Cyc, respectively, and *Acta2* in 90 and 79%, respectively (table S2). *Col1a1* expression was also higher in F-CI and F-Cyc than in resting CF and F-Act and was even lower in IR. Peak mean levels of *Acta2* in F-Cyc at MI-day 5 were almost as high as in MYO at the same time point, although levels had declined by MI-day 7 in both F-Cyc and MYO. Levels of *Cthrc1* in F-Cyc also peaked at MI-day 5 before declining by MI-day 7; however, overall levels in F-Cyc remained lower than in MYO. We conclude that most F-CI and F-Cyc cells show hallmarks of commitment to myofibrogenesis at the single-cell level, a feature not shared by IR. Commitment was apparent at MI-day 3, prior to the appearance of substantial numbers of MYO (Fig. 1, C and F). These findings are consistent with previously documented ACTA2/α-SMA immunostaining in lineage-traced CFs as early as MI-day 3, correlating with peak CF proliferation (*46*).

### TF networks regulating CF states and transitions

To infer TFs that regulate CF states and state transitions, we performed TF network analysis on the integrated MI data using decoupleR (*53*) and identified the top predicted factors for populations and time points of interest. A heatmap of weighted TF activity scores at early MI time points (days 1 to 3) (fig. S5 and table S4) showed notable segregation of injury-related CF populations (IR, F-CI, and F-Cyc) from homeostatic CFs. Injury-induced populations were highest for TFs related to cell proliferation, self-renewal, and cancer involving both intrinsic (E2F family, MYC, and ZFX) and extrinsic (HIF1a, EPAS1/HIF2a, NFYB, LEF1, GLI2, VDR, and SMAD1/3/5) pathways. Nontypical examples included ZFX, a self-renewal TF, which is highly expressed in tumors and binds across the genome to thousands of CpG island promoters, many of which regulate cell cycle and cancer-related genes (*54*, *55*). NFY proteins, which contain a histone-like domain, bind to CCAAT motifs, also found commonly in promoters, allowing scaffolding of many cofactors to regulate a host of genes important for cell cycle transitions (*56*, *57*). IR, F-CI, and F-Cyc each showed a unique pattern of TF regulon activity—some in common (MYC, GLI2, VDR, and ZFX), others higher in F-Cyc (KLF5, LEF1, and E2F1/3) or IR (WT1, MBD1, and SNAPC4), with others such as E2F2/4, TFDP1, and NFYB showing a graded pattern (F-Cyc > F-CI > IR). This heterogeneity reinforces the different commitment states of early injury populations with respect to cell cycle and myofibrogenesis, as described above. The vitamin D receptor (VDR) pathway was observed in all injury-related CFs with evidence from liver showing that it may function as part of a negative feedback loop limiting fibrosis by repressing genomic SMAD3 targets (*58*). HIF1α and HIF2α/EPAS1 signatures, in contrast, were high in IR and F-CI but low in F-Cyc, consistent with our findings that HIF1α provides a strong braking mechanism for CF proliferation at MI-day 1 through regulation of antioxidant pathways (*8*). KLF5 activity was highest in F-Cyc and is known to be involved in multiple CV disease processes and associated lineages, including pressure overload–related fibrosis (*59*).

TF regulons showing higher activity in homeostatic fibroblast populations (F-SH, F-SL, F-Trans, F-WntX, and F-Act) were variously related to stem cell and cancer states, chromatin regulation, epithelial-mesenchymal transition, multilineage differentiation and morphogenesis, signaling, and metabolism. The lower activity of these factors in injury-related CFs (IR, F-Cyc, and F-CI) may limit their differentiation into MYOs and alternative cell fates. The analysis of resting CF populations also revealed pathways for the less characterized F-WntX and F-Trans (figs. S5 and S6), reinforcing their separate albeit related identities (Fig. 1B).

Network analysis of populations present at late MI time points (days 7 to 30; fig. S6 and table S4) revealed profibrotic pathways higher in MYO and diminished in MFC, including those for SMAD3, MYCN, GLI2, TEAD4, and GATA3. It is noteworthy that transcripts for most TFs highlighted above are expressed at very low levels and not enriched in any specific CF subpopulations. Therefore, these TF analyses reveal levels of network control not apparent from gene expression alone and highlight a host of TF regulons controlling CF cell states and transitions.

### Profibrotic and antifibrotic MYOs and MFCs

**Fig. 3.**
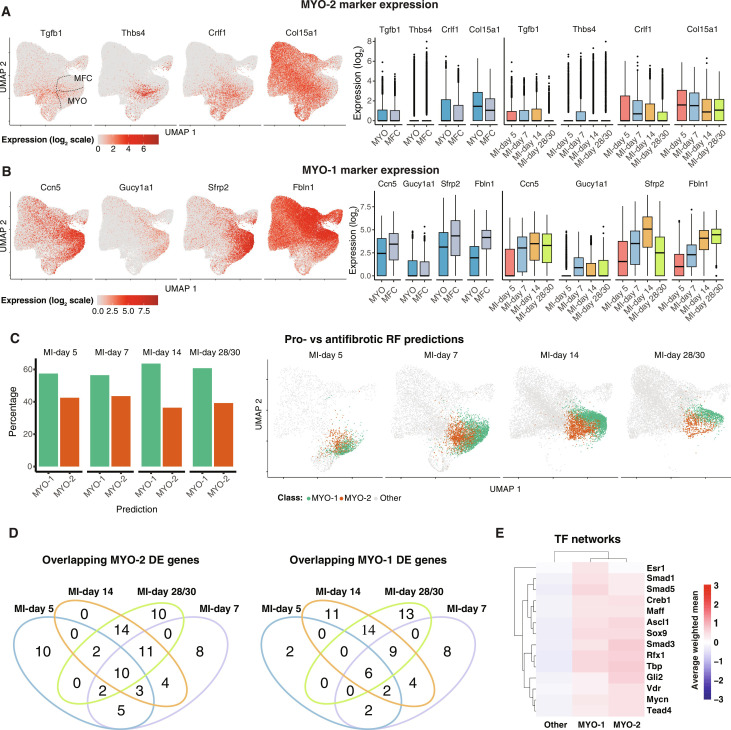
Profibrotic versus antifibrotic MYOs and MFCs. (**A** and **B**) Expression of marker genes for MYO-2 [(A) profibrotic] or MYO-1 [(B) antifibrotic] on an aggregate UMAP or as visualized in box plots comparing MYO and MFC or an aggregate of MYO and MFC across the relevant MI time points. (**C**) Percentage of MYO/MFC cells across the indicated MI time points predicted with a RF classifier corresponding to either antifibrotic versus profibrotic subtypes. Shown are the percentage of cells predicted in either category (left) or the location of the predicted pro/antifibrotic cells on UMAP coordinates according to time point (right). (**D**) Venn diagrams representing flux of MYO-1 and MYO-2 differentially expressed signature genes [MAST testing; *P*_adj_ < 1 × 10^−05^; log_2_(fold change) > 0.5] across treatment conditions. (**E**) Top 10 predicted TFs for MYO-1 or MYO-2 cells for MI-days 5 to 30 cells. Shown are the average weighted mean decoupleR scores across the cells for each indicated population.

We have previously reported that MYO at MI-day 7 can be subdivided into populations that exhibit profibrotic or antifibrotic transcriptional signatures, which we referred to as MYO-2 and MYO-1, respectively (*9*). In our integrated data, we asked if the ratio of profibrotic and antifibrotic subsets changed over time in a way that might illuminate their relationship. We first interrogated data for the top previously defined marker genes that discriminate profibrotic versus antifibrotic subsets (*9*), which, for profibrotic cells, included *Tgfb1*, *Thbs4*, *Crlf1*, and *Col15a1* and, for antifibrotic cells, *Ccn5/Wisp2*, *Sfrp2*, *Gucy1a1*, and *Fbln1*. Notably, UMAP plots suggested the existence of profibrotic and antifibrotic components within both MYO and MFC (Fig. 3, A and B). Profibrotic MYO-2 markers were expressed across the junction of MYO and MFC in UMAP space and at comparable levels in each compartment, as well as over MI time (Fig. 3A). Antifibrotic markers *Ccn5*/*Wisp2* and *Sfrp2*, previously suggested to be up-regulated in MFCs (*10*), were higher in MFC versus MYO in the integrated data [table S5; MAST testing; *P*_adj_ < 1 × 10^−05^; log_2_(fold change) > 0.5], albeit that they were expressed in all MI time points (Fig. 3B), also true for other MYO-1 markers *Gucy1a1* and *Fbln1*.

To clarify the dynamics of MYO-1 and MYO-2 further, we used the predefined MYO-1 and MYO-2 subsets from the original *Pdgfra*-eGFP^+^ (enhanced green fluorescent protein–positive) MI-day 7 CF data of Farbehi *et al.* (*9*) to train a Random Forest (RF) classifier to predict MYO-1 and MYO-2 on the integrated data (Fig. 3C). We confirmed a comparable proportion of predicted MYO-1 and MYO-2 substates as early as MI-day 5 (Fig. 3C), when MYO first accumulated in substantial numbers (Fig. 1C), through to MI-days 28/30 when MFCs were dominant, and MYOs were almost completely resolved (Fig. 3C). Thus, profibrotic and antifibrotic substates may exist within both MYO and MFC (Fig. 3, A to C).

After time point–specific comparisons, 71 and 79 DEGs, respectively, distinguished MYO-1 and MYO-2 states across MYO and MFC subtypes (table S5). Expression of these genes changed over time, although 33 of the MYO-1 signature genes were expressed in at least two time points, and 47 of MYO-2 signature genes (Fig. 3D). Canonical genes for MYOs, such as *Acta2*, *Cthrc1*, and *Col1a1* were not differentially expressed between MYO-1 and MYO-2 states, reinforcing their essential MYO identity. However, other ECM-related genes including *Cilp*, *Eln*, *Fbln1*, *Timp1*, *Col15a1*, *Col4a1*, and *Col4a2* were differentially expressed at most time points (table S5), suggesting functional differences between these states (see Fig. 3, A and B). The up-regulation of *Eln* (encoding elastin) in MYO-1 states suggests that these cells generate a more elastic matrix.

Last, we compared TF pathways defining the antifibrotic MYO-1 and profibrotic MYO-2 subpopulations found within MYO and MFC (Fig. 3E). Regulon patterns were largely overlapping; however, profibrotic MYO-2 showed higher activity scores for VDR, GLI2, and SMAD3 pathways, whereas antifibrotic MYO-1 was depleted of these and elevation of ESR1 (estrogen receptor) and SMAD1/5 pathways. As mentioned, VDR may be part of a negative feedback loop limiting SMAD3-mediate fibrosis (*58*), and the GLI2 pattern likely reflects activation of the canonical hedgehog pathway, which has pleiotropic cardioprotective effects (*60*). Although the cardioprotective roles of estrogens in women are well known, studies in *ESR1* knockout mice also demonstrate CV protective effects in males (*61*). Our data suggest estrogens and the balance of bone morphogenetic protein and transforming growth factor–β (TGFβ) SMADs as positive drivers in the resolution of MYO and MFC to antifibrotic states.

### Integration of cross-disease CF datasets

**Fig. 4.**
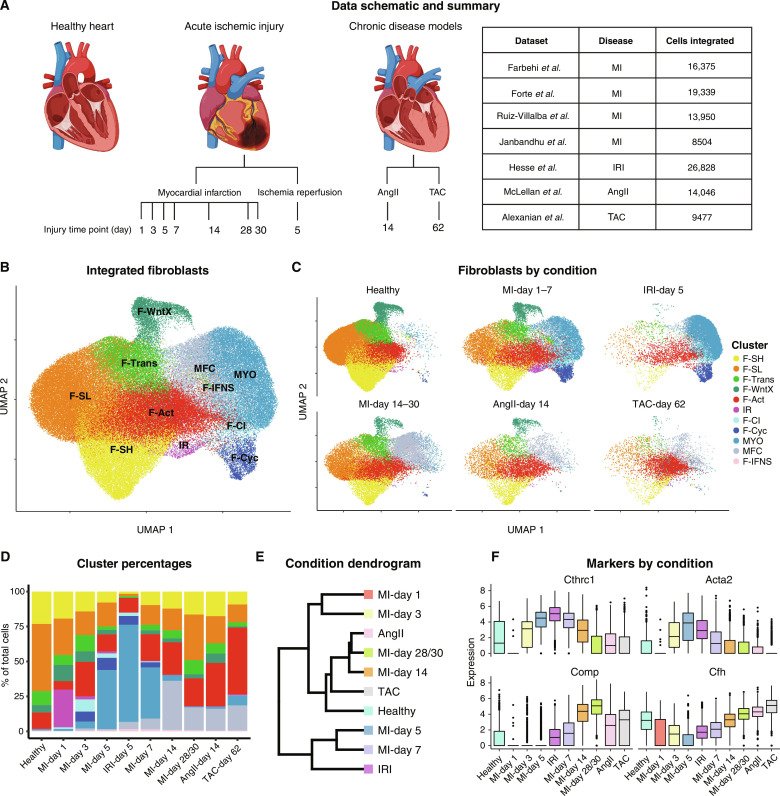
Cross-disease integration. (**A**) Schematic of the disease and time points used for integration and the corresponding studies. (**B**) UMAP plot showing an aggregate of CFs across conditions. (**C**) UMAP plot showing CFs according to condition. (**D**) Percentage of CFs in each population according to experimental condition. (**E**) Dendrogram of disease conditions determined by average batch-corrected expression in each condition across populations. (**F**) Expression of select markers according to disease condition.

Although differentiated CF states including MYO and MFC have been characterized in MI, it remains unclear to what extent these exist in different disease contexts. To address this, we expanded our MI map to include available CF scRNA-seq data from three additional disease models comprising single late-disease time points for ischemia-reperfusion injury (IRI) (day 5) (*62*), as well as angiotensin II (AngII)–induced and trans-aortic constriction (TAC)–induced hypertension associated with cardiac hypertrophic cardiomyopathy (days 14 and 62, respectively) (*51*, *63*)—in humans, chronic disease states reflecting heart failure with reduced ejection fraction (HFrEF) (Fig. 4A). Again, we used cFIT to transport these diverse experiments into an integrated space and generated UMAP plots using the low-dimensional coordinates returned. To interpret these models in the light of defined MI subtypes, we trained an RF classifier on the integrated gene expression data using cell type labels determined above and applied it to the IRI, AngII, and TAC datasets (Fig. 4, B to D, and fig. S7, A to C). We confirmed these identities in the AngII, TAC, and IRI experiments using Seurat label transfer analysis (*26*) as a complimentary approach (fig. S7, D to F). Cells from IRI-day 5 showed high similarity to MI-days 1 to 7, with a predominance of MYO followed by F-Act (Fig. 4, C and D), confirmed by dendrogram analysis (Fig. 4E). IRI-day 5 showed a much higher relative prevalence of MYO than any MI stage (Fig. 4D and fig. S7C).

In contrast, very few MYO cells were predicted at late stages of AngII or TAC models (Fig. 4, C and D), consistent with previous observations (*63*). MYOs were similarly absent in a separate TAC study at days 14 and 28 after surgery (*47*). Rather, we found that the AngII and TAC profiles showed similarity to late stages of MI (Fig. 4E), with induced populations corresponding to MFCs and an expansion of F-Act (Fig. 4, C and D). In the initial report on the AngII model using scRNA-seq (*63*), the *Thbs4*-high population corresponds to MFC and the *Cilp*-high population corresponds to F-Act in the integrated data. Expression of MYO markers *Cthrc1* and *Acta2* were down-regulated in AngII and TAC hearts compared to MI-days 5 and 7, whereas *Comp*, a highly specific marker of MFC, and another enriched MFC marker, *Cfh* (encoding complement factor H), were up-regulated [Fig. 4F and table S6; MAST testing; *P*_adj_ < 1 × 10^−05^; log_2_(fold change) > 0.5]. *Thbs4* and *Cilp* encode ECM proteins with diverse roles in ECM structure and remodeling (*64*, *65*). In our integrated data, *Cilp* was expressed in a complex and graded patterns across F-Act, MYO, and MFC (fig. S8, A and B). *Thbs4* expression was restricted to F-Act in IRI and a subset of MYO and MFC cells (fig. S8, A and C), the latter corresponding to the profibrotic compartment described above (Fig. 3A). Thus, these markers are suggestive but not diagnostic of CF subtypes, whereas our integration approach has allowed us to identify the specific subpopulations induced by pressure overload.

### Origins of MFC in the AngII model

**Fig. 5.**
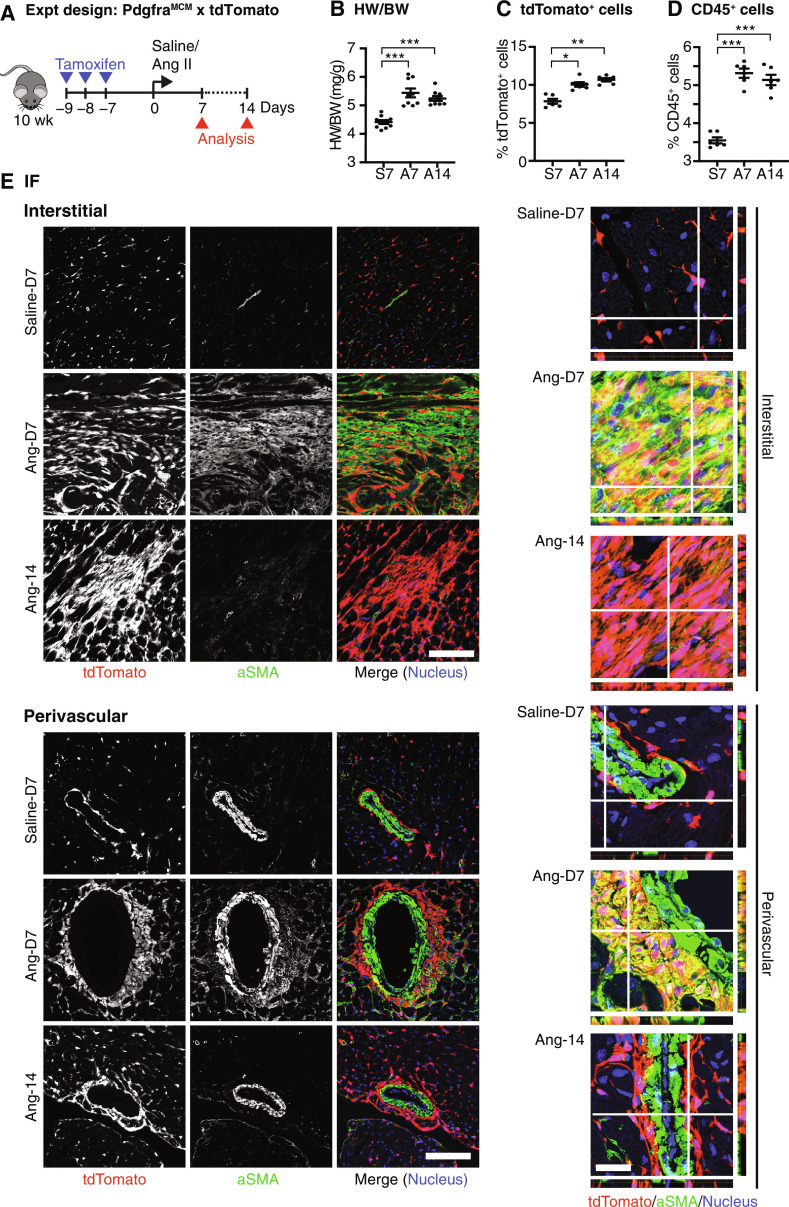
MFCs are derived from MYOs in AngII model. (**A**) Schematic of the experimental (Expt) design. (**B**) Heart weight–to–body weight ratio (HW/BW) following AngII administration (*n* = 9 to 10). (**C** and **D**) Percentage of tdTomato^+^CD31^−^CD45^−^ (C) and CD45^+^ (D) cells (E) in total interstitial cell population (*n* = 6). (**E**) Representative immunofluorescence images of heart sections showing staining for indicated antigens. Scale bars, 100 μm. Orthogonal slice views are shown on the right. Scale bar, 25 μm. Data from 9 to 10 biological replicates are presented. Bars represent the means ± SEM. *P < 0.05, **P < 0.01, and ***P < 0.001 by ANOVA.

The presence of abundant MFC in the absence of MYO in AngII and TAC models (days 14 to 62) begs the questions of whether MYO are generated at earlier time points or whether there are distinct cell trajectories to MFC (Fig. 2A). Although the presence of collagen deposition in pressure overload–mediated hypertrophic heart failure is well established (*4*, *66*), evidence supporting substantial focal MYO formation using α-SMA or other markers, is less conclusive (*43*, *47*, *67*–*71*). It has been reported that CF activation and collagen deposition in diabetic and pressure overload cardiomyopathy occurs in the absence of MYO differentiation (*32*, *47*, *63*, *72*). Here, we confirmed that, using genetic lineage tracing, AngII hearts show early focal but transient myofibrogenesis. To mark CFs, we crossed *Pdgfra^MCM/+^* tamoxifen-dependent Cre driver mice with Cre-dependent tdTomato reporter mice (*8*) and introduced AngII via mini-pump in young adults, with analysis at 7 and 14 days (Fig. 5A). Increased heart weight/body weight ratio and tdTomato^+^ cells and influx of CD45^+^ immune cells at early and late time points confirmed induction of cardiomyocyte hypertrophy and chamber remodeling (Fig. 5, B to D). Using immunofluorescence on mid-ventricular sections, fibrosis manifested at day 7 as the presence focally of large tdTomato^+^α-SMA^+^ CFs in interstitial and periarteriolar locations associated with collagen deposition (Fig. 5E and fig. S9). Large tdTomato^+^ fibroblasts were also present at interstitial and periarteriolar locations at day 14; however, these were α-SMA^low-negative^, consistent with an MFC identity. These data demonstrate that there is a transient wave of myofibrogenesis in AngII hearts around day 7, which is resolved by day 14. We hypothesize that MFCs present at late stages of pressure overload cardiomyopathy derive principally from MYOs present at earlier stages, as in MI. Although the angiotensin stimulus was delivered constantly over 14 days, MYO was nonetheless resolved in favor of MFC, suggesting that MYO is self-limiting in vivo.

### DEGs and transcript isoform use in MFC across different disease models

**Fig. 6.**
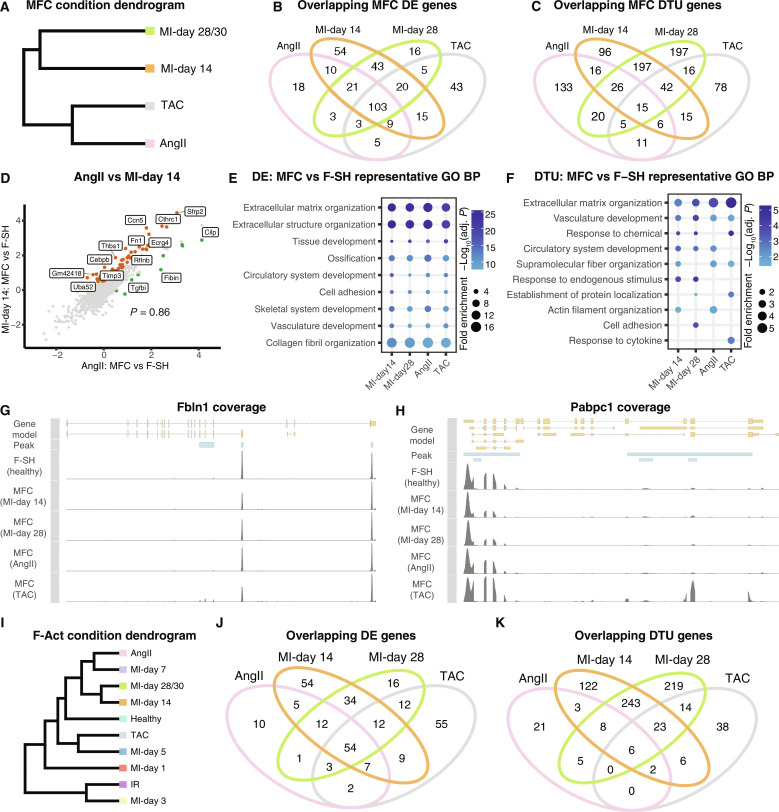
Cross-disease comparison of MFCs and activated fibroblasts. (**A**) Dendrogram of indicated disease conditions determined by average batch-corrected expression in MFCs. (**B** and **C**) Overlapping DE [MAST testing; *P*_adj_ < 1 × 10^−05^; log_2_(fold change) > 0.5] (B) and DTU [Sierra; *P*_adj_ < 0.05; log_2_(fold change) > 0.5] (C) genes between conditions determined by comparing MFCs to the healthy F-SH cells of the relevant dataset. (**D**) Comparison of log_2_(fold changes) comparing MFC to F-SH for AngII and MI-day 14. (**E** and **F**) Representative GO BP terms for MFC versus F-SH (E) DE or DTU (F) genes between conditions. (**G** and **H**) Read coverage plots for example DTU genes (G) *Fbln1* and (H) *Pabpc1* with the differential peak indicated. Shown is read coverage for F-SH from a merge of healthy hearts compared to MFC cells from the indicated conditions. (**I**) Dendrogram of disease conditions determined by average batch-corrected expression in F-Act. (**J** and **K**) Overlapping DE (J) and DTU (K) genes between conditions determined by comparing F-Act to the healthy F-SH cells of the relevant dataset.

Because MFCs are predicted to accumulate in both ischemic (MI/IRI) and pressure overload (AngII and TAC) models, we explored if molecular signatures discriminate MFC states in different injury settings. Unbiased dendrogram analysis showed that the two late MI time points representing peak MFC accumulation (days 14 and 28/30) clustered together, however, separately from AngII and TAC MFC (Fig. 6A).

We calculated DEG and differential transcript usage (DTU) [Sierra (*44*); *P*_adj_ < 0.05 and log_2_(fold change) > 0.5] genes for each condition by comparing MFC states to resting F-SH of respective healthy controls (Fig. 6, B and C, and table S7). There was a strong overlap of DEGs for MFC between conditions, with 28% (103/368) common to all four conditions and 43% common to at least three conditions (Fig. 6B). Relatively few genes (~4 to 15%) were unique to one condition. Correlating expression fold changes (comparing MFC versus F-SH) revealed that the transcriptomes of MFC in the AngII model were highly positively correlated to those in MI (*P* = 0.86; Fig. 6D). We conclude that the identity signature of MFC is highly stable between MI and hypertrophic disease models.

In contrast, there were a greater number of DTU genes unique for each condition, with only 1.7% (15/873) found in common (Fig. 6C). For example, for the AngII model, 57% (133/232) of DTU genes were unique, in contrast to 10% (18/172) unique DEGs (Fig. 6B). Although the highest similarity was between MI time points, with 280/651 (43%) of total MI DTU genes shared between MI-days 14 and 28, most of these (70%) were unique to the MI condition (Fig. 6C).

Supporting the above, GO term analysis showed consistency of BPs for MFC DEGs across conditions but more divergence for DTU genes (Fig. 6, E and F, and fig. S10, A and B). Top DE terms included extracellular matrix organization, extracellular structure organization, collagen fibril organization and ossification, and skeletal system development (Fig. 6E), mirroring previously observed osteogenic and chondrogenic signatures in MFC (*46*). These terms were relatively consistent in fold enrichment and adjusted *P* value across conditions. In contrast, top GO terms for DTU genes reflected time- and/or model-dependent network changes (Fig. 6F and fig. S10B). Among DTU genes was *Fbln1*, encoding fibulin 1, an ECM glycoprotein involved in cell migration, proliferation, and differentiation, and essential for heart morphogenesis (*73*). The long *Fbln1* isoform uniquely contains a conserved fibulin-specific domain of unknown function and is up-regulated in MFC compared to F-SH (Fig. 6G). Another DTU gene, *Pabpc1*, encodes polyadenylate [poly(A)]–binding protein cytoplasmic 1, which promotes ribosome recruitment and translation initiation, and poly(A) tail shortening (*74*) and is important for the increased translation seen during cardiac hypertrophy (*75*). *Pabpc1* expresses multiple annotated transcript isoforms, which showed massively different usage across conditions (Fig. 6H). In summary, the MFC cell state is stable across disease models and time points, albeit that they respond uniquely to the specific injury environments through differential isoform use.

### Molecular signatures of F-Act across disease states and time points

We asked if F-Act, present throughout the injury time courses, also adapts to the injury environment in different disease models, as for MFC. In dendrogram analysis of F-Act cells across conditions, MI-days 14 and 28/30 showed the highest similarity; however, F-Act cells in AngII and TAC models were not aligned with each other, rather with MI-day 7 and MI-day 5, respectively (Fig. 6I). We again performed DE and DTU analysis comparing F-Act to F-SH of cognate healthy controls (table S8). Compared to MFC, there were relatively fewer DE and DTU genes for F-Act versus F-SH across different states, reflecting their closer relationship. Nonetheless, there was a similar trend with more overlapping DEGs and more unique DTU genes (Fig. 6, J and K). For example, in the AngII model, only 11% (10/94) of the F-Act DEGs were unique to MI, whereas 46% (21/45) of DTU genes were unique. Combining time points, 31% (70/219) of DEGs were unique to MI, compared to 52% (341/651) unique DTU genes. *Rhoa*, encoding ras homology family member A, implicated in the progression of pathological hypertrophy in TAC (*76*), exhibited preferential expression of a shorter 3′UTR in F-Act (fig. S11A).

GO BP analyses of F-Act genes at different time points across conditions revealed a large shift in terms of the transition from healthy hearts to disease states (fig. S11B). MI-day 1 showed the more unique disease profile, consistent with the dendrogram analysis (Fig. 6I). F-Act at MI-day 1 showed increased expression of IR markers such as *Mt1*, *Mt2*, and *Angptl4* (fig. S11, C and D). Consistently, DEGs for IR and F-Act at MI-day 1 were highly correlated (*P* = 0.81; fig. S11E). These data likely reflect the proposed dynamic flux between F-Act and IR states. F-Act GO BP terms were relatively unchanged between MI-days 3, 5, and 7, indicating that this population remained stable thereafter and did not progress toward MYO with time.

### CF states in heart failure with preserved ejection fraction

Heart failure with preserved ejection fraction (HFpEF) is the most common form of heart failure, showing heterogeneous clinical features, high burden of common comorbidities such as advanced age, cardiometabolic stress, hypertension, and multiorgan fibrosis (*77*). However, to what degree fibrosis correlates with adverse outcomes or is causative in HFpEF remains unexplored (*4*). To examine this, we used a recently described two-hit HFpEF model in C57Bl/6J mice over 15 weeks, involving high fat diet (HFD) with concomitant induction of hypertension by delivery of a selective inhibitor of nitric oxide synthases, *N*ω-nitro-l-arginine methyl ester (1+) (l-NAME) (fig. S12A) (*78*, *79*). We confirmed increased blood pressure, preserved left ventricular ejection fraction, diastolic dysfunction (increased E/e′), and decreased global longitudinal strain by echocardiography, as well as increased lung weight and cardiomyocyte hypertrophy without changes in vessel density (fig. S12, B to H, and fig. S13A). Furthermore, tandem mass tag-based proteomics of HFpEF versus chow-fed ventricle samples confirmed significant up-regulation of PDK4 and PERILIPIN5 (PLIN5) and overall changes indicative of decreased utilization of glucose, amino acids, and ketone bodies for energy and increased lipid accumulation (fig. S13, B and C), consistent with a study on murine and human HFpEF (*79*).

We performed scRNA-seq of total cardiac ventricular interstitial cells from HFpEF and chow-fed mice at 10 and 15 weeks (*n* = 2). This revealed no significant changes in CF or other cell type proportions (fig. S12, I and J) and no MYO was evident. MFCs occurred in small numbers, although they were not increased in the HFpEF hearts. Consistently with lack of fibrosis, there was no evidence of increased fibrillar collagen deposition in the ventricles or atria using picrosirius red staining in the C57Bl/6J model (fig. S13D). The number of DEGs between conditions [*P*_adj_ < 0.05; log_2_(fold change) > 0.5] was modest and were found mostly in CFs at 15 weeks (fig. S13E and table S9). Notably, although fibrosis was not evident, several fibrosis-related genes including *Il1b*, *Postn*, *Fn1*, *Tgfb1*, *Acvr1b*, *Ccn2*, *Runx1*, and *Runx2* were slightly up-regulated at 15 weeks in resting fibroblasts (F-SH and F-SL), with the antifibrotic gene *Igf1* down-regulated (fig. S13F), suggesting early stages of an emerging fibrotic phenotype. Top GO terms for DEGs related to signal transduction and regulation of reactive oxygen species (table S9), the latter specific to F-SH cells and involving up-regulation of *Nox4*, *Pdk4*, *FoxO3*, *CD36*, *Trim30a*, and *Ccn2* (table S9). We conclude that metabolic dysregulation is the major driver of HFpEF in the C57Bl/6J model.

### Conservation of mouse CF states in human hearts

**Fig. 7.**
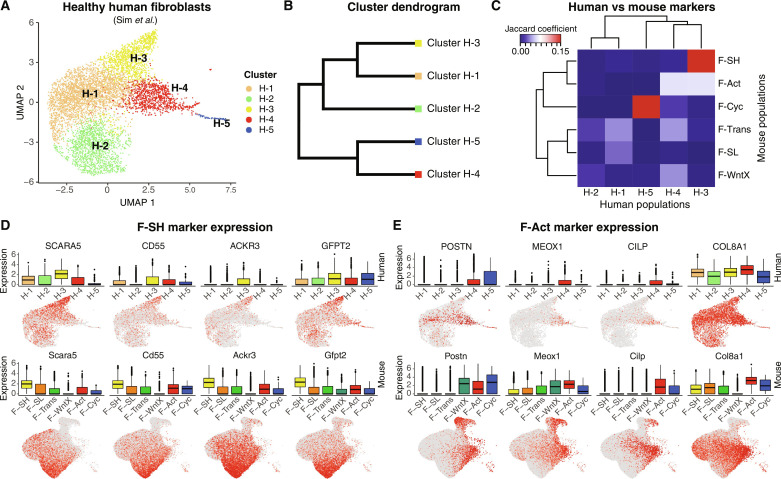
Cross-species comparison of CFs between human and mouse. (**A**) UMAP of human CFs with identified subclusters. (**B**) Dendrogram of human CF clusters determined by average integrated expression in populations. (**C**) Jaccard coefficients for the overlap of human and mouse CF marker genes [MAST testing; *P*_adj_ < 1 × 10^−05^; log_2_(fold change) > 0.5] from uninjured hearts. (**D** and **E**) Gene expression as visualized in box and UMAP plots comparing human (top) and mouse (bottom) fibroblasts for (D) F-SH markers and (E) F-Act markers.

We asked if CF states defined in murine models are conserved in human hearts by comparing our mouse integrated map to a recent single-nuclei RNA-seq (snRNA-seq) dataset on left ventricles of three healthy (donor) human hearts (*80*). After quality control (QC) filtering and clustering, we selected fibroblasts for in silico analysis. Reclustering yielded four abundant healthy (H) populations (H-1 to H-4) and one minor proliferative population (H-5) (Fig. 7, A and B). We calculated marker genes [table S10; MAST testing; *P*_adj_ < 1 × 10^−05^; log_2_(fold change) > 0.5] for each cluster and compared them to state-specific markers from integrated data of healthy murine hearts. Human heart cluster H-3 showed a strong correlation with mouse F-SH, which, as noted above, is the major quiescent CF population enriched in stem cell markers. Cluster H-4 (and H-3) showed a significant relationship to mouse F-Act, albeit with a lower Jaccard score (Fig. 7C). We could discriminate clusters H-3 and H-4 on the basis of differential expression of top F-SH and F-Act markers genes—cluster H-3 up-regulated mouse F-SH markers including *SCARA5*, *CD248*, *ACKR3*, and *GFPT2* (Fig. 7D) (*9*, *10*, *81*), whereas cluster H-4 up-regulated top mouse F-Act markers including *POSTN*, *MEOX1*, *CILP*, and *COL8A1* (Fig. 7E). A *SCARA5^+^* population (H-3) has also been seen in human infarcted hearts (*21*). Proliferating CFs from mouse and human hearts were also highly correlated. The significance of associations was confirmed by Fisher’s exact tests (fig. S14A). In summary, two of the main murine CF populations, F-SH and F-Act, and proliferating CFs (F-Cyc) show cognate cell populations in healthy human hearts, suggesting conservation of both progenitor-like and activated CF states. Specific human homologs for other mouse CF populations (F-SL, F-WntX, and F-Trans) were not found in these data (fig. S14B).

**Fig. 8.**
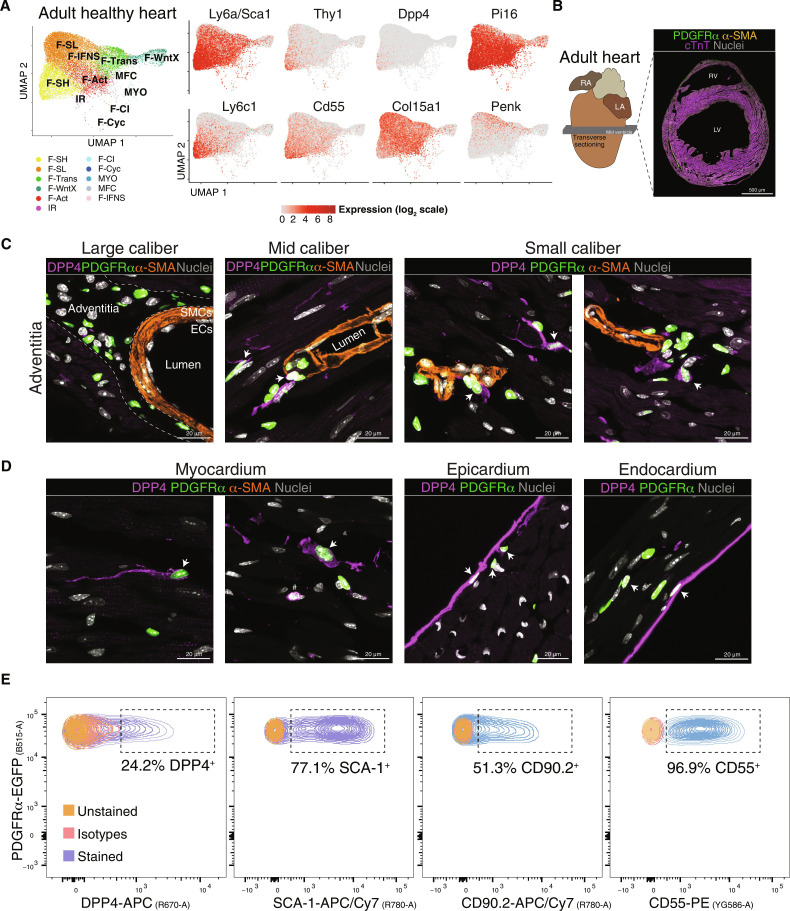
Localization of *Pdgfra*-eGFP^+^DPP4^+^ fibroblasts in myocardium vascular adventitia and interstitium. (**A**) UMAP of fibroblasts from healthy hearts. Expression of select genes in different CF populations as visualized in UMAP plots is shown on the right with population identities shown on the left. (**B**) Sectioning outline and example of a mid-ventricular heart section of adult *Pdgfra*^eGFP/+^ knockin mice stained for indicated antigens. Scale bar, 500 μm. (**C** and **D**) 9 μm *Z*-stack high-resolution confocal images showing staining of indicated antigens in the perivascular zone of adventitial layer of large to small vessels (C) and myocardium, epicardium, and endocardium (D). PDGFRα corresponds to nuclear eGFP fluorescence. RV, right ventricle; LV, left ventricle; RA, right atrium; LA, left atrium; SMCs, smooth muscle cells; ECs, endothelial cells (*n* = 2). Scale bars, 20 μm. (**E**) Spectrum of live fibroblasts from mouse ventricles labeled with DPP4, SCA-1, CD90.2, and CD55 antibodies, after selection for *Pdgfra*-eGFP^+^ cells. Unstained and isotype control cells were included to define the gating strategy (*n* = 3).

In a recent single-cell multiorgan and mouse-human comparison, two fibroblast clusters were proposed as “universal” (common across organs in mice and humans) (*82*). The universal *Pi16*^high^ cluster appears homologous to the mouse F-SH clusters based on the shared expression of *Pdgfra*, *Ly6a*/*Sca1*, *Pi16*, *Dpp-4*, *Ly6c1*, *Cd34*, *Cd55*, *Scara5*, and other markers (*9*, *82*). This cluster has been proposed to be related to vascular adventitial cells. The other universal cluster reported (*Col15a1*^high^) was assigned as basement membrane–secreting parenchymal CFs, expressing higher levels of *Col15a1* and *Penk*. We displayed relevant cognate markers in UMAP plots of healthy mouse heart ventricle integrated data (Fig. 8A). The common feature of F-SH/*Pi16*^high^ cluster was graded expression of discerning markers across UMAP space with highest levels in F-SH; however, these markers were also expressed more sparsely and at lower levels in other CF populations including F-Act, F-SL, and F-Trans. The *Col15a1*^high^ cluster markers (*Col15a1* and *Penk*) were also graded but antithetically to F-SH/*Pi16*^high^ markers, with lowest levels in F-SH. To spatially localize F-SH cells in mouse hearts, we chose DPP4 as one of the most selective F-SH markers (Fig. 8A). DPP4 was reported previously to be expressed in multilineage adipofibrogenic mesenchymal progenitors in subcutaneous periadipogenic and adventitial connective tissue (*83*). Using immunofluorescence, we detected DPP4 in a subset of *Pdgfra*-eGFP^+^ reticular dermal fibroblasts, as expected (*83*), as well as *Pdgfra*^+^ dermal papilla fibroblasts, a hair follicle stem cell population (*84*) (fig. S14C). In mid-ventricular adult heart sections (Fig. 8, B to D), DPP4 was evident in a subset of adventitial *Pdgfra*^+^ fibroblasts of mid-to-small caliber coronary vessels but rarely around larger coronary arteries. Expression was also seen sparsely in *Pdgfra*^+^ CFs within the myocardial interstitium. Robust DPP4 expression in endocardium and epicardium was also detected (Fig. 8D), noting, however, that these populations are not efficiently captured in scRNA-seq pipelines (*9*, *10*). Our data are consistent with F-SH being equivalent to the *Pi16*^high^ cognate population in the mouse hearts localized principally to mid-to-low caliber coronary vascular adventitial niches, with some also within the interstitium. Quantification using flow cytometry showed that the DPP4^+^ population in healthy hearts represented 24% of live *Pdgfra*-eGFP^+^ ventricular CF, compared to 77% for SCA-1^+^, 51% for CD90^+^, and 96% for CD55^+^ cells, respectively (Fig. 8E and fig. S14, D and E). We have shown previously that selection for the PDGFRα^+^SCA-1^+^CD90^+^ CF subfraction enriches for MSC colony-forming units (*5*, *9*).

### CF subtype-specific responses to COVID-19

CV complications of SARS-CoV-2 (severe acute respiratory syndrome coronavirus 2) (COVID-19) infection are thought to be largely secondary to severe pulmonary disease, although a minor patient group shows myocarditis (*85*). Nonetheless, COVID-19 infection leads to gene expression perturbations in heart, including changes to immune, proliferative, and fibrotic pathways (*86*, *87*). In reported studies, however, CF substate changes were not delineated. Angiotensin-converting enzyme 2 (ACE2) is a key intermediate in the renin-angiotensin pathway and the main receptor protein for the COVID-19 virus, and *ACE2* has been recently reported to be expressed in human CF and pericytes (*19*). We asked if human CF states exhibit identifiable changes after severe COVID-19 infection. We used data from Delorey *et al.* (*88*), which compared cardiac snRNA-seq dataset from deceased individuals with severe COVID-19 infection to that of healthy donors, however, noting that donors varied with respect to age, ethnic background, disease, and treatment trajectories and likely comorbidities. We used Seurat label transfer to assign corresponding cluster labels in each dataset. Differential proportion testing using Propeller showed that there were no significant differences in CF cluster proportions with COVID-19 infection (fig. S15A). We did not see up-regulation of an MYO cluster as reported previously in COVID-19–infected lung (*82*). We evaluated DEGs between COVID-19–infected and healthy hearts, which revealed a total of 813 unique up-regulated and 880 down-regulated DEGs [adjusted *P* < 0.05; log_2_(fold change) > 0.5]. These were distributed across all CF subpopulations to different degrees (fig. S15B). Up-regulated DEGs were overrepresented for GO terms related to cell junction and focal adhesion assembly and cell differentiation themes (fig. S15C), consistent with previous reports (*86*–*88*). Notably, down-regulated genes were overrepresented for multiple terms relating to RNA processing and splicing (fig. S15D), a finding suggesting that RNA splicing is repressed in CFs during severe COVID-19 infection. Notably, this is also a tactic used by other viruses to favor replication and evade immune responses (*89*, *90*). Widespread dysregulation of transcript and protein isoform use has also been described in lungs of patients infected with COVID-19 correlated with disease severity (*89*, *90*).

### Human CF subtypes in hypertrophic heart disease

**Fig. 9.**
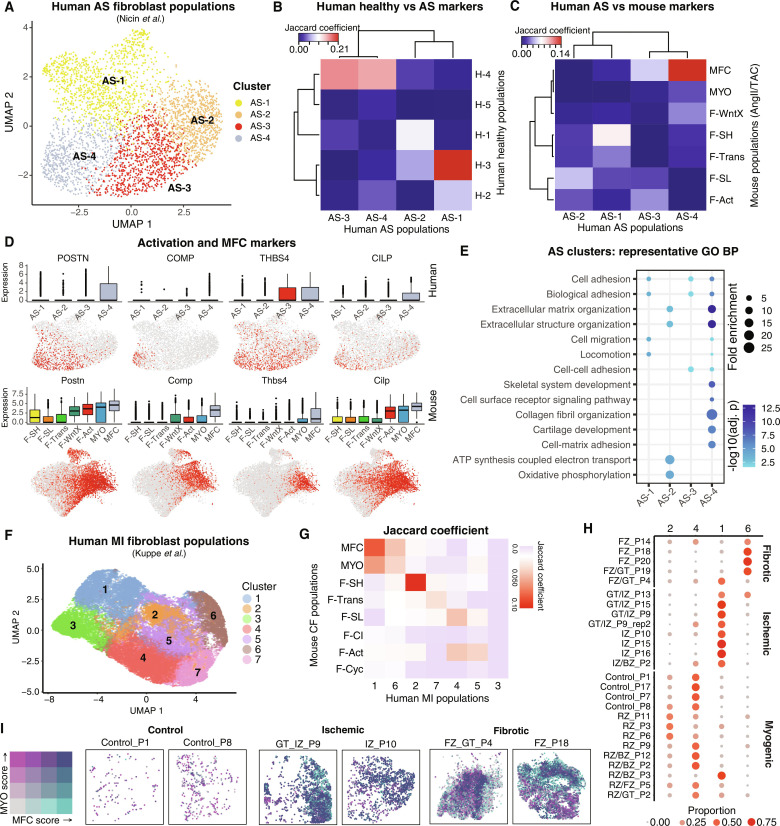
Analysis of CFs in human hearts from patients with AS and MI. (**A**) UMAP of human AS CFs with identified subclusters. (**B** and **C**) Jaccard coefficients for (B) the overlap of AS fibroblast population markers with markers from fibroblasts from healthy (H) hearts and (C) the overlap of human and mouse CF marker genes from AngII and TAC hearts [MAST testing; *P*_adj_ < 1 × 10^−05^; log_2_(fold change) > 0.5]. (**D**) Gene expression as visualized in box and UMAP plots comparing human AS fibroblasts (top) and mouse (bottom) AngII/TAC fibroblasts. (**E**) Representative GO BP terms overrepresented among AS fibroblast clusters. (**F**) UMAP of snRNA-seq–resolved human MI fibroblast populations colored by unbiased clusters. (**G**) Heatmap of Jaccard indices of marker genes for human MI fibroblast population clusters with mouse CF populations. (**H**) Dot plot displaying relative proportion of human MI fibroblast populations within each patient sample. Color and size of dots corresponds to relative proportion among the four clusters within each patient sample. (**I**) Spatial coordinates of Visium-resolved human MI samples, colored using bivariate color scheme corresponding to MFC and MYO scores. Only spots corresponding to the majority Fibroblast percentage are displayed. Patient samples are organized by control (left), ischemic (middle), and fibrotic (right).

To probe human CF states in chronic CV disease, we used a recent snRNA-seq dataset of left ventricle biopsies taken from patients with severe aortic valve stenosis (AS) with pressure overload–related cardiac hypertrophy (*22*). We identified four CF clusters (AS-1 to AS-4; Fig. 9A). Jaccard comparisons between human AS and healthy heart markers [table S10; MAST testing; *P*_adj_ < 1 × 10^−05^; log_2_(fold change) > 0.5] showed that cluster AS-1 was highly correlated with H-3 (homologous to mouse F-SH) (Fig. 9B). Both AS-3 and AS-4 correlated to healthy H-4 (similar to mouse F-Act). Comparisons were highly significant by Fisher’s exact tests (*P*_adj_ < 1 × 10^−75^; fig. S16A).

We next compared discriminating marker genes for human AS clusters to those from our mouse integration map filtering for AngII or TAC (pressure overload) disease conditions. Consistent with above, Jaccard coefficients revealed that AS-1 showed correspondence to mouse F-SH. Notably, AS-4 showed highly significant correspondence to MFC (*P*_adj_ < 1 × 10^−25^; Fig. 9C and fig. S16B) and up-regulated activation marker *POSTN*, as well as *COMP*, *THBS4*, and *CILP*, also up-regulated in mouse MFC (Fig. 9D). Additional MFC markers up-regulated included *FMOD* and *PRELP* (fig. S16C). MYO markers including *CTHRC1* and *ACTA2* were not up-regulated in AS-4 or any other clusters (fig. S16C), likely because of the advanced state of disease. In healthy human CFs, MFC markers were not up-regulated in any population (fig. S16D).

GO term analysis (Fig. 9E and fig. S16E) revealed that AS-4 was associated with BP terms related to ossification, including skeletal system development and cartilage development, as well as extracellular matrix organization and collagen fibril organization, highly similar to MFC in mouse (Fig. 6E), supporting functional conservation of MFC between murine and human diseased hearts.

### Spatial identification of MYO and MFC-like cells in human MI samples

Last, we used existing matching snRNA-seq and 10x Genomics Visium spatial transcriptomics datasets from human MI hearts (*21*) to assess if human MYO and MFC could be discriminated at different disease stages and their spatial relationship. After selecting previously assigned CF, clustering revealed seven distinct subclusters (H-1 to H-7) (Fig. 9, F and G, and fig. S17, A to E). Jaccard analysis revealed the strongest correlation between human MI subcluster H-2 and mouse F-SH (Fig. 9G), within which F-SH/*Pi16*^high^ cluster signature genes were enriched (fig. S17, B and C), confirming human-to-mouse conservation of this subtype as detailed above. Subcluster 4 showed the highest correlation with F-SL and F-Act, albeit at a lower Jaccard scores.

Subclusters H-1 and H-6 both exhibited strong correlations with MYO and MFC, and both showed overrepresentation of GO terms relating to extracellular matrix, collagen, cell adhesion, and vascular development (fig. S17, F and G). While a unique identity for H-1 and H-6 could not be clearly assigned from Jaccard scores, H-1 showed higher expression of *FN1*, *POSTN*, *COL1A1*, *COL3A1*, and *CTHRC1*, aligning with an MYO identity, whereas H-6 showed higher expression of *COMP*, *ITGBL1*, and *CFH*, aligning with an MFC identity (fig. S17, D, E, and H). Furthermore, the H-6/MFC gene signature showed partial overlap with all other CF clusters (except for MYO-like H-1), consistent with H-6/MFC adopting a less mature and deactivated state compared to MYO, as in mice (*46*). We next examined the degree of representation of H-1/MYO and H-6/MFC subclusters among MI patient clinical subgroups (*21*), finding that H-1/MYO was more enriched in ischemic zones of hearts with less advanced disease, whereas H-6/MFC was more enriched in fibrotic zones found uniquely associated with advanced fibrosis (Fig. 9I). This pattern aligns with the known lineage and spatiotemporal relationships between MYO and MFC in mice, including their respective early and late peaks following MI (Fig. 1, B, C, and F) (*46*). In contrast, other subclusters including H-2/F-SH and H-4/F-SL were associated with preserved myogenic regions consistent with being resting populations.

We next exploited these transcriptomics data to examine the spatial relationship between H-1 (MYO-like) and H-6 (MFC-like) in MI hearts (*21*). In Visium “spots” (average four nuclei per spot) (*21*), we generated MYO and MFC scores as a weighted sum of the expression of top H-1 and H-6 markers specific to fibroblasts, respectively (fig. S17H and table S10) (see Materials and Methods). First, we confirmed that MYO and MFC scores were high in spots labeled Fibroblast (fig. S17I) (*21*). We next documented distributions of spots with high MYO and MFC scores among clinical samples and heart anatomical regions (figs. S18 and S19). As expected, highly scoring spots were sparse in controls. Among diseased hearts, highest ranked scores for MYO and MFC were found mostly in ischemic and fibrotic zones, respectively (fig. S18, A and B). Notably, there was considerable variability in the degree of spatial overlap between spots with high MYO and MFC scores (fig. S18C). For example, in samples with the greatest density of high-scoring spots, mostly in fibrotic and ischemic zones as expected (fig. S19), there were regions of high spatial concordance as well as neighboring clusters of MYO-only or MFC-only spots (Fig. 9I). In summary, F-SH, F-SL, MYO-like, and MFC-like cells could be distinguished in human MI hearts (*21*). There was focal clustering of spots with high MYO and MFC scores in fibrotic and ischemic zones with high although variable spatial overlap, likely reflecting both the heterogeneity in disease timing and mechanism in samples analyzed, and conservation of a dominant lineage relationship between MYO and MFC in human as in mouse hearts.

## DISCUSSION

Here, we report integration mapping of CFs states in homeostasis and disease in mouse and human ventricles. Our data extend initial integration mapping on cardiac cell types (*21*, *47*, *82*, *91*, *92*), revealing insights into ventricular CF identities, conservation, and fates and the underlying biology of CV disease pathogenesis.

### Integration of early resting CF subtypes

We confirmed the presence of resting F-SH cells (*Pdgfra*^+^;*Ly6a*/*Sca1*^high^) (Table 1) in different mouse disease models. F-SH cells are enriched in stem/progenitor cell markers (*5*, *6*, *8*), reside in a hypoxic niche (*8*), and potentially show preferential proliferation after cardiac injury (*5*, *6*, *8*, *32*). In vivo lineage tracing suggests considerable flux between SCA1^high^ and SCA1^low^ CF populations, even in healthy hearts, over a time frame of weeks (*6*), supporting a dynamic equilibrium between CF cell states. We show that F-SH cells are homologous to the *Pi16*^high^ cluster found conserved across different mouse and human organs (*82*). *Dpp4* expression is a marker of stem cell population in other tissues and was selected as a highly discriminative marker for F-SH. Immunostaining for DPP4 demonstrated that most F-SH cells reside within the adventitia of mid-to-small caliber coronary arteries but can also be found within the interstitium. The vascular adventitia contains multiple cell types including MSC-like cells dedicated to vessel homeostasis and adaption, and these display immune and fibrotic sentinel responses to diverse stimuli including hypertension and hypoxia (*83*, *93*–*99*).

F-Act is closely related to F-SH and present in healthy hearts and all after injury stages. While largely immature and retaining stem cell characteristics, a subset of non-dividing F-Act cells express activation markers in a graded distribution across UMAP space, which in injured hearts includes MYO markers *Acta2* and *Cthrc1*. These F-Act cells, while distinct from MYO, appear to have undergone commitment to myofibrogenesis. We propose that F-Act represents an early-tier CF activation state reflective of a reversible sentinel function, which creates an “alert” condition (*100*), similar to other stem cell populations (*101*), tipping toward proliferation and definitive fibrogenesis and potentially other fates after injury.

### Early injury–specific CF populations

After MI, early injury–induced populations, IR, F-CI, and F-Cyc, arise sequentially. TF network analysis showed that these states represent a closely related triad, separate from homeostatic populations, results that can now be explored further using single-cell chromatin accessibility assays. IR was first evident at MI-day 1 (*10*) and expressed monocyte/macrophage proinflammatory cytokines and inflammatory pathway genes, suggesting a role in innate immunity (*15*, *100*, *102*). Along with the other early CF populations, IR expressed higher levels of antioxidant and other prosurvival genes, likely adaptations to the hostile milieu after MI. Using MT1 and MT2 as markers, Forte *et al.* (*10*) reported that IR cells were abundant in both injury and remote zones of infarcted ventricles, pointing to IR as a pan-ventricle IR. However, this would seem at odds with expectations that injury-induced proinflammatory cells cluster at the infarct and border zones in response to damage-associated molecular patterns (*102*). We confirmed that *Mt1* and *Mt2* are among the top DEGs for IR; however, both genes are expressed in other CF populations, so the spatial distribution of IR requires confirmation with additional markers and/or spatial RNA-seq. Our integrated data have allowed us to refine our understanding of IR in several respects. First, we confirmed that IR peaks at MI-day 1, as reported previously (*10*); however, our clustering shows that IR cells are virtually all resolved by MI-day 3. CFs previously assigned as IR at MI-day 3 by Forte *et al.* correspond in our integration study mostly to proliferative phase populations F-CI and F-Cyc, most of which appear committed to an MYO fate. However, they remain distinct from MYO, which represents a more advanced maturational state. IR cells have not yet initiated myofibrogenesis yet are primed for proliferation. IR may transition to MYO via F-CI and F-Cyc; however, the precise fate of IR remains unknown and cannot be inferred from available static single-cell data. An alternative fate for IR is that they undergo cell death during inflammation resolution, noting that >60% of infarct zone cells at MI-day 1 are positive for TUNEL (terminal deoxynuleotidyl transferase–mediated deoxyuridine triphosphate nick end labeling) assay (*8*). Our integration data have allowed us to refine the CF proliferative window (*46*, *103*), with commitment to division occurring in IR at MI-day 1 and F-CI and F-Cyc evident until MI-day 7.

F-CI cells are nonproliferating CFs that appear first at MI-day 3, coincident with cycling fibroblasts (F-Cyc). F-CI and F-Cyc are closely related populations (*9*, *44*), and trajectory analysis suggests that F-CI includes both preproliferative CFs and those having just completed the cell cycle. Thus, F-CI can be thought of as an MYO progenitor pool. At MI-day 7, after MYO has peaked, F-CI was considerably depleted. Trajectory analysis predicts that MYOs arise mostly from F-CI, although direct routes from other CF subtypes, including F-Cyc, may occur.

### MYO and MFC

Definitive MYOs appear sparsely at MI-day 3 and peak at MI-day 5 before being supplanted by MFCs. We confirm here that MYO is composed of at least two substates—MYO-1 and MYO-2 (*9*). Although both are MYO in character, MYO-1 shows antifibrotic features, including higher expression of *Ccn5/Wisp2*, which can inhibit TGFβ signaling and reverse established fibrosis (*104*), soluble WNT antagonist genes *Sfrp2* and *Sfrp1*, and lower expression of profibrotic transcriptional drivers, suggesting distinct functional properties. Unbiased classification revealed that MYO-1 and MYO-2 are present as soon as MYOs appear in substantial numbers at MI-day 5 and persist in similar proportions after the MYO peak at MI-day 7. A notable additional finding was that MYO-1 and MYO-2 signatures also occur within MFC, at a time when MYOs are completely resolved. Opposing gradients of MYO-1 versus MYO-2 markers across UMAP space hint at similar gradients in vivo. These findings challenge our current view of MYO and MFCs (*46*). One possibility is that MYO-1 and MYO-2 represent early and late forms of MYOs, respectively, noting that MYOs have been classified previously on continuous scales of morphologies and contractile states (*105*, *106*). Alternatives are that they indicate MYO and MFC polarization and the self-limiting nature of fibrosis (*46*, *102*, *107*, *108*), a finding with possible therapeutic implications (*109*), or that they exist within distinct niches.

### Cross-integration of different disease states

Our integrated MI time course allowed us to accurately profile other CV disease states. At day 5 of IRI, CF populations resembled those of MI-days 5 to 7, although IRI showed a substantially higher proportion of MYO. This may result from increased infiltration of inflammatory cells after reperfusion, leading to amplification of myofibrogenesis.

In advanced stages of pressure overload cardiomyopathy (AngII and TAC), associated with “reactive fibrosis” (*4*), we identified an increase in F-Act, abundant MFC, and relative absence of MYO, features in common with late stages of MI. We established the AngII model and demonstrated *Pdgfra*-lineage^+^α-SMA^+^ MYOs colocalizing with focal perivascular and interstitial collagen deposition at day 7; however, α-SMA^high^ MYOs were resolved by day 14. This demonstrates that AngII pressure overload involves transient myofibrogenesis in both perivascular and adventitial compartments. We predict that, in the AngII model, MFCs derive largely from MYO, as in MI (*46*). Consistent with our findings, a recent mouse scRNA-seq study identified a CF population expressing *Postn*, *Comp*, and *Thbp4*, increasing after TAC to day 28 (*47*). This was proposed to be MFC after integration analysis.

There were no overt signs of fibrosis or changes in CF population proportions in a two-hit C57Bl/6J model of HFpEF, a clinically relevant form of HF. Nonetheless, by 15 weeks, a profibrotic gene expression signature was emerging across all CF subtypes. In the original report of the HFpEF model performed in C57Bl/6N strain (divergent from C57Bl/6J), modest focal collagen staining was seen at 5 weeks of treatment, although fibrosis appeared to be purely a function of hypertension, being induced by l-NAME alone (*78*). In a more recent single-cell study of HFpEF hearts also performed in C57Bl/6N mice, a modest increase in fibroblasts was detected at 7 weeks after treatment, with ECM and inflammatory pathways being induced (*110*). Collectively, these studies suggest that cardiometabolic stress, not fibrosis, is the principal driver of HFpEF, consistent with preclinical studies (*79*, *111*, *112*) and molecular profiles in patients with HFpEF (*77*). However, the extent of fibrosis in HFpEF is likely modulated by the degree of hypertension and genetic background, as demonstrated here.

### Adaptation of F-Act and MFC to distinct injury environments

Our integrated CF map showed high concordance of MFC and F-Act DEG signatures in ischemic versus pressure overload injury models, reinforcing the unique identity and stability of MFC and F-Act. This aligns with a model of cardiac fibrosis whereby diverse injurious stimuli converge on a common fibrotic cascade. However, both MFC and F-Act show pronounced differential transcript isoform use between ischemic and hypertensive models, reflecting adaptation of CF subtypes to distinct injury environments, a level of network control thus far unexplored. Intron retention has been recently identified as a mode of transcriptional regulation associated with cardiac infarcts (*113*).

### Conservation of CF states

Five CF subpopulations were detected after in silico processing of data derived from left ventricles of healthy human donor hearts (*19*), aligning with other large-scale studies (*21*, *82*, *91*). Homology of the healthy human population H-3 with F-SH in mice was strongly supported, consistent with previous findings of the conservation of *Pi16*^high^ cluster CF across species and organs (*82*). A human F-Act–like state (H-4) was also suggested in healthy and MI hearts.

Single-cell studies on human heart failure samples have identified various activated CF states including those proposed to represent MYOs (*20*, *21*, *24*, *92*), although these have not been studied in detail. We concentrated first on human AS-induced hypertrophic cardiomyopathy (*22*), allowing us to compare with our integrated mouse pressure overload data. We detected four main populations. AS-1 showed strong correlation to the universal healthy human H-3 population and F-SH in mice, whereas AS-3 showed some similarity to healthy human H-4 and mouse F-Act. Our analysis also compellingly confirmed human AS-4 as homologous to mouse MFC, found at late stages of mouse AngII/TAC and MI models. These findings strongly support conservation of MFC in pressure overload cardiomyopathy between mice and humans. Our data also support the presence of F-Act and MFC in human MI (*21*), along with MYO, F-SH, and F-SL.

In a snRNA-seq study of 26 failing human hearts (ventricular biopsies), a population of activated fibroblasts expressing *POSTN*, *COL22A1*, and *THBS4*, consistent with MFC-like cells, were present in failing but not control donor hearts (*20*). No MYOs were specifically detected. Furthermore, given that hearts used in this study were in advanced (pretransplantation) stages of failure, the MFC-like cells were only abundant in 2 of 26 patients, one with dilated and the other hypertrophic cardiomyopathy. However, a follow-up study on a wider set of controls and patients with available bulk RNA-seq data confirmed the detection of MFC-like cells in only limited advanced heart failure samples but also suggested that MFC will be more prevalent at earlier disease stages (*20*). The presence or absence of MFC-like cells was not correlated with left ventricular ejection fraction, mass, or wall thickness nor history of diabetes, atrial fibrillation, or hypertension. Collectively with our own findings, these data establish that MFCs are an integral component of CF trajectories in diverse forms of cardiomyopathy in mice and humans. Deeper analysis of fibroblast activation and resolution in human CV disease states is warranted.

Analysis of Visium spatial transcriptomics data in ventricular biopsies from patients with MI (*21*) showed strong colocalization of spots scoring highly for MYO and MFC signatures, adjacent to regions enriched in either MYO-high or MFC-high spots. This is consistent with the known lineage relationship between MYO and MFC in mice (*46*) and sheds light on how MYO resolution plays out, in 3D, in the human MI setting in which ischemic and fibrotic zones are heterodisperse.

Figure S20 shows a summary diagram representing our findings on CF dynamics under different injury conditions to date and likely mouse-human conservation. However, projected timelines for CF dynamics during truly chronic pressure overload responses in humans and the conservation status of some human populations require additional data.

Integrated single-cell data maps pave the way for generation of multispecies, multilineage, and multidimensional tissue atlases that will drive forward better understanding of human and animal biology and treatment of disease. CV diseases remain the highest cause of death and disability worldwide, and development of antifibrosis therapies has been challenging. Progress may be accelerated by deeper understanding of CF agency and function and path to fibrosis resolution. Our data on diverse CV disease models should inform current efforts to reconstruct 3D tissue context through spatial transcriptomics and will add a deeper perspective to development of novel therapeutics.

### Limitations of the study

Our study was specific to the cardiac ventricles—atrial, valvular, vascular, and other CFs present in whole hearts may have distinct signatures and trajectories (*19*). In different studies, there may be technical limitations in recovery of certain cell types or biases due to differences in experimental design including cell enrichment protocols, ancestry, gender, baseline physiology, and extent of disease. Bioinformatic processes, including QC and definition of cell types and states, are operator and software-dependent and involve subjective decisions, as described previously (*24*). As such, inferences about cell annotation, trajectories, spatial context, and function will require validation using multiple approaches including spatial transcriptomics. Our chosen population nomenclatures principally reference those of foundation studies (see Table 1) (*9*, *10*). More systematic nomenclature will be needed in the future. Integration of other cell types, including cardiomyocytes, endothelial, perivascular, and immune cells, will be necessary to further progress meaningful 3D tissues maps.

## MATERIALS AND METHODS

### Datasets

Data from *Pdgfra*-eGFP^+^/CD31^−^ cells from sham- or MI-days 3 and 7 mice are available on ArrayExpress under ID E-MTAB-7376 (*9*). Cardiac interstitial cell data from healthy hearts and MI-days 1 to 28 can be found on ArrayExpress under ID E-MTAB-7895 (*10*). *Pdgfra*-TdTom^+^/CD31^−^/CD45^−^ cell data from sham- or MI-day 3 mice are available on ArrayExpress under ID E-MTAB-9583 (*8*). *Col1a1*-GFP^+^ cell data from sham- or MI-days 7, 14, and 30 mice were obtained from the Gene Expression Omnibus (GEO) under accession number GSE132146 (*28*). scRNA-seq of cells from sham or TAC mice were obtained from GEO under accession number GSE155882 (*51*). Data from scRNA-seq of healthy or AngII mice are available on ArrayExpress under ID E-MTAB-8810 (*63*). Data from sham or ischemia-reperfusion hearts are available on ArrayExpress under ID E-MTAB-10035 (*62*). The sc/snRNA-seq datasets for HFpEF created in this study have been deposited in ArrayExpress (accession number E-MTAB-13362). For the healthy human heart analysis, processed counts were downloaded from GEO (accession number GSE156707) (*80*). For analysis of hypertrophic human hearts, the data are available on ArrayExpress under access E-MTAB-11268 (*22*). For the human COVID-19 analysis, a combined file of cardiac cells from individuals infected or uninfected with COVID-19 was downloaded from https://singlecell.broadinstitute.org/single_cell/study/SCP1216 (heart_meta_study_all_cells_used_for_DE.h5ad) (*88*). Human MI snRNA-seq and Visium data were downloaded from the publication’s data availability URL (*21*). Processed Seurat objects for mouse integration analyses, plus healthy and AS human fibroblasts, have been deposited on Synapse (https://synapse.org/) under project ID syn52429757.

### Alignment and quantification

All mouse data were processed from Fastq files to count matrices using the CellRanger 6.0.2 count program against the mouse mm10 reference genome (2020-A-2.0.0) downloaded from the 10x Genomics website (https://support.10xgenomics.com/single-cell-gene-expression/software/pipelines/latest/what-is-cell-ranger). Individual datasets were aggregated by using the CellRanger aggr program with the normalize parameter set to “none.”

### QC filtering and clustering

Clustering analyses were performed using the Seurat R package (v 4.0.3). Jupyter notebooks containing the analysis pipeline for each of the datasets is available at https://github.com/VCCRI/FibroblastIntegration. Briefly, the distributions of unique molecular IDs and gene counts were visualized, with outliers filtered. Cells were filtered for percent of mitochondrial content. Initial clustering was performed following a basic Seurat pipeline, including detection of top variable genes, scaling, dimensionality reduction with principal components analysis (PCA), construction of nearest neighbor graphs using the Seurat FindNeighbors program, and clustering with FindClusters. Doublets were estimated by running Scrublet (*114*) on individual single-cell datasets and visualized on UMAP plots prior to filtering to retain singlets. Following doublet filtering, the clustering steps, from detection of variable genes, was rerun. Clusters were inspected for stressed or damaged cells, with clusters representing likely damaged cells removed. Fibroblasts were identified among the returned clusters by the expression of fibroblast marker genes (including *Pdgfra*, *Col1a1*, and *Tcf21*) and filtered for further analysis. The fibroblast subclusters for the MI datasets were manually annotated in accordance with previous fibroblast subpopulation designations according to marker gene expression (*9*, *10*). For our previous datasets [*Pdgfra*-eGFP^+^/CD31^−^ cells (*9*) and *Pdgfra*-TdTom^+^/CD31^−^/CD45^−^ cells (*8*)], population IDs were maintained in accordance with the original publications.

### Integration of mouse cardiac datasets

Integration of the MI and cross-disease mouse datasets was performed using the cFIT R package (v 0.0.0.9) (*25*). For the MI integration, we performed integration on the following six sets of conditions and cell isolation strategies:

1) Uninjured versus MI hearts at MI-days 1, 3, 5, 7, 14, and 28 from interstitial cells of cardiac ventricles (*10*).

2) Sham- versus MI-day 3 on *Pdgfra*-eGFP^+^/CD31^−^ cells from cardiac left ventricles (*9*).

3) Sham- versus MI-day 7 on *Pdgfra*-eGFP^+^/CD31^−^ cells from cardiac left ventricles (*9*).

4) Sham- versus MI-day 3 on *Pdgfra*-TdTom^+^/CD31^−^/CD45^−^ cells from cardiac left ventricles (*8*).

5) MI-day 14 from *Col1a1*-GFP^+^ cells from cardiac ventricles (*28*).

6) MI-day 30 from *Col1a1*-GFP^+^ cells from cardiac ventricles (*28*).

For the *Col1a1*-GFP^+^ data from Ruiz-Villalba *et al.* (*28*), we noted that batch effects appeared to exist between each condition, in line with the approach of the original paper, which used the Seurat canonical correlation analysis (CCA) integration method between the conditions prior to analysis (*28*). Therefore, we selected the two later MI time points (MI-days 14 and 30), where enriched CFs were not already available and incorporated these as separate datasets for integration.

For the cross-disease integration, we used the above MI datasets, in addition to the following:

1) Total cardiac cells from the cardiac ventricles of AngII- or saline-treated mice (*63*).

2) Cardiac interstitial cells from cardiac ventricles of sham- or TAC-day 62 operated mice (*51*).

3) Enriched CFs (*Wt1*-tdTom^+^/CD31^−^/CD45^−^) from the hearts of sham or ischemia-reperfusion–day 5 operated mice (*62*).

The integration for both MI and cross-disease datasets was performed on 4000 variable genes, out of a union of genes expressed across the datasets, identified with the select genes function in cFIT (*25*). For the MI integration, we used the CFITIntegrate function with the *r* value, representing expected biological heterogeneity, set to 15. For integration of the larger cross-disease dataset, we used the CFITIntegrate_sketched function with the same *r* value of 15 and subsample.prop set to 0.5.

For comparisons with alternative integration methods, we integrated the MI datasets using the Seurat (*26*) CCA approach, Harmony (v 0.1.0) (*29*), and RISC (v 1.0) (*30*). For consistency, we used 4000 variable genes, as above, for each of the comparison methods. Seurat integration was performed as follows. Functions NormalizeData and FindVariableFeatures were run on each condition with nfeatures set to 4000 for FindVariableFeatures. SelectIntegrationFeatures was run with nfeatures set to 4000. FindIntegrationAnchors and IntegrateData followed by ScaleData and RunPCA were all run with default parameters. UMAP coordinates were generated using the RunMAP function, with n.neighbors set to 200, min.dist = 0.5, and dims (PCA) set to 50.

For the Harmony comparison, variable genes were identified as above, followed by scaling and PCA dimensionality reduction using the Seurat functions. The RunHarmony function was run using the top 50 principal components (PCs), with UMAP coordinates generated using the Seurat RunUMAP function as above. For RISC, we followed the pipeline as outlined in the software vignette, identifying 4000 variable genes per dataset and using the union of all variable genes for integration. We used the scMultiIntegrate function with eigens = 15, npc = 50, and align = “OLS.” UMAP coordinates were generated using the scUMAP function with npc = 50.

For nearest neighbor comparisons between the methods, we used the Seurat FindNeighbors function to identify the top 200 nearest neighbors for each cell, based on the reduced dimensions (e.g., PCA) used for calculating UMAP. For each cluster, we calculated the average percentage of cluster identities that nearest neighbor cells belonged to. These percentages were plotted as heatmaps using the ComplexHeatmap R package (*115*).

### Analysis of human datasets

For the healthy heart data (*80*), processed counts were downloaded from GEO. For the AS hearts (*22*), Fastq files were downloaded and processed to counts using CellRanger count and aggr as above but mapping to the GRCh38-2020-A reference. For clustering of the human datasets [both healthy hearts (*80*) and AS hearts (*22*)], the individuals were integrated prior to clustering using the Seurat CCA integration pipeline (see Jupyter notebooks at https://github.com/VCCRI/FibroblastIntegration). Briefly, following doublet removal with Scrublet, data were log-normalized, and 2000 variable genes were identified for each sample. The SelectIntegrationFeatures (with nFeatures = 2000), FindIntegrationAnchors, and IntegrateData functions were run, followed by data scaling, dimensionality reduction with PCA, and clustering performed on the top 50 PCs using the FindNeighbors and FindClusters functions. Fibroblast clusters were identified according to the expression of fibroblast markers and absence of markers of other cardiac cell lineages (including cardiomyocytes, endothelial cells, and leukocytes) and selected for further analysis. PCA was then rerun on the integrated fibroblasts, with clustering and calculation of UMAP coordinates performed on the top 40 PCs.

For the analysis of CFs from the COVID-19 study, we used the CF clusters defined in the healthy human heart as a reference point for label transfer analysis. We used a combined dataset of cardiac cells from two sets of healthy individuals and two datasets of individuals infected with severe COVID-19 (*88*). As major cell types had been assigned, we first selected the cells annotated as fibroblasts. We then processed the datasets as follows. For each dataset, we retained individuals with greater than 100 fibroblast cells. Similar to our analysis of human data above, we applied the Seurat integration pipeline to remove batch effects between the individuals by running the Select IntegrationFeatures (with nfeatures = 2000) and FindIntegrationAnchors functions on log-normalized data. The data were then scaled prior to PCA and clustering and UMAP run on the top 40 PCs. We filtered out clusters that appeared to be nonfibroblast cells (based on markers of cardiomyocyte, immune, endothelial, glial, and mural cells) and thus further enriched for fibroblasts. To use a consistent nomenclature and determine if the CF clusters we had characterized in healthy hearts were altered following severe COVID-19 infection, we used Seurat label transfer analysis to impose the cell identities from our analysis of the Sim *et al.* data (*80*) on the healthy and COVID-19 datasets. We processed each dataset individually, using the FindTransferAnchors and TransferData functions with dims = 1:40.

### RF predictions

For prediction of profibrotic versus antifibrotic signatures in MYOs and MFCs and prediction of CF states in the cross-disease map, we trained RF classifiers on the cFIT-integrated data using the Ranger R package (v 0.13.1) (*116*). In both cases, RF classifiers were trained using 500 trees and subsequently applied by taking the highest-scoring cell type prediction. Before application to the relevant dataset, the accuracy of the classifiers were evaluated through 10-fold cross-validation experiments, with prediction accuracy measured using multiple metrics including sensitivity, specificity, and balanced accuracy.

For the MYO-1 versus MYO-2 predictions, we could distinguish the two populations with a balanced accuracy of 0.93 according to the 10-fold cross-validation simulations.

### Trajectory analyses

Trajectory analysis of the integrated MI datasets was performed with PAGA (*48*) through the Scanpy (v 1.9.5) Python package (*117*). For the three comparisons (healthy, MI-days 1 to 5, and MI-days 7 to 14), the subsets were used to calculate top 10 nearest neighbors based on the 15 dimensions returned by cFIT. We used the SaveH5Seurat and Convert functions in Seurat to generate h5ad files for Scanpy. The Scanpy function sp.pl.paga (with layout = “fr” and threshold = 0.03) was used to generate the PAGA graph, and sp.pl.draw_graph was used to generate the force-directed layout graph.

Trajectory analysis was also performed using Monocle 3 (*49*). Monocle was run on UMAP coordinates from indicated subsets of conditions: healthy and MI-days 1 and 3, 3 and 5, 5 and 7, 7 and 14, and 14 and 28/30. For each data subset, the trajectory graph was calculated with the learn_graph function with parameters rank.k = 50, maxiter = 15, and minimal_branch_len = 10 and trajectories plotted using the plot_cells function.

### Differential expression testing

Unless otherwise stated, DEGs were determined using the Seurat FindMarkers program with MAST testing (v.1.18.0) (*50*). A Bonferroni-adjusted *P* value of 1 × 10^−05^ and a log_2_(fold change) difference of 0.5 were used to determine significance.

For the analysis of HFpEF samples, pseudo-bulk replicates of each cell population were generated according to biological replicates (*n* = 2 for HFpEF and normal chow). For each cell type, genes were evaluated if they were expressed in ≥10% of cells in either condition. DESeq2 (v 1.32.0) (*118*) was applied for differential expression testing, and the fdrtool package (v 1.2.16) (*119*) was used for *P* value correction. Genes returning adjusted *P* < 0.05 and an absolute log_2_(fold change) difference of 0.5 were considered significant.

For the COVID-19 analysis of human samples, a pseudo-bulk approach was taken. Within a cell population, each sample (representing an individual) was used to generate pseudo-bulk profiles for the individuals infected with COVID-19 and healthy individuals. As these samples are composed of five separate studies (representing three healthy and two COVID-19 datasets), we used the RUVSeq (v 1.26.0) R package (*120*) to normalize out technical variation as part of the differential expression analysis. To obtain control genes, we first applied DESeq2 (*118*) to calculate differential expression between the individuals infected with COVID-19 and healthy individuals and retained genes that were outside the top 5000 differentially expressed. The control genes and pseudo-bulk count matrix were used as input to the RUVg function with the *k* value (representing the estimated number of sources of technical variation) set to 3. The *W* matrix returned by RUVg was used as input into DESeq2 for an updated DE analysis. Genes returning adjusted *P* < 0.05 and an absolute log_2_(fold change) difference of 0.5 were considered significant.

### Differential transcript usage

DTU was determined using the Sierra R package (v 0.99.27) (*44*). Peak calling was run on the CellRanger BAM files utilizing the same reference file as for alignment. Peaks were merged within each study and read counting performed using the CountPeaks method. DTU testing was performed on each study by comparing the population of interest (MFC or F-Act) to the F-SH population from each respective uninjured control. DTU genes were evaluated using the Sierra DUTest function, requiring peaks to be expressed in a minimum of 10% of cells (exp.thresh = 0.1) and feature.type set to include “UTR3” and “exon,” An adjusted *P* value of 0.05 and a log_2_(fold change) difference of 0.5 was used to determine significant DU peaks. Analysis of differences in 3′UTR length was determined using the DetectUTRLengthShift and PlotUTRLengthShift functions, with default parameters.

### GO testing

GO term overrepresentation testing was performed using the ViSEAGO (*121*) R package. The BiomaRt package (*122*) was used to convert gene symbols to Uniprot/Swissprot IDs, and GO annotations for the genes were performed using the ViSEAGO Uniprot2GO and annotate functions. Overrepresentation testing was performed using the TopGO (*123*) runTest function, with algorithm = “classic” and statistic = “fisher” and the set of expressed genes used as background. The returned *P* values were adjusted for multiple testing using Benjamini-Hochberg correction, with GO terms considered significant if they obtained an adjusted *P* value of <0.05.

### TF network analysis

We performed analysis of TFs predicted to regulate different fibroblast populations using the decoupleR package (v 2.2.2) (*53*). DoRothEA (v 1.8.0) (*124*) TF regulons were retrieved using the get_dorothea function with levels “A,” “B,” and “C.” The log_2_-normalized counts were input to the run_wmean function with times = 100 and minsize = 5. The average weighted mean in each population/MI time point tested was then used to rank the TF according to the cell type and condition of interest. Heatmaps were generated using the pheatmap (v 1.0.12) R package (*125*).

### Differential proportion testing

Differences in cell type proportions were evaluated in the HFpEF and COVID-19 experiments using the propeller function from the Speckle R package (v 0.0.2) (*126*). In both experiments, sample was set to biological replicates and group was set to experimental condition (HFpEF versus normal chow and time point or COVID-19 versus uninfected). A false discovery rate of 0.05 was used to determine significance.

### Ethics statement

Mice were bred and housed in the BioCORE facility of the Victor Chang Cardiac Institute. All experimental procedures were approved by the Garvan Institute/St. Vincent’s Hospital Animal Experimentation Ethics Committee (nos. 19/07 and 19/14) and performed in strict accordance with the National Health and Medical Research Council of Australia Guidelines on Animal Experimentation. Mice were maintained on a 12-hour light/dark cycle from 6 a.m. to 6 p.m. and had unrestricted access to food and water.

### Mouse strains

1) Wild-type [inbred C57BL/6J] (the Jackson Laboratory; stock no. 000664).

2) Pdgfra^MCM/+^ [Pdgfra^tm1.1(cre/Esr1∗)Nshk^] (RIKEN, Japan; MGI catalog no. 5475226).

3) Pdgfra^H2BEGFP/+^ [B6.129S4 Pdgfratm11(EGFP)Sor/J] (the Jackson Laboratory; stock no. 007669).

4) Rosa26-tdTomato [B6.Cg-Gt(ROSA)26Sortm9(CAG-tdTomato)Hze/J] (the Jackson Laboratory; stock no. 007909).

### Lineage tracing

For irreversible labeling and fate mapping of PDGFRα^+^ cells, *Pdgfra*^MCM/+^ males were mated with *Rosa*^Tom/Tom^ females on the C57BL/6J background. Reporter activation in 8- to 12-week-old male *Pdgfra*^MCM/+^; *Rosa*^Tom/+^ mice was induced by three intraperitoneal injections of tamoxifen (Sigma-Aldrich, A9525) (100 mg/kg body weight) on consecutive days.

### MI and AngII heart failure models

MI was performed as described previously (*8*). Hypertension-induced cardiac hypertrophy and fibrosis were achieved by subcutaneous implantation of osmotic pumps (Alzet, model 2002) loaded with AngII (Sigma-Aldrich, United States; no. T5648) (1000 ng/kg per min) or vehicle (saline). Osmotic pumps were implanted 1 week after the tamoxifen treatment, and hearts were subjected to cells or tissue analysis at indicated time points after implantation.

### HFpEF Model

The mouse HFpEF model was induced in the C57BL/6J background by HFD + l-NAME (*N*ω-nitro-l-arginine methyl ester hydrochloride) (Sigma-Aldrich, no. N5751-25G) feeding for 15 weeks as described previously (*78*). Ten- to 12-week-old male mice were fed with a normal chow diet or HFD (Specialty Feeds, Australia; no. SF13-092). l-NAME was dissolved in drinking water (0.5 g/liter, pH = 7.4). The development of HFpEF was monitored via blood pressure taken via tail cuff and by echocardiography.

### Analysis of HFpEF sc/snRNA-seq data

The Fastq files were processed to count matrices using the CellRanger 6.0.2 count program against the mm10 reference used above. As the data contain both cells and nuclei, we ran CellRanger count once to count exons only and a second time to include introns by setting --include-introns. Individual datasets for exons only or exons plus introns were aggregated using the CellRanger aggr program with the normalize parameter set to “none.” Doublets were predicted and removed using Scrublet on the exon counts, with a doublet score threshold of 0.25 used to classify cells as doublets.

As a combination of single cells and nuclei are a confounding factor for clustering, we first identified and integrated the cells and nuclei as follows. A previous work (*127*) has shown that ribosomal genes and long noncoding RNAs (lncRNAs) can discriminate between cells and nuclei, respectively. We therefore defined a “ribosomal score,” based on all ribosomal genes using the Seurat AddModuleScore function. Similarly, all lncRNAs as marked in the CellRanger mm10-2020-A GTF file were used to generate an lncRNA module score. The product of these two scores was taken, and the dataset was split according to the ribosomal × lncRNA score, with a score >−0.5 representing putative nuclei and <−0.5 representing putative cells.

After the datasets were split into putative cells and putative nuclei, these two datasets were integrated using the Seurat integration pipeline. For the purposes of integration, the counts for the nuclei were updated to include introns. Each dataset was log-normalized, and the top 3000 variable genes were identified with the FindVariableFeatures function. Integration features were selected with the SelectIntegrationFeatures function, with features set to 3000. The FindIntegrationAnchors and IntegrateData functions were then run. The data were then scaled and reduced with PCA, and the FindNeighbors function was run on the top 40 PCs. FindClusters was run using a resolution of 1.2. RunUMAP was run on the top 40 PCs. A total of 34 clusters were returned an annotated based on marker gene expression. Of these, one was identified as contaminating red blood cells and removed, and a second defined by up-regulation of mitochondrial genes was filtered out.

### TMT discovery proteomics sample preparation

Tandem mass tagged (TMT) discovery proteomics was carried out by specialist staff at Sydney Mass Spectrometry, The Charles Perkins Centre, University of Sydney, Australia. Heart tissue was powdered under liquid nitrogen, before 20 mg was mixed with 400 ml of sodium deoxycholate buffer and heated to 95°C for 10 min at 1000 rpm, in a thermomixer. The samples were processed in a bead beater for 3 min at 50 Hz. A solution of 10 mM tris(2-carboxyethyl)phosphine and 40 mM chloroacetic acid was added to each sample, which was then returned to the thermomixer to reduce and alkylate proteins at 95°C for 10 min at 1000 rpm. Sample cleanup was carried out using chloroform/methanol precipitation and resolubilized in 6 M urea/2 M thiourea. Following Qubit quantitation, 100 mg was taken from each sample and made up to 50 ml with urea/thiourea. The proteins were then digested by the addition of 2 mg of porcine trypsin and incubated at 37°C in a thermomixer for 16 hours at 1000 rpm.

### TMT labeling, mid-pH fractionation, and data collection

The peptides were purified using Oasis hydrophilic-hydrophobic–balanced (HLB) with short cartridges (Waters Corp., Milford, MA, United States) and resuspended in a total volume of 30 μl with 100 mM Hepes buffer (pH 8.5). For each sample, 7-μg total peptides were labeled with TMT pro 16plex Isobaric labeling reagent (Thermo Fisher Scientific, MA, United States), following the manufacturer’s instructions. The TMT-labeled peptides were purified using an HLB column and fractionated by offline basic pH reversed-phase chromatography. Approximately 15 μg of peptides was loaded on to an in-house packed 320 μm × 25 cm column (3.5 μm particle size, Xbridge BEH C18; Waters Corporation, MA, United States). Liquid chromatography (LC) mobile phase buffers were composed of 10 mM ammonium formate (pH 7.9) and 90% (v/v) acetonitrile/10% (v/v) water. Peptides were eluted using a linear gradient of 5 to 50% over 45 min at a flow rate of 6 μl/min. Twelve concatenated fractions were then dried down prior to analysis. The TMT-labeled hydrophilic interaction liquid chromatography fractions were resuspended in mass spectrometry (MS) loading buffer [3% (v/v) acetonitrile/0.1% (v/v) formic acid] and analyzed online by nanocapillary LC–tandem mass spectrometry using a Dionex Ultimate 3000 HPLC system (Thermo Fisher Scientific, MA, United States) coupled to an in-house built fritless nano 75 μm × 30 cm column packed with ReproSil Pur 120 C18 stationary phase (1.9 μm, Dr. Maisch GmbH, Germany). Separated compounds were analyzed with an Orbitrap Eclipse Tribrid Mass Spectrometer (Thermo Fisher Scientific, MA, United States). A synchronous precursor selection MS3 method was used for data collection (*128*). Proteome Discoverer 2.5 (Thermo Fisher Scientific, MA, United States) was used to analyze the MS data; the settings included the SEQUEST HT database search engine node and percolator validation for protein identification. The abundance ratios for each sample were calculated against a pooled internal standard and normalized relative to total protein signal. Protein quantification values were exported for further analysis to Microsoft Excel (2016), and log(fold change) values were calculated.

### Statistical analysis

For statistical analysis, unless otherwise specified, all results obtained from independent experiments are reported as means ± SEM of multiple replicates. Comparisons between two groups of normally distributed and not connected data were performed using unpaired, nonparametric Student’s *t* test. Multiple group comparisons were performed by one-way analysis of variance (ANOVA) (for one independent variable) or two-way ANOVA (for two independent variables), followed by Tukey’s post hoc comparison (GraphPad Prism version 8.0, La Jolla, CA). Unless otherwise indicated, “*n*” in the figure legends represents the number of animals or independent biological samples per group used in the indicated experiments. *P* values of <0.05 were considered statistically significant. *P < 0.05, **P < 0.01, ***P < 0.001, ****P < 0.0001.

## Supplementary Materials

### This PDF file includes:

Supplementary Text Figs. S1 to S20 Legends for tables S1 to S10 References
