# Supplementary material for: Integration mapping of cardiac fibroblast single-cell transcriptomes elucidates cellular principles of fibrosis in diverse pathologies
Source: Sci Adv. 2024 Jun 21;10(25):eadk8501. doi: 10.1126/sciadv.adk8501 (PMC11192082; doi:10.1126/sciadv.adk8501)
Supplement: Supplementary file 1 — Supplementary Text Figs. S1 to S20 Legends for tables S1 to S10 References [file sciadv.adk8501_sm.pdf]

Science Advances
AAAS

Supplementary Materials for

Integration mapping of cardiac fibroblast single-cell transcriptomes elucidates cellular principles of fibrosis in diverse pathologies

Ralph Patrick *et al.*

Corresponding author: Ralph Patrick, ralph.patrick@imb.uq.edu.au; Richard P. Harvey, r.harvey@victorchang.edu.au

Sci. Adv. **10**, eadk8501 (2024)
DOI: 10.1126/sciadv.adk8501

The PDF file includes:

Supplementary Text
Figs. S1 to S20
Legends for tables S1 to S10
References

Other Supplementary Material for this manuscript includes the following:

Tables S1 to S10

Supplementary Text

<u>Extended methods</u>

Conventional echocardiography and Doppler imaging
Transthoracic echocardiography was performed using a VisualSonics Vevo 2100 system equipped with MS400 transducer (FUJIFILM VisualSonics, Canada). Anaesthesia was induced by 5% isoflurane and confirmed by lack of response to firm pressure on one of the hind paws. During the ultrasound acquisition—under body-temperature-controlled conditions—isoflurane was reduced to 1.0–1.5% and adjusted to maintain a heart rate in the range between 460 – 500 beats per min. LVEF and other indices of systolic function were obtained from short-axis B-mode scans at the midventricular level, as described (*129*). The areas boarded by the endocardium were planimetrically measured on all four SAX images at end diastole and end systole, and from this a modified Bullet formula was used to derive left ventricular ejection fraction (LVEF) (left ventricular volume [LVvol] = length [L] x mean endocardial area x 5/6). All parameters were measured at least three times, and means are presented.

Speckle-tracking echocardiography and strain analysis B-mode traces acquired from the parasternal long-axis view were used to calculate global strain in longitudinal dimensions using *Vevo Strain* software (FUJIFILM VisualSonics, Canada) and a speckle-tracking algorithm, as described (*78*).

Tail-cuff blood pressure recordings
Systolic blood pressure was measured noninvasively in conscious mice using the tail-cuff method and a CODA instrument (Kent Scientific, CT, USA). Mice were placed in individual holders on a temperature-controlled platform (37°C) and recordings were performed under steady-state conditions. Before testing, all mice were trained to become accustomed to short term restraint for a week prior. Blood pressure was recorded for at least four consecutive days and readings were averaged from ten measurements per session.

Histology, immunofluorescence staining and confocal microscopy
All samples were processed in parallel under identical conditions for comparisons. Most procedures were performed as described (*8*), except for skin and healthy ventricle tissue sections in which modifications were incorporated (see Supplementary Materials). Briefly, tissue samples were fixed in 4% PFA, rinsed in PBS, and immersed in PBS containing 30% sucrose at 4°C before being embedded in optimal cutting temperature compound (OCT) for cryosectioning. Sections were incubated in blocking buffer for 1 hour and then were incubated in primary antibodies diluted in blocking buffer overnight at 4°C followed by wash and incubation in appropriate fluorophore-conjugated secondary antibodies diluted in blocking buffer for 1 hour at room temperature. Sections were then washed and mounted in a mounting medium containing DAPI. IF images were captured using LSM900 Inverted confocal laser scanning microscope. For Picrosirius red staining, measurement of cardiomyocyte size and vessel density analysis, hearts were embedded in paraffin and later deparaffinized using standard protocols, and images were acquired using Olympus VS120 Slide Scanner. Primary antibodies and dilutions: PDGFRα (R&DSystems, USA; #AF1062, 1:100), DPPIV/CD26 (R&DSystems, USA; #AF954, 1:100), α-SMA (Sigma Aldrich, USA; #A5228, 1:400), α-SMA-Cy3 (Sigma-Aldrich, USA; #C6198, 1:250), Cardiac Troponin T-

Alexa Fluor 647 (BD Biosciences Pharmingen, USA; #565744, 1:100), Wheat Germ Agglutinin (WGA)-Alexa Fluor 647 (ThermoFisher, USA; #W32466, 1:100) and Isolectin B4 (IB4)-Alexa Fluor 488 (ThermoFisher, USA; #I21411, 1:100). All secondary antibody dilutions were 1:500.

Skin and mid-ventricle histology and staining

For isolating flank skin and ventricle mid-sections, 4-month-old adult male *Pdgfra*$^{eGFP/+}$ knock-in mice were anaesthetised by intraperitoneal injection of ketamine (100 mg/kg) and xylazine (20 mg/kg) and perfused via left ventricular cardiac puncture with 30 mL of PBS1X at a flow rate of 3 mL/min (Legato 100 Syringe Pump, Becton Dickinson Plasti-plak 50ml, & Nipro 25G catheter needle). The mice were again perfused with 30 mL of 4% PFA and collected hearts and shaved flank skin samples were fixed with 4% PFA (4% PFA in PBS, pH 7.4) for 3-hours at room temperature. Fixed tissues were washed briefly in PBS1x (three times, 5 min each wash) and then dehydrated with 30% sucrose solution over-night at room temperature. Hearts were cut at the axial midline and these halves were embedded side-by-side in Tissue-Tek Optimal Cutting Temperature mounting medium (O.C.T., Tissue-Tek, Sakura) and stored at -80°C. Heart sections were cut in the axial orientation on a Leica CM1950 clinical cryostat (Leica Biosystems) at 7-µm thickness and collected onto Trajan Series 2 Adhesive slides (Trajan Scientific & Medical). Flank skin samples was embedded and cut in the same manner, at transverse orientation with nitrocellulose membrane acting as a structural support. For indirect immunofluorescence staining, tissue sections were permeabilized in 1× saponin-based permeabilization and wash buffer (0.2% (w/v) saponin containing 4% (v/v) FBS (v/v), 1% (w/v) BSA and 0.02% (v/v) Sodium Azide in PBS) for 15 min. Tissues were then stained over-night at 4°C in 1× saponin-based permeabilization and wash buffer using anti-Mouse DPPIV/CD26 Antibody (DPP4 polyclonal goat IgG, cat. no. AF954, 1:100 dilution), alpha-smooth muscle actin (α-SMA)-Cy3 (1:250 dilution, clone 1A4, Cat. No. C6198, Sigma-Aldrich), and Alexa Fluor® 647 Mouse Anti-Cardiac Troponin T (BD Pharmingen, cat. no. 565744, AB_2739341, Clone 13-11, 1:100 dilution). Following brief washing in PBS1x (3X 5 min washes), DPP4 stained sections were counterstained with suitable species-specific secondary antibody coupled to Alexa Fluor dye (1:500, Donkey anti-Goat IgG (H+L) Cross-Adsorbed Secondary Antibody, Alexa Fluor 633) diluted in 1× saponin-based permeabilization and wash buffer for 2-hours at room temperature. Sections were washed again in 1X PBS (3X 5 min washes), and stained with Vybrant™ DyeCycle™ Violet (ThermoFisher, Cat# V35003) at 1µM for 10 minutes in 1× saponin-based permeabilization and wash buffer, washed once with PBS1x and mounted under coverslip (Menzel-Gläser, 22x22 mm, #0). Dako fluorescent mounting medium (Agilent, Cat# S3023) was applied to the slides prior mounting and imaging. The endogenous fluorescence of eGFP was detected.

Confocal laser-scanning microscopy and image processing

z-stack high-resolution confocal microscopy of stained sections was performed using a LSM900 Inverted confocal laser scanning microscope that comprises an upright Zeiss Axio Observer 7, four laser lines, two Gallium Arsenide Phosphide photomultiplier tubes (GaAsP-PMT), and a motorised stage. Confocal tiling scan was employed using a ×10 objective, 0.45 numerical aperture 512 µm × 512 µm, WD 2.0, Plan-APO UV-VIS–NIR, and PBS immersion. For DPP4 z-stack imaging, we used a ×63 objective, 1.4 numerical aperture with a z-step size of 1 µm, 2,048 µm × 2,048 µm, WD 0.19, Plan-APO DIC-UV-VIS-IR, and oil immersion (Immersol 518 F, Refractive Index: 1.518, Zeiss). Whenever comparisons were made, the settings for confocal scanner detection and laser excitation were kept identical between samples. Z-stacks were processed using

Fiji software (ImageJ2, V2.14.0/1.54f), and finally, distinct LUTs were applied to each image to improve the visibility of the different channels.

Cardiomyocyte size and vessel density analysis
To analyse cardiomyocyte size (as a surrogate measure of hypertrophy) and vessel density, ventricle sections stained with WGA and IB4 were imaged at 40x objective magnification and analysed as previously described (*130*). Pre-processing was carried out using *ImageJ* followed by analysis of cardiomyocyte size and vessel density on *CellProfiler* (v4.2.4). Cardiomyocyte size was normalised to the normal chow control group whilst vessel density was calculated as number of vessels per cardiomyocyte and normalised to the normal chow control group.

Measurement of lung weight
Ratio of wet to dry lung weight was estimated as described previously (*78*).

Volcano plot and GO term analysis
For proteomics, volcano plots were generated using the *RStudio* package *EnhancedVolcano*. Data generated during differential expression analysis of proteins in HFpEF vs Chow mice was plotted using cut offs of 0.5 for fold change and 0.05 for adjusted *p*-value. Downregulated and upregulated proteins were then run separately through the Database for Annotation, Visualisation, and Integrated Discovery (*DAVID*, david.ncifcrf.gov), using the preselected databases for pathway analysis. Both up and downregulated pathways were visualised together using *RStudio* package *ggplot2*, with the number of DE genes present in that pathway represented by circle size, with adjusted *p*-value (Benjamini<0.05) represented by a spectrum colour range.

Cell Isolation
Total interstitial cell population (TIP) were isolated as previously described (*8*). Briefly, hearts were minced and incubated in collagenase type II (Worthington) at 37°C before filtering through 40 mm strainers. Cells were resuspended in red cell lysis buffer, followed by dead cell removal, immunostaining for 15min on ice with indicated fluorophore-conjugated antibodies and two times wash with fluorescence-activated cell sorting (FACS) buffer (2% FBS, 2mM EDTA in PBS, pH 7.4) before the acquisition.

Flow Cytometry
FSC-H versus FSC-A, FSC-H versus FSC-W and SSC-H versus SSC-W cytograms were used to discriminate and gate out doublets/cell aggregates during sorting or analysis. DAPI was used to distinguish live (DAPI-) and dead (DAPI+) cells. For most samples, at least 10,000 final gate events were collected and stored. Flow cytometry experiments were performed on a Canto II (BD Biosciences, USA), using *FACSDiva* software (BD Biosciences, USA). Lineage negative ($CD31^-$/$CD45^-$) *Pdgfra*-$eGFP^+$ CF flow cytometry was performed using a BD FACSymphony A5. FACS analysis was performed using *FlowJo* software (version 10.8.1, BD). For details of DPP4, SCA-1, CD90.2 and CD55 staining in live $CD31^-$/$CD45^-$ *Pdgfra*-$eGFP^+$ CF, flow cytometry events, and further gating, see Supplementary Material. Antibodies and dilutions: Fc Block (CD16/32) (BioLegend, USA; #101301, 1:400), Ly-6A/E (SCA-1)-APC/Cy7 (BD Biosciences Pharmingen, USA; # 560654, 1:400), CD90.2 (Thy1.2)-APC/Cy7, (BioLegend, USA; # 105327, 1:200), CD26 (DPP4)-APC (BioLegend, USA; # 137807, 1:100), CD55-PE REAfinity (Miltenyi Biotech, USA; # 130-104-055, 1:40), BD OptiBuild™ BUV395 Rat Anti-Mouse CD31 (BD Biosciences

Pharmingen, USA; Clone 390, 1:400), CD45-BV786 (BD Biosciences Pharmingen, USA; # 564225, Clone 30-F11, 1:400), CD45-APC/Cy7 (BD Biosciences Pharmingen, USA; # 557659, 1:400).

Heart ventricle cell isolation and high-speed flow cytometry of live *Pdgfra-eGFP*$^{+}$ CF

Two-step digestion of heart tissue for cellular isolation was performed as described before with few modifications (*131*). Hearts of 4-month-old adult male *Pdgfra*$^{eGFP/+}$ knock-in mice were carefully dissected, washed in ice-cold DMEM, ventricles were further separated transversally, and cut into small pieces with blades until a homogeneous, paste-like slurry was formed. Seven ml of digestion solution containing collagenase type II (265 Unit/mL, Worthington, DC, United States), 0.5 U of Dispase (Cat. No. 07913, STEMCELL Technologies, Canada), 0.05 mg/mL of DNaseI (Cat. No. 10104159001, Roche/Sigma-Aldrich, 100 mg from bovine), and 1% BSA (Sigma-Aldrich Pty Ltd., A3311-50G) dissolved in DMEM (Cat. No. 10566016) was added and the preparation was placed on a water bath with constant rotation at 37°C for 40 min. After 20 min of incubation, 10 mL of FACS buffer was added to the ventricle preparations and the homogenate was gently pipetted up and down 10 times to enhance dissociation with a 10 mL serological Stripette® on ice. Ice-cold FACS buffer was added to make the final volume up to 20 mL volume and the supernatant was then passed sequentially through 100 µm and 70 µm cell strainers. The remaining non-digested pellet was incubated with additional 4 mL of digestion buffer (detailed above) for another 20 min in a water bath. The first filtered preparation was centrifuged at 600 g for 6 min at 4°C, and the flicked pellet was resuspended in 5 mL of ACK lysing buffer (i.e., red blood cell lysis buffer in w/v) (potassium carbonate 0.01%, ammonium chloride 0.083%, and EDTA 0.0004% in H_2Od at pH 7.4) and further incubated for 3 min. Then, 20 mL of FACS buffer was added to stop the lysis and cell pelleting was carried out as described before. After flicking, 5 mL of FACS was added to the cell and kept on ice. After further digestion of the remaining non-digested pellet, the cells were collected as described above for the first dissociation step. At the end of the process, the two dissociated cell mixtures were pooled together in 10 mL of FACS buffer, which was further divided into 1.5 mL tubes, cells pelleted at 600 g for 5 min at 4°C and flow cytometry staining was carried out in 400 µL of FACS buffer, after the mixture was incubated with Fc block antibody for 15 min on ice.

Each sample was ran using a BD FACSymphony A5 high-speed cell analyser, with 2 million total events captured, and at least thousand *Pdgfra-eGFP*$^{+}$ CF being analysed. Flow cytometry measurements were run at a mid-flow rate, and the core stream allowed to stabilize for 5 s prior acquisition. Flow cytometry of resting and live lineage-negative (CD31^{-}/CD45^{-}) *Pdgfra-eGFP*$^{+}$ ventricular CF was performed after staining with specific antibodies detailed in the main text, and DAPI negative cells were selected and gated for eGFP fluorescence. Unstained and isotype controls were used in each combination for DPP4-APC, SCA-1-APC/Cy7, CD90.2-APC/Cy7, and CD55-PE. Fluorescence data was collected and further compensated using FacsDIVA 8 software. Fluorescence data was analysed after biexponential transformation in FlowJo Portal (version 10.8.1, Becton Dickinson & Company (BD)) using Mac OS X operating system.

Cell/nuclear isolation and sequencing - HFpEF model

For the HFpEF model, we adapted a protocol for simultaneous isolation of CM nuclei and non-myocyte cells, as previously described (*63*). TIP cells were isolated as above. For CM nuclei extraction, strainers were washed with FACS buffer to collect cardiomyocyte in a 50-ml Falcon

tube. After 2 rounds of centrifugation (500g x 5 min), CM nuclei were isolated by swelling CMs in a hypotonic solution (91 mM NaCl, 5.3 mM KCl, 0.5 mM MgCl2, 0.293 mM CaCl2, 10 mM glucose, 10 mM HEPES at pH 7.4 and 0.05% Triton-X-100 supplemented with 40 U/µl RNasin® Ribonuclease Inhibitor (Promega Corporation, WI, USA) before triturating cells using a 30-gauge hypodermic needle to mechanically fragment cells. Nuclei were then isolated by filtration through a 40 µm cell strainer to exclude large debris. Finally, nuclei suspension was centrifuged at (500g x 5 min) before staining with DAPI, followed by FACS for isolation of nuclei. TIP cells and CM nuclei were FACS-isolated and subsequently pooled at specific proportions (50% TIP (without $CD31^{+}$ cells), 20% $CD31^{+}$ cells and 30% CM nuclei). Isolation of single cells was performed in two batches on separate days, with samples from each treatment group split across days to mitigate batch effects. Cells/nuclei from 2 mice were pooled together after FACS isolation and considered as one replicate and 2 replicates per condition were run for sequencing.

FACS was used to isolate CM nuclei from each heart and enumerated using a hemocytometer for single cell RNA sequencing workflows using a 10x Genomics platform. Approximately 6000 total nuclei (per condition) were loaded into each channel and captured on the 10x Genomics Chromium Controller using the Chromium Single Cell 3' v3.1 reagent kit. Following capture and reverse transcription, cDNA was synthesized and amplified, then final Illumina compatible sequencing libraries generated. Libraries were sequenced to an approximate depth of 30,000 reads per cell on an Illumina S4 flow cell, using the Illumina NovaSeq 6000 Sequencer. Isolation of single cells was performed in two batches on separate days, with samples from each treatment group split across days to mitigate batch effects.

Analysis of human spatial transcriptomic data - fibroblast clustering

First, we took the integrated mouse CF data and removed types F-WntX, IR, and F-IFNS. To relate the mouse and human genes, we used the orthogene package (version 1.6.1) to convert mouse genes into human homologues. We used the findMarkers function in the scran package (version 1.28.2) to identify genes that were significantly associated with each type with FDR-adjusted P-value < 0.05 and log fold-change > 0.25. Then, we downloaded the Kuppe and colleagues' snRNA-seq and Visium data (*21*), in the form of gene expression log-count matrices, from the publication's data availability URL. The Visium data had already been filtered to remove spots with no or low gene expression. For the Visium data we also extracted the spatial location information for each of the Visium resolved spots. For the snRNA-seq data, we restricted to cells that were annotated as Fibroblast, and kept only genes that were detected in at least 1% of cells. We selected 500 top highly variable genes and used these to perform multi-batch principal component analysis using the batchelor package (version 1.16.0), followed by batch correction according to the sequencing samples using the reduced mutual nearest neighbour approach in the batchelor package. We then performed unbiased clustering of these cells using the clusterRows function in the bluster package (version 1.10.0) using the two-step param approach, where the first step was K-means clustering with 500 centres, followed by louvain graph-based clustering over the 500 centres. We then performed k-nearest neighbour-based smoothing of the cluster labels with k=100 nearest neighbours. For visualisation, we performed further dimensionality reduction using UMAP via the runUMAP function in scater (version 1.28.0). We used the same strategy as the mouse data to identify genes significantly associated with each human fibroblast cluster. We used the topGO function in the limma package (version 3.56.2) to assess the significance of enrichment of gene ontology terms for each of the human fibroblast clusters. To assess the concordance of marker

genes among each mouse and human cluster, we calculated the Jaccard index of the identified genes for each pair of clusters and visualised this in a heatmap.

Analysis of human spatial transcriptomic data - MYO and MFC scores

We performed a differential expression analysis comparing the human fibroblast cluster H1 and H6 using the findMarkers function in the scran package, using genes that were detected in at least 20% of cells within H1 and H6. We had observed that several genes that were positive markers for cluster H1 were also markers of cardiomyocytes, thus, we performed an additional differential expression analysis between the cluster H1 and the snRNA-seq resolved cells that were annotated as Cardiomyocytes. To extract a MYO-specific score, we assigned a weighting to each gene corresponding to the log fold-change of cluster H1 compared to cluster H6 and reassigned the weighting to zero for genes with a log fold-change below 0.1 as well as for genes with a positive log fold-change for Cardiomyocyte cells versus cluster H1. To extract a MFC-specific score, we performed a similar weighting scheme with a log fold-change threshold of 0.5 and no further reassignment with Cardiomyocytes. To extract a score for each gene expression profile, we performed elementwise multiplication of each gene's expression with the weighting for the MYO and MFC identities respectively, both for the snRNA-seq resolved cells and for the Visium-resolved spots. We checked the MYO and MFC scores were highest among Fibroblasts by generating boxplots for all cell types within the snRNA-seq data. We visualised the MYO and MFC scores in the Visium data using bivariate colour plots for spots that were annotated as Fibroblast in the original publication. Finally, we calculated total MYO and MFC scores for each patient Visium sample by calculating the sum of each MYO and MFC score among Fibroblast spots and divided by the total number of spots identified.

Figure S1

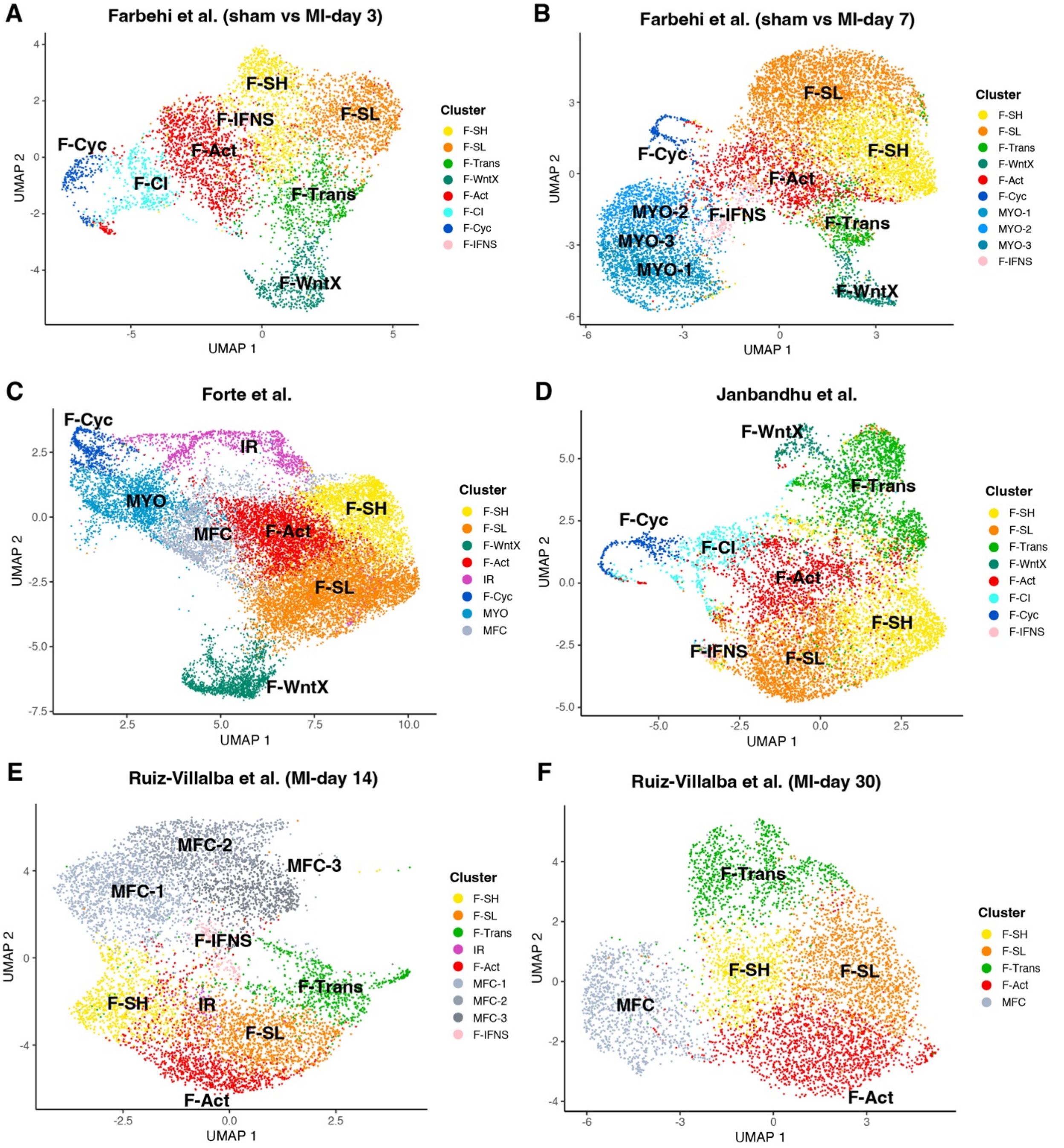

Fig. S1. Clustering and cell type assignments for the MI datasets included in the integration analysis. (A-F) Fibroblast sub-types were assigned based on marker genes in accordance with foundational scRNA-seq publications (see **Table 1**). (A) sham and MI-day 3. (B) sham and MI-day 7 data from Farbehi and colleagues utilising the cluster identifications from the original publication. (C) re-clustering analysis of uninjured and MI-days 1-28 data from and colleagues. (D) Sham and MI-day 3 data from Janbandhu and colleagues using the population identities from the original publication. Note this was derived using a label transfer analysis from (A). (E, F) Clustering of MI-day 14 (E) and MI-day 30 data (F) from Ruiz-Villalba and colleagues.

Figure S2

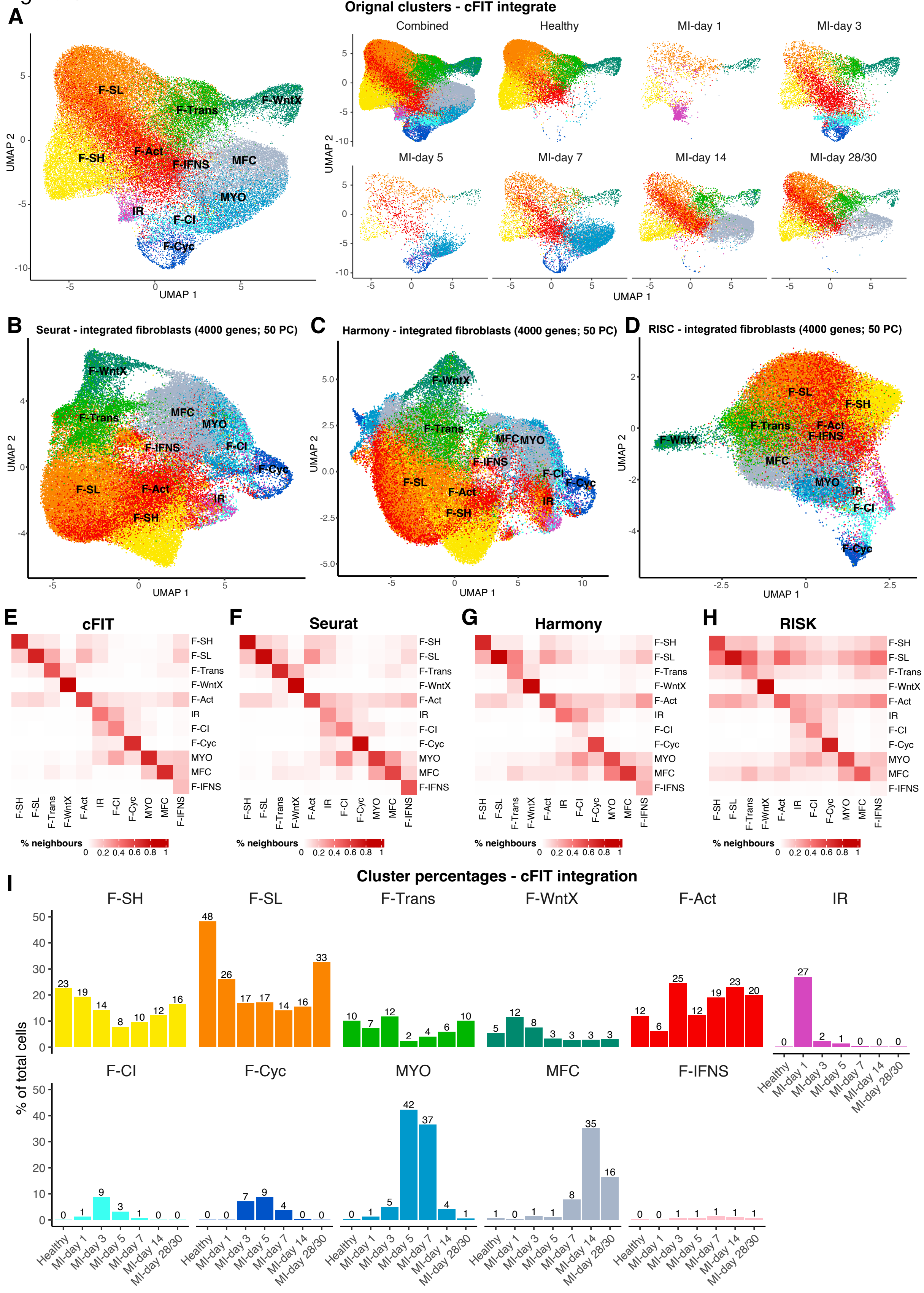
A
Orignal clusters - cFIT integrate
Combined
Healthy
MI-day 1
MI-day 3
MI-day 5
MI-day 7
MI-day 14
MI-day 28/30
UMAP 1
UMAP 2
F-SL
F-Trans
F-WntX
F-SH
F-Act
F-IFNS
MFC
MYO
IR
F-CI
F-Cyc
B
Seurat - integrated fibroblasts (4000 genes; 50 PC)
C
Harmony - integrated fibroblasts (4000 genes; 50 PC)
D
RISC - integrated fibroblasts (4000 genes; 50 PC)
E
cFIT
F
Seurat
G
Harmony
H
RISK
% neighbours
I
Cluster percentages - cFIT integration
% of total cells
Healthy
MI-day 1
MI-day 3
MI-day 5
MI-day 7
MI-day 14
MI-day 28/30

Fig. S2. Integration of fibroblasts across MI timepoints and datasets. (A) UMAP plot based on cFIT integration with cells coloured according to their dataset-specific cluster assignments. Shown is an aggregate of CFs across conditions or according to injury timepoint. (B-D) UMAP plots based on alternative integration approaches, (B) Seurat, (C) Harmony and (D) RISC. In all cases, the top 4000 variable genes were used as for the cFIT integration analysis, and the first 50 PCs. (E-H) Nearest neighbour analysis among the cell clusters for the indicated integration method. For each cluster (by column), the average percentage of nearest 200 neighbours among all clusters was determined. (I) Percentage of cells following cFIT integration in each population according to experimental condition.

Figure S3

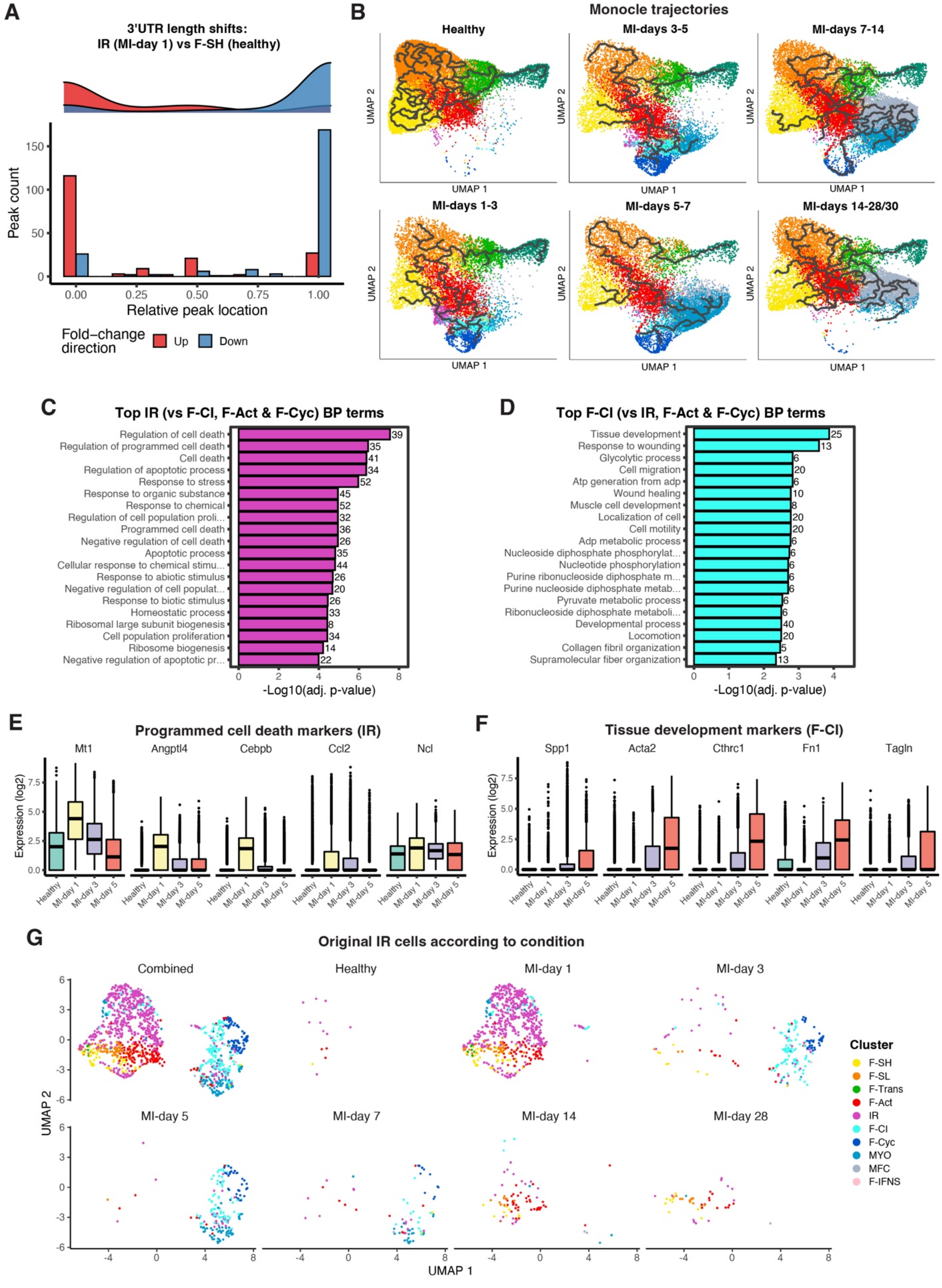
A
3'UTR length shifts:
IR (MI-day 1) vs F-SH (healthy)
Peak count
Relative peak location
Fold−change direction
Up
Down
B
Monocle trajectories
Healthy
MI-days 3-5
MI-days 7-14
MI-days 1-3
MI-days 5-7
MI-days 14-28/30
UMAP 1
UMAP 2
C
Top IR (vs F-Cl, F-Act & F-Cyc) BP terms
Regulation of cell death
Regulation of programmed cell death
Cell death
Regulation of apoptotic process
Response to stress
Response to organic substance
Response to chemical
Regulation of cell population proli...
Programmed cell death
Negative regulation of cell death
Apoptotic process
Cellular response to chemical stimu...
Response to abiotic stimulus
Negative regulation of cell populat...
Response to biotic stimulus
Homeostatic process
Ribosomal large subunit biogenesis
Cell population proliferation
Ribosome biogenesis
Negative regulation of apoptotic pr...
-Log10(adj. p-value)
D
Top F-Cl (vs IR, F-Act & F-Cyc) BP terms
Tissue development
Response to wounding
Glycolytic process
Cell migration
Atp generation from adp
Wound healing
Muscle cell development
Localization of cell
Cell motility
Adp metabolic process
Nucleoside diphosphate phosphorylat...
Nucleotide phosphorylation
Purine ribonucleoside diphosphate m...
Purine nucleoside diphosphate metab...
Pyruvate metabolic process
Ribonucleoside diphosphate metaboli...
Developmental process
Locomotion
Collagen fibril organization
Supramolecular fiber organization
-Log10(adj. p-value)
E
Programmed cell death markers (IR)
Mt1
Angptl4
Cebpb
Ccl2
Ncl
Expression (log2)
Healthy
MI-day 1
MI-day 3
MI-day 5
F
Tissue development markers (F-Cl)
Spp1
Acta2
Cthrc1
Fn1
Tagln
G
Original IR cells according to condition
Combined
Healthy
MI-day 1
MI-day 3
MI-day 5
MI-day 7
MI-day 14
MI-day 28
UMAP 1
UMAP 2
Cluster
F-SH
F-SL
F-Trans
F-Act
IR
F-Cl
F-Cyc
MYO
MFC
F-IFNS

Fig. S3. Analysis of IR and F-CI cell populations. (A) Sierra plot of relative shifts in 3'UTR length between IR at MI-day 1 and F-SH from healthy hearts. (B) UMAP plot split according to successive pairs of MI time points in comparison to the combined healthy control, with *Monocle* trajectories overlaid on UMAP coordinates. (C,D) Top 20 GO Biological Process terms for DE genes upregulated (MAST testing; $p_{adj} < 1\times10^{-05}$; log2 fold change > 0.5) in (C) IR compared to F-CI, F-Act and F-Cyc combined at MI-days 1 & 3 or (D) F-CI compared to IR, F-Act and F-Cyc combined at MI-days 1 & 3. (E) Gene expression across all fibroblasts by timepoint for representative genes from the *programmed cell death* GO term over-represented in genes upregulated in IR. (F) Gene expression across all fibroblasts by timepoint for representative genes from the *tissue development* GO term over-represented in genes upregulated in F-CI. (G) UMAP according to experimental condition of IR cells as identified by initial clustering of the Forte and colleagues' data (*10*), with updated cell labels following the kNN analysis incorporating all MI datasets.

Figure S4

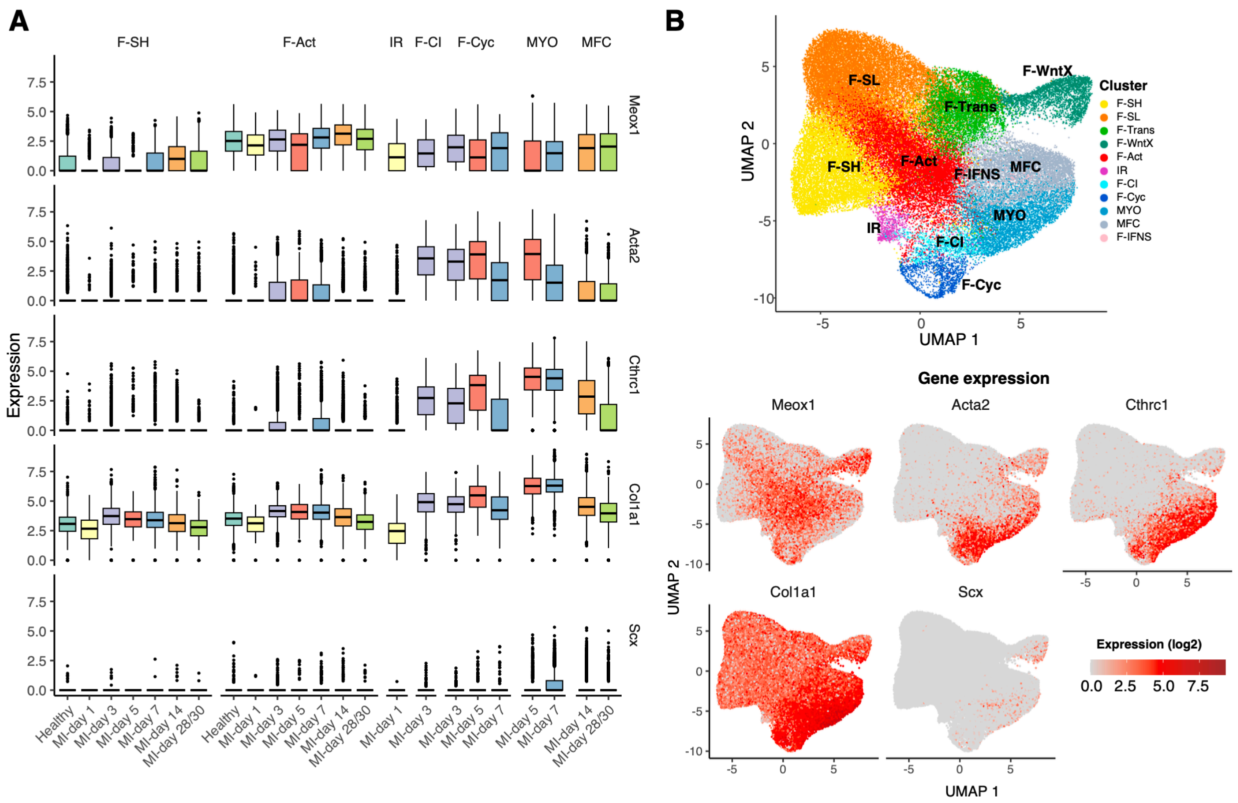

Fig. S4. Analysis of MI expression dynamics. (A) Expression of select fibroblast activation or myofibroblast markers across fibroblast sub-types at the indicated MI timepoint or uninjured control. (B) Expression of select fibroblast activation and myofibroblast markers in an aggregate of healthy and MI cells.

Figure S5

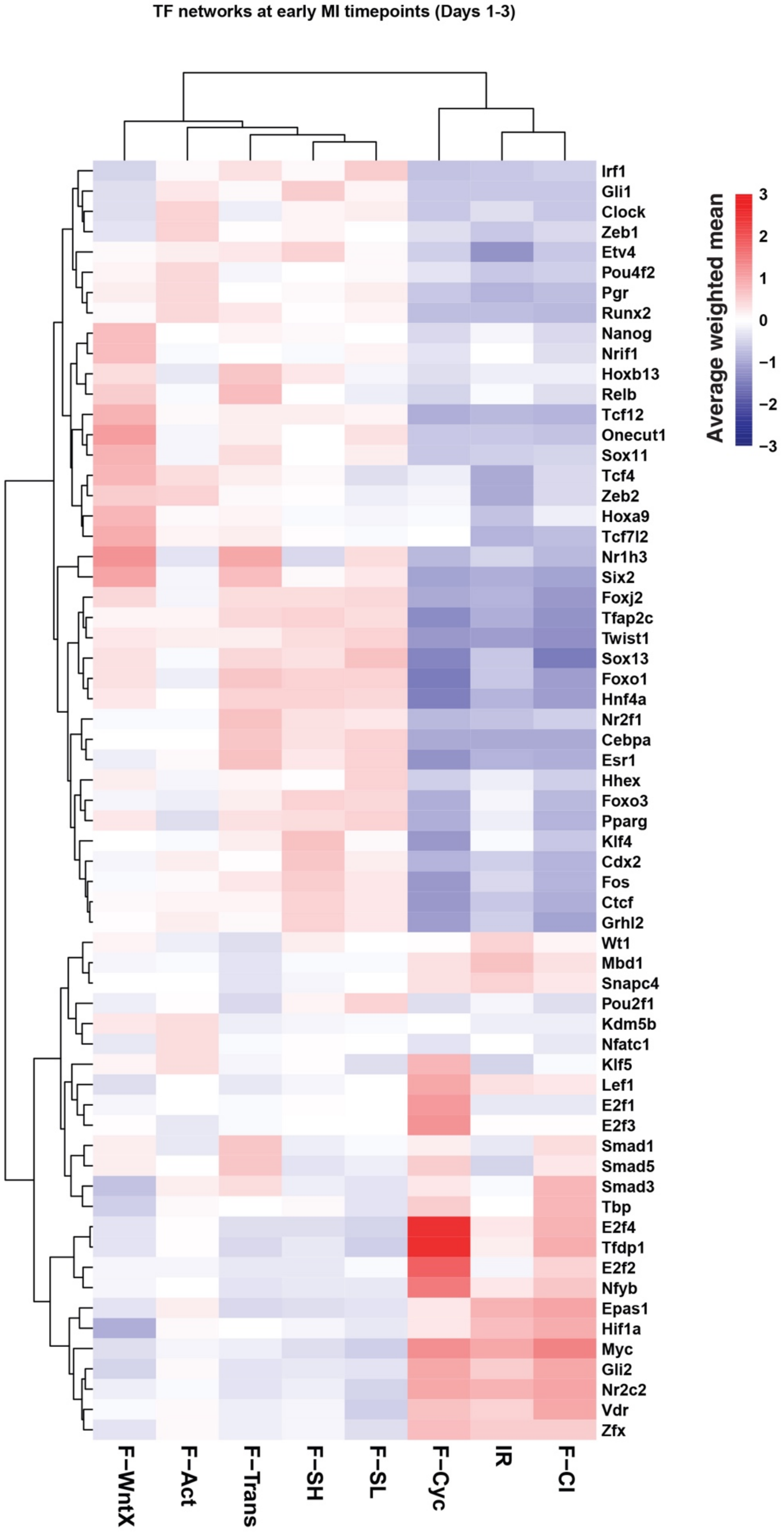
TF networks at early MI timepoints (Days 1-3)
Irf1
Gli1
Clock
Zeb1
Etv4
Pou4f2
Pgr
Runx2
Nanog
Nrif1
Hoxb13
Relb
Tcf12
Onecut1
Sox11
Tcf4
Zeb2
Hoxa9
Tcf7l2
Nr1h3
Six2
Foxj2
Tfap2c
Twist1
Sox13
Foxo1
Hnf4a
Nr2f1
Cebpa
Esr1
Hhex
Foxo3
Pparg
Klf4
Cdx2
Fos
Ctcf
Grhl2
Wt1
Mbd1
Snapc4
Pou2f1
Kdm5b
Nfatc1
Klf5
Lef1
E2f1
E2f3
Smad1
Smad5
Smad3
Tbp
E2f4
Tfdp1
E2f2
Nfyb
Epas1
Hif1a
Myc
Gli2
Nr2c2
Vdr
Zfx
F−WntX
F−Act
F−Trans
F−SH
F−SL
F−Cyc
IR
F−CI
Average weighted mean
3
2
1
0
−1
−2
−3

Fig. S5. Predicted TF regulators for MI-days 1-3. Top ten predicted TFs for each indicated population for MI-days 1-3 cells. Shown are the average weighted mean decoupleR scores across the cells for each population.

Figure S6

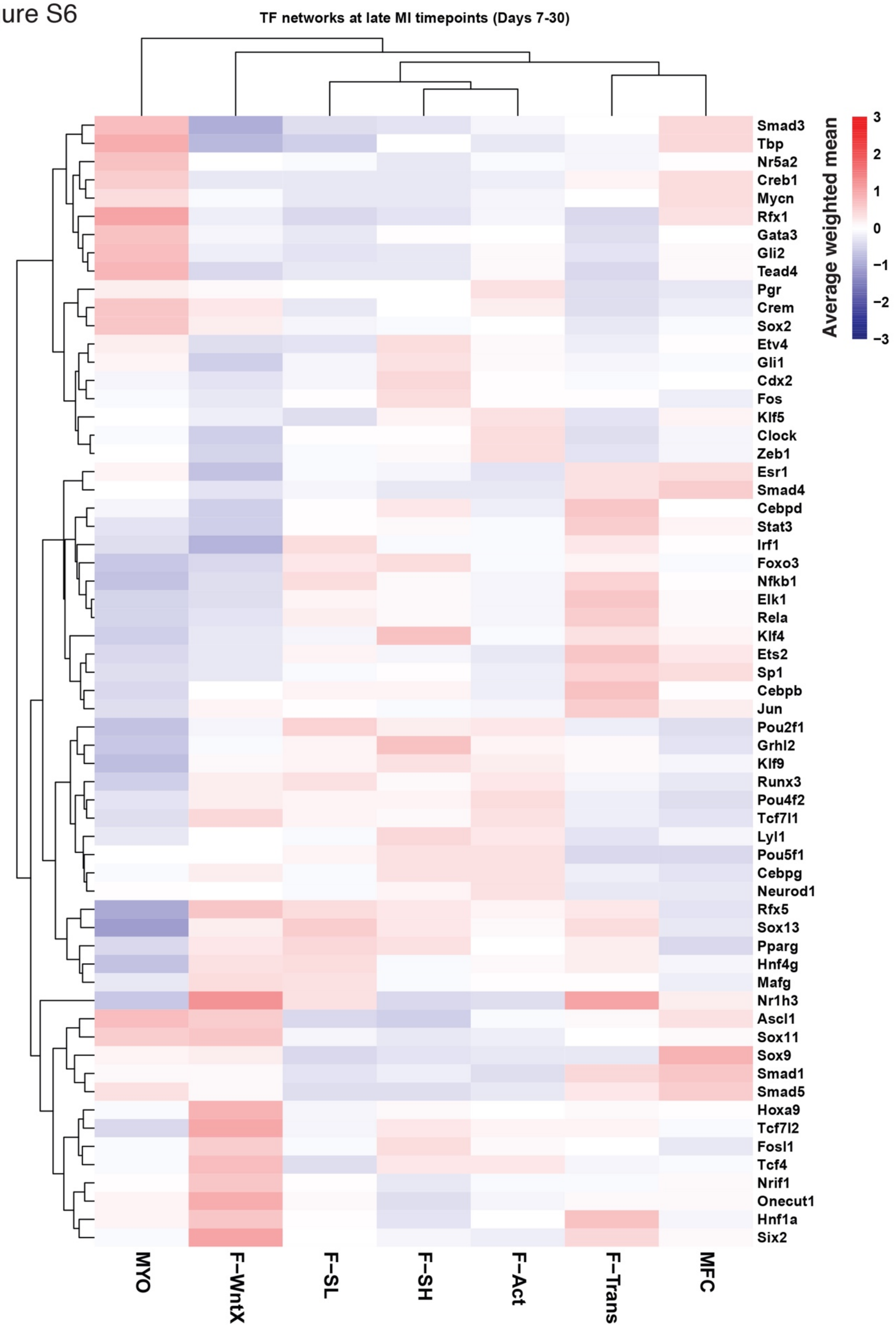
TF networks at late MI timepoints (Days 7-30)
Smad3
Tbp
Nr5a2
Creb1
Mycn
Rfx1
Gata3
Gli2
Tead4
Pgr
Crem
Sox2
Etv4
Gli1
Cdx2
Fos
Klf5
Clock
Zeb1
Esr1
Smad4
Cebpd
Stat3
Irf1
Foxo3
Nfkb1
Elk1
Rela
Klf4
Ets2
Sp1
Cebpb
Jun
Pou2f1
Grhl2
Klf9
Runx3
Pou4f2
Tcf7l1
Lyl1
Pou5f1
Cebpg
Neurod1
Rfx5
Sox13
Pparg
Hnf4g
Mafg
Nr1h3
Ascl1
Sox11
Sox9
Smad1
Smad5
Hoxa9
Tcf7l2
Fosl1
Tcf4
Nrif1
Onecut1
Hnf1a
Six2
MYO
F−WntX
F−SL
F−SH
F−Act
F−Trans
MFC
Average weighted mean
3
2
1
0
−1
−2
−3

Fig. S6. Predicted TF regulators for MI-days 7-30. Top ten predicted TFs for each indicated population for MI-days 7-30 cells. Shown are the average weighted mean decoupleR scores across the cells for each population.

Figure S7

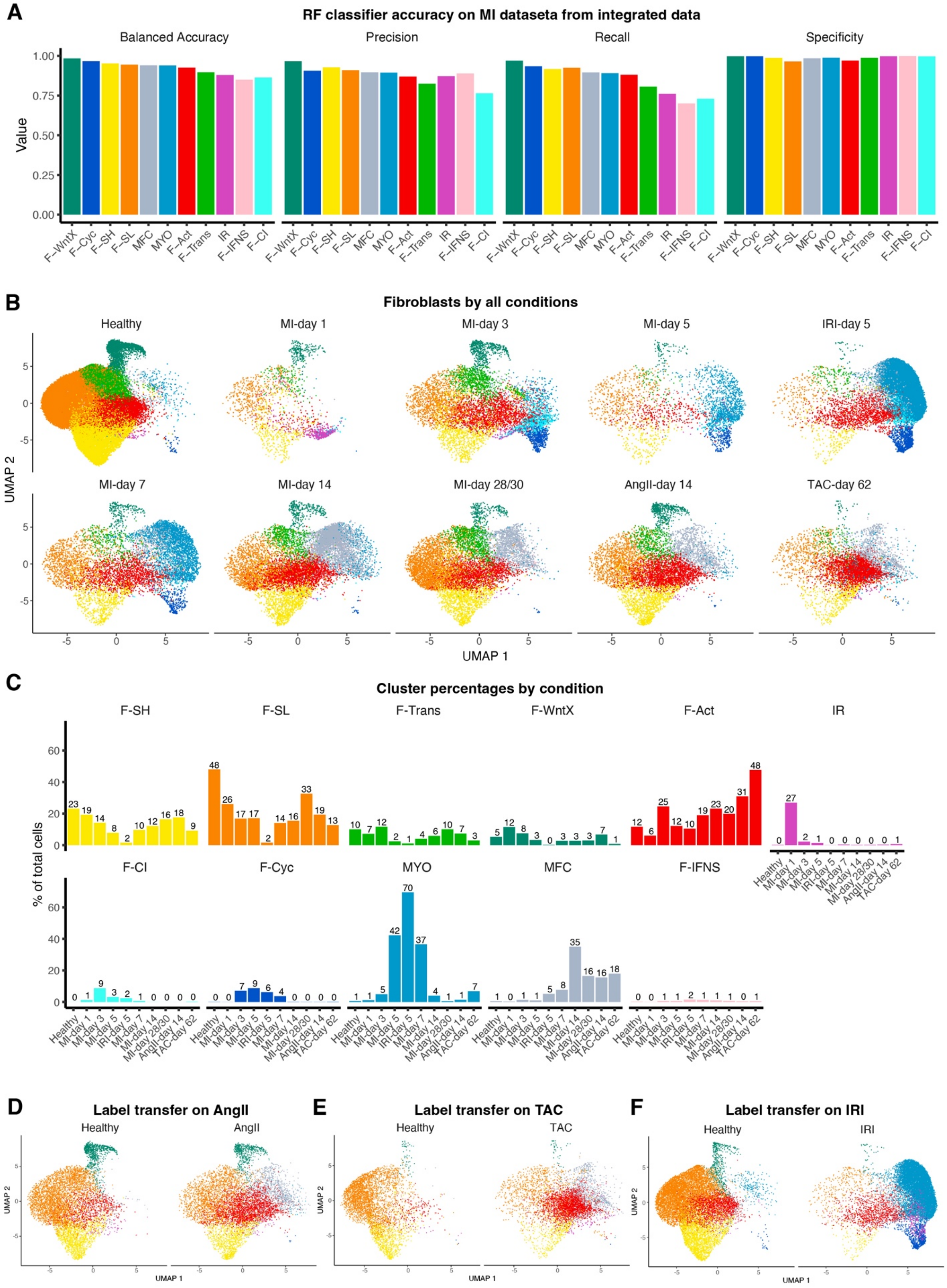
A
RF classifier accuracy on MI dataseta from integrated data
Balanced Accuracy
Precision
Recall
Specificity
Value
1.00
0.75
0.50
0.25
0.00
F−WntX F−Cyc F−SH F−SL MFC MYO F−Act F−Trans IR F−IFNS F−CI
B
Fibroblasts by all conditions
Healthy
MI-day 1
MI-day 3
MI-day 5
IRI-day 5
MI-day 7
MI-day 14
MI-day 28/30
AngII-day 14
TAC-day 62
UMAP 2
UMAP 1
C
Cluster percentages by condition
F-SH
F-SL
F-Trans
F-WntX
F-Act
IR
F-CI
F-Cyc
MYO
MFC
F-IFNS
% of total cells
F-SH: 23 19 14 8 2 10 12 16 18 9
F-SL: 48 26 17 17 2 14 16 33 19 13
F-Trans: 10 7 12 2 1 4 6 10 7 3
F-WntX: 5 12 8 3 0 3 3 3 7 1
F-Act: 12 6 25 12 10 19 23 20 31 48
IR: 0 27 2 1 0 0 0 0 0 1
F-CI: 0 1 9 3 2 1 0 0 0 0
F-Cyc: 0 0 7 9 6 4 0 0 0 0
MYO: 1 1 5 42 70 37 4 1 1 7
MFC: 1 0 1 1 5 8 35 16 16 18
F-IFNS: 0 0 1 1 2 1 1 1 0 1
Healthy MI-day 1 MI-day 3 MI-day 5 IRI-day 5 MI-day 7 MI-day 14 MI-day 28/30 AngII-day 14 TAC-day 62
D
Label transfer on AngII
Healthy
AngII
E
Label transfer on TAC
Healthy
TAC
F
Label transfer on IRI
Healthy
IRI
UMAP 2
UMAP 1

Fig. S7. Cross-disease integration of fibroblast datasets. (A) Prediction accuracy metrics for predicting fibroblast cell types from the integrated MI dataset using a Random Forest (RF) classifier. Evaluations are based on 10-fold cross-validation. (B) UMAP of cross-disease datasets and timepoints according to condition. Fibroblast cell types in the non-MI conditions predicted using the RF classifier. (C) Proportion of fibroblast cell types according to experimental condition and timepoint (D-F) Seurat label transfer analysis from the integrated MI dataset to cross-disease datasets (D) AngII, (E) TAC and (F) IRI.

Figure S8

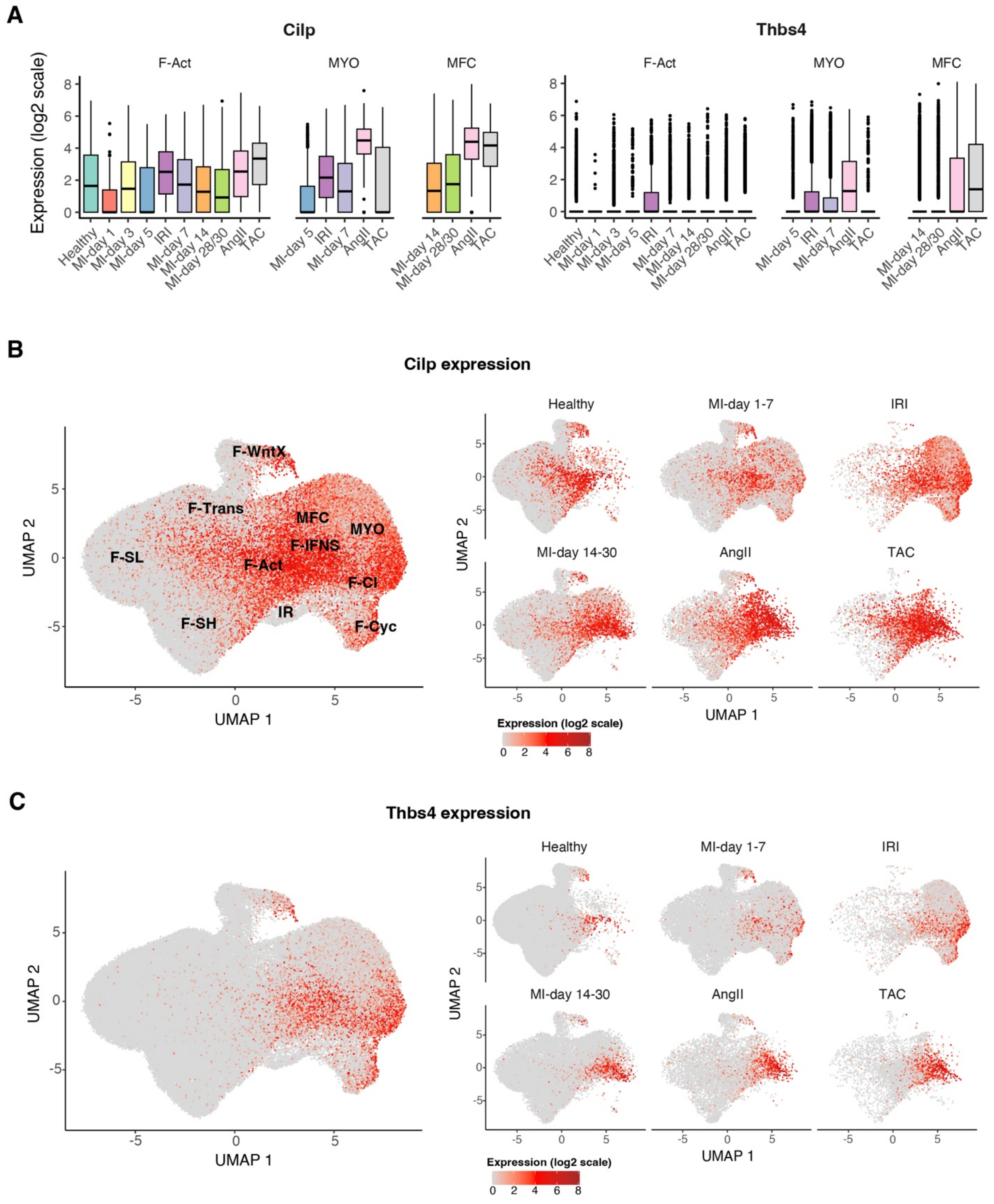
A
Cilp
Thbs4
F-Act
MYO
MFC
Expression (log2 scale)
Healthy
MI-day 1
MI-day 3
MI-day 5
IRI
MI-day 7
MI-day 14
MI-day 28/30
AngII
TAC
B
Cilp expression
F-WntX
F-Trans
MFC
MYO
F-IFNS
F-SL
F-Act
F-CI
IR
F-SH
F-Cyc
UMAP 1
UMAP 2
Healthy
MI-day 1-7
IRI
MI-day 14-30
AngII
TAC
Expression (log2 scale)
C
Thbs4 expression
UMAP 1
UMAP 2
Healthy
MI-day 1-7
IRI
MI-day 14-30
AngII
TAC
Expression (log2 scale)

Fig. S8. ***Cilp*** **and** ***Thbs4*** **expression in different CF subpopulations.** (A) Box plots showing expression in F-Act, MYO and MFC. (B,C) UMAPs showing expression across an aggregate MI time course (including healthy hearts; left panels) alongside those for individual conditions.

Figure S9

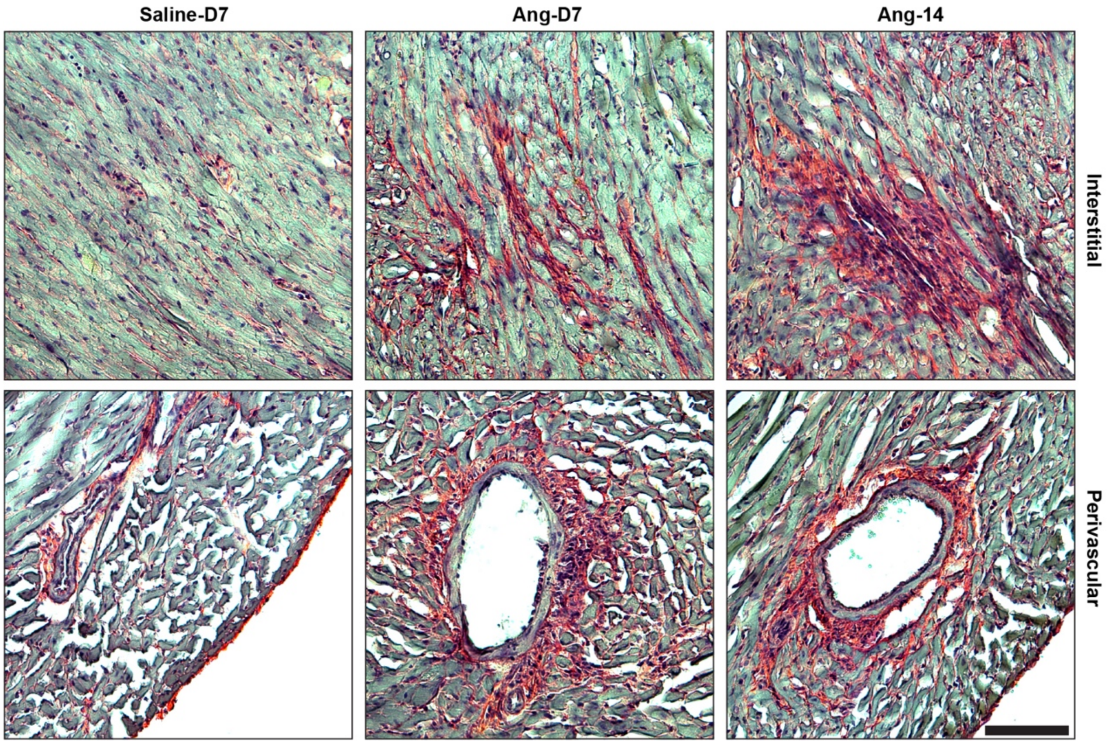

Fig. S9. Documentation of fibrosis in AngII model. Representative images of heart sections at indicated timepoints after AngII treatment showing collagen staining using Picrosirius red (n=3). Top panels – interstitial foci; bottom panels – perivascular foci. Scale bar, 100 μm.

Figure S10

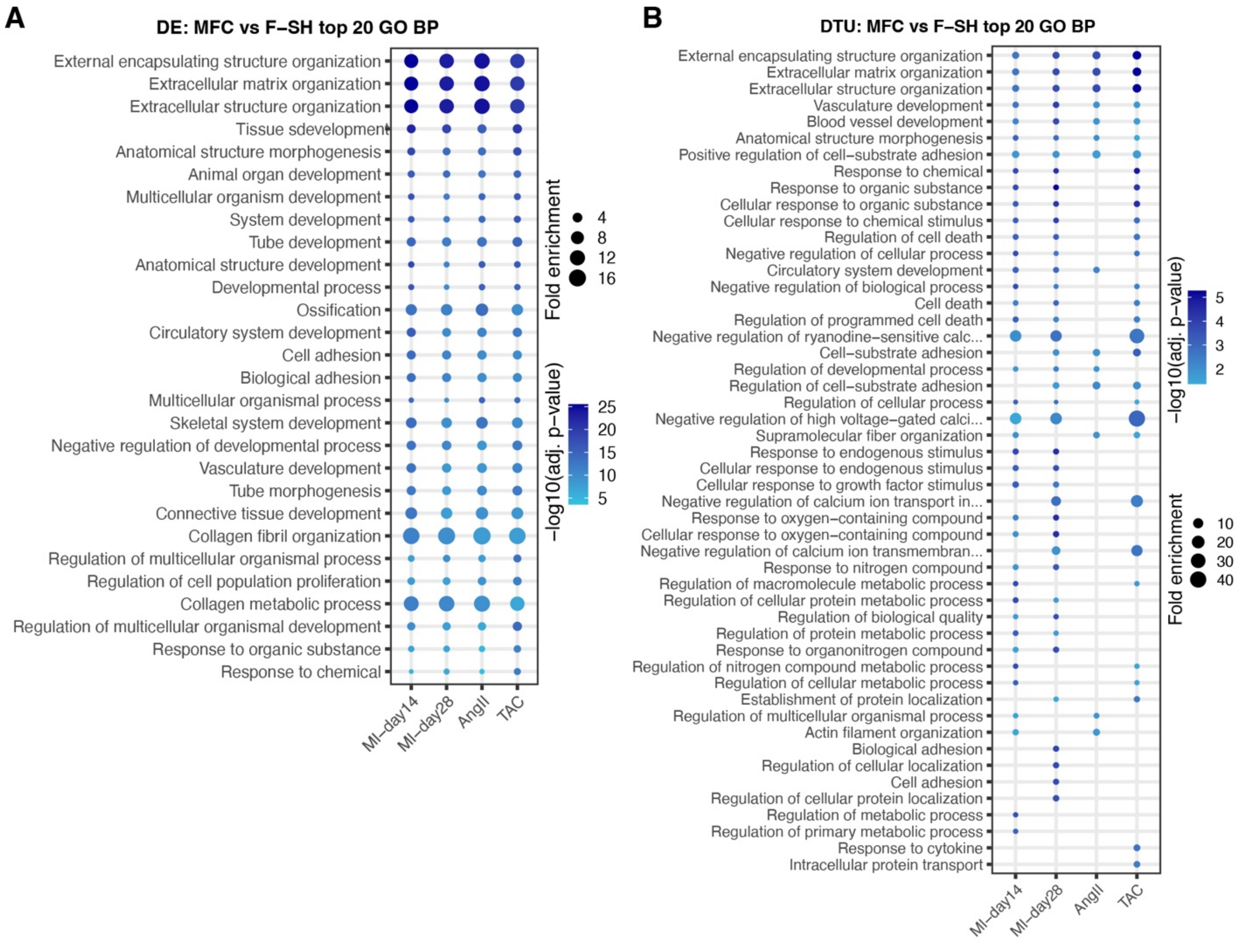

Fig. S10. Differential gene and transcript use in MFC. (A-B) Top 20 GO Biological Process terms for either (A) differentially expressed (DE) genes or (B) differential transcript use (DTU) genes comparing MFC from the indicated disease state/timepoint to F-SH from healthy hearts.

Figure S11

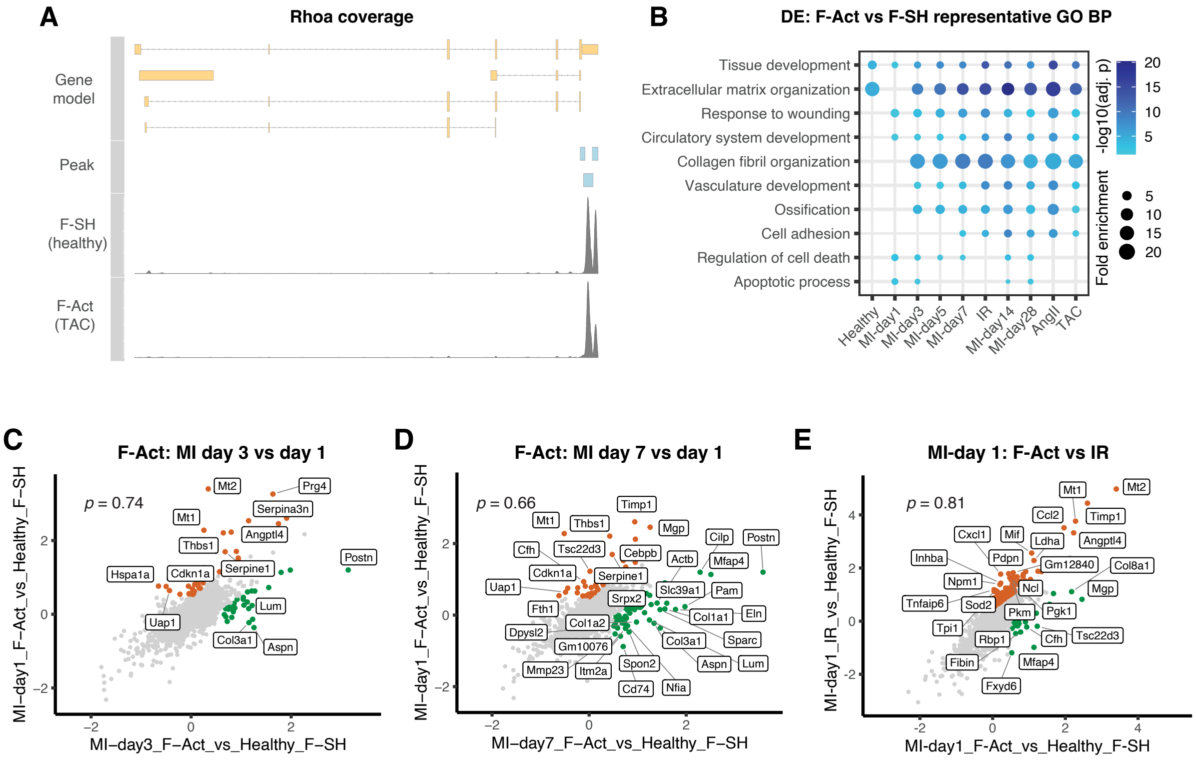

Fig. S11. Analysis of F-Act differential transcript use and gene expression across MI timepoints and disease conditions. (A) Read coverage plot for the *Rhoa* gene, with differential peaks indicated and comparing F-Act coverage from TAC to F-SH expression from the relevant control heart. (B) Representative GO terms comparing F-Act across conditions to F-SH cells from the relevant healthy heart data. (C,D) Comparison of Log_2 fold change differences in gene expression comparing F-Act to F-SH from healthy hearts for (C) F-Act at MI-day 3 vs MI-day 1 or (D) F-Act at MI-day 7 vs MI-day 1. (E) Comparison of Log_2 fold change differences in gene expression comparing F-Act (at MI-day 1) to F-SH against IR vs F-SH.

Figure S12

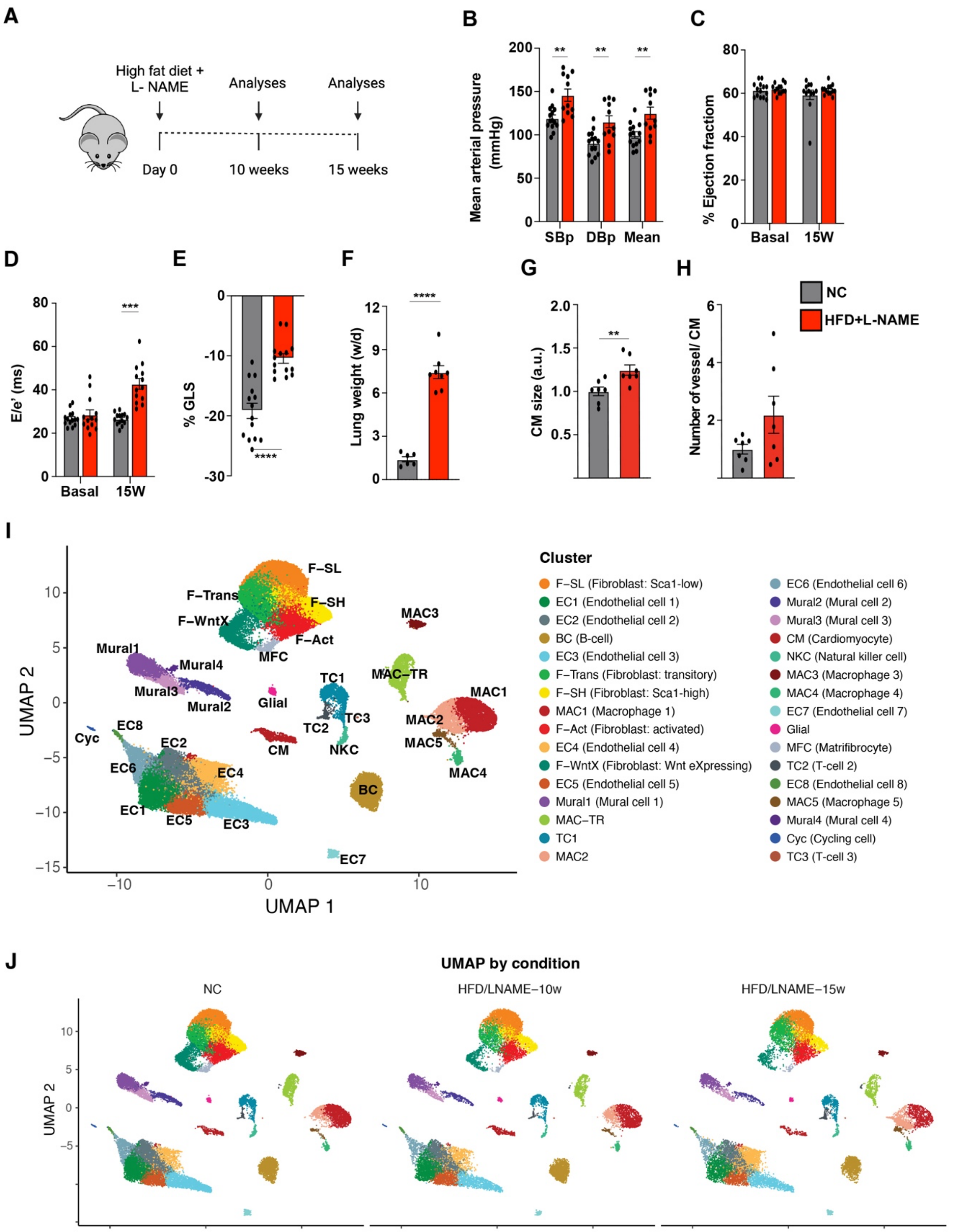
A
High fat diet +
L- NAME
Analyses
Analyses
Day 0
10 weeks
15 weeks
B
Mean arterial pressure (mmHg)
200
150
100
50
0
**
**
**
SBp
DBp
Mean
C
% Ejection fractiom
80
60
40
20
0
Basal
15W
D
E/e' (ms)
80
60
40
20
0
***
Basal
15W
E
% GLS
0
-10
-20
-30
****
F
Lung weight (w/d)
12
9
6
3
0
****
G
CM size (a.u.)
2.0
1.5
1.0
0.5
**
H
Number of vessel/ CM
6
4
2
NC
HFD+L-NAME
I
UMAP 2
UMAP 1
10
5
0
−5
−10
−15
−10
0
10
F−SL
F−Trans
F−SH
F−WntX
F−Act
MFC
MAC3
Mural1
Mural4
Mural3
Mural2
Glial
TC1
MAC−TR
MAC1
TC3
TC2
NKC
MAC2
MAC5
MAC4
CM
Cyc
EC8
EC2
EC6
EC4
EC1
EC5
EC3
BC
EC7
Cluster
F−SL (Fibroblast: Sca1-low)
EC1 (Endothelial cell 1)
EC2 (Endothelial cell 2)
BC (B-cell)
EC3 (Endothelial cell 3)
F−Trans (Fibroblast: transitory)
F−SH (Fibroblast: Sca1-high)
MAC1 (Macrophage 1)
F−Act (Fibroblast: activated)
EC4 (Endothelial cell 4)
F−WntX (Fibroblast: Wnt eXpressing)
EC5 (Endothelial cell 5)
Mural1 (Mural cell 1)
MAC−TR
TC1
MAC2
EC6 (Endothelial cell 6)
Mural2 (Mural cell 2)
Mural3 (Mural cell 3)
CM (Cardiomyocyte)
NKC (Natural killer cell)
MAC3 (Macrophage 3)
MAC4 (Macrophage 4)
EC7 (Endothelial cell 7)
Glial
MFC (Matrifibrocyte)
TC2 (T-cell 2)
EC8 (Endothelial cell 8)
MAC5 (Macrophage 5)
Mural4 (Mural cell 4)
Cyc (Cycling cell)
TC3 (T-cell 3)
J
UMAP by condition
NC
HFD/LNAME−10w
HFD/LNAME−15w
UMAP 2
10
5
0
-5

Fig. S12. Validation and scRNA-seq analysis of HFpEF mice model. (A) Schematic of the experimental design. (B) Systolic (Sbp) and Diastolic blood pressure (Dbp) of experimental groups after fifteen weeks of treatment (n=14 normal chow; n=11 HFD + L-NAME). (C) Percent left ventricular ejection fraction. (D) Ratio of mitral valve E wave to e' wave (E/e'). (E) LV global longitudinal strain (GLS) (B-D n=14 normal chow; n=13 HFD+ L-NAME). (F) Ratio of wet to dry lung weight (LW) (n=6 normal chow; n=8 HFD +L-NAME). (G) Quantification of IF staining shown in Figure S13A for cardiomyocyte area. Values are normalized to normal chow-mice (n=7). (H) Quantification of IF staining shown in Figure S13A for number of capillaries. Values are normalized to normal chow-mice (n=7). (I) Aggregate UMAP showing identified cell types from clustering. (J) UMAP according to condition. *$p < 0.05$, **$p < 0.01$, and ***$p < 0.001$.

Figure S13

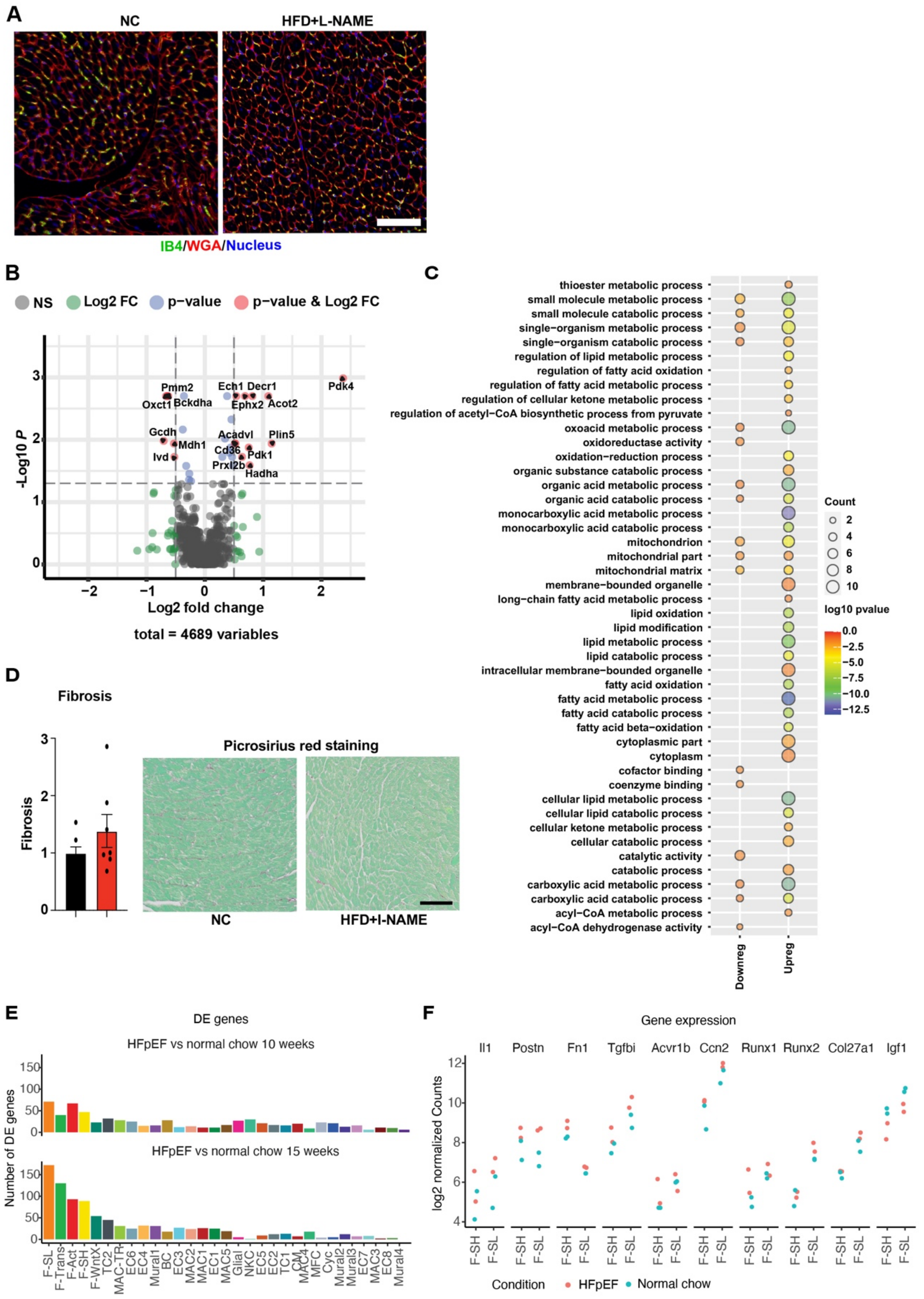
A
NC
HFD+L-NAME
IB4/WGA/Nucleus
B
NS
Log2 FC
p−value
p−value & Log2 FC
-Log10 P
Log2 fold change
total = 4689 variables
Pdk4
Pmm2
Oxct1
Bckdha
Ech1
Decr1
Ephx2
Acot2
Gcdh
Mdh1
Ivd
Acadvl
Cd36
Prxl2b
Pdk1
Plin5
Hadha
C
thioester metabolic process
small molecule metabolic process
small molecule catabolic process
single−organism metabolic process
single−organism catabolic process
regulation of lipid metabolic process
regulation of fatty acid oxidation
regulation of fatty acid metabolic process
regulation of cellular ketone metabolic process
regulation of acetyl−CoA biosynthetic process from pyruvate
oxoacid metabolic process
oxidoreductase activity
oxidation−reduction process
organic substance catabolic process
organic acid metabolic process
organic acid catabolic process
monocarboxylic acid metabolic process
monocarboxylic acid catabolic process
mitochondrion
mitochondrial part
mitochondrial matrix
membrane−bounded organelle
long−chain fatty acid metabolic process
lipid oxidation
lipid modification
lipid metabolic process
lipid catabolic process
intracellular membrane−bounded organelle
fatty acid oxidation
fatty acid metabolic process
fatty acid catabolic process
fatty acid beta−oxidation
cytoplasmic part
cytoplasm
cofactor binding
coenzyme binding
cellular lipid metabolic process
cellular lipid catabolic process
cellular ketone metabolic process
cellular catabolic process
catalytic activity
catabolic process
carboxylic acid metabolic process
carboxylic acid catabolic process
acyl−CoA metabolic process
acyl−CoA dehydrogenase activity
Downreg
Upreg
Count
log10 pvalue
D
Fibrosis
Picrosirius red staining
NC
HFD+l-NAME
E
DE genes
HFpEF vs normal chow 10 weeks
HFpEF vs normal chow 15 weeks
Number of DE genes
F-SL
F-Trans
F-Act
F-SH
F-WntX
TC2
MAC-TR
EC6
EC4
Mural1
BC
EC3
MAC2
MAC1
EC1
MAC5
Glial
NKC
EC5
EC2
TC1
CM
MAC4
MFC
Cyc
Mural2
Mural3
MAC3
EC7
EC8
Mural4
F
Gene expression
Il1
Postn
Fn1
Tgfbi
Acvr1b
Ccn2
Runx1
Runx2
Col27a1
Igf1
log2 normalized Counts
F-SH
F-SL
Condition
HFpEF
Normal chow

Fig. S13. Vascular density, proteomics and fibrosis analysis in the murine HFpEF model. (A) Representative IF images of wheat germ agglutinin (WGA) and isolectin B4 (IB4)-stained heart sections from HFD+L-NAME and normal chow mice. Scale bar, 100 µm. (B) Volcano plot illustrating the distribution of all proteins identified with Tandem Mass Tagged (TMT) discovery proteomics on HFD+L-NAME vs normal chow hearts. (C) Gene Ontology (GO) term analysis on significantly upregulated and downregulated proteins in HFD+L-NAME vs normal chow hearts. (D) Representative images of heart sections showing Picrosirius red and fast green staining. Quantification is shown on the left (n=7). Scale bar, 100 µm. (E) Counts of the number of differentially expressed genes (DESeq2; adj. $p < 0.05$ and Log_2 fold-change > 0.5) between HFpEF and normal chow-fed mice at 10 weeks or 15 weeks of HFD + L-NAME. (F) Expression of select differentially expressed genes. Shown are Log_2 normalised pseudo bulk counts for the biological replicates (n = 2 per condition).

Figure S14

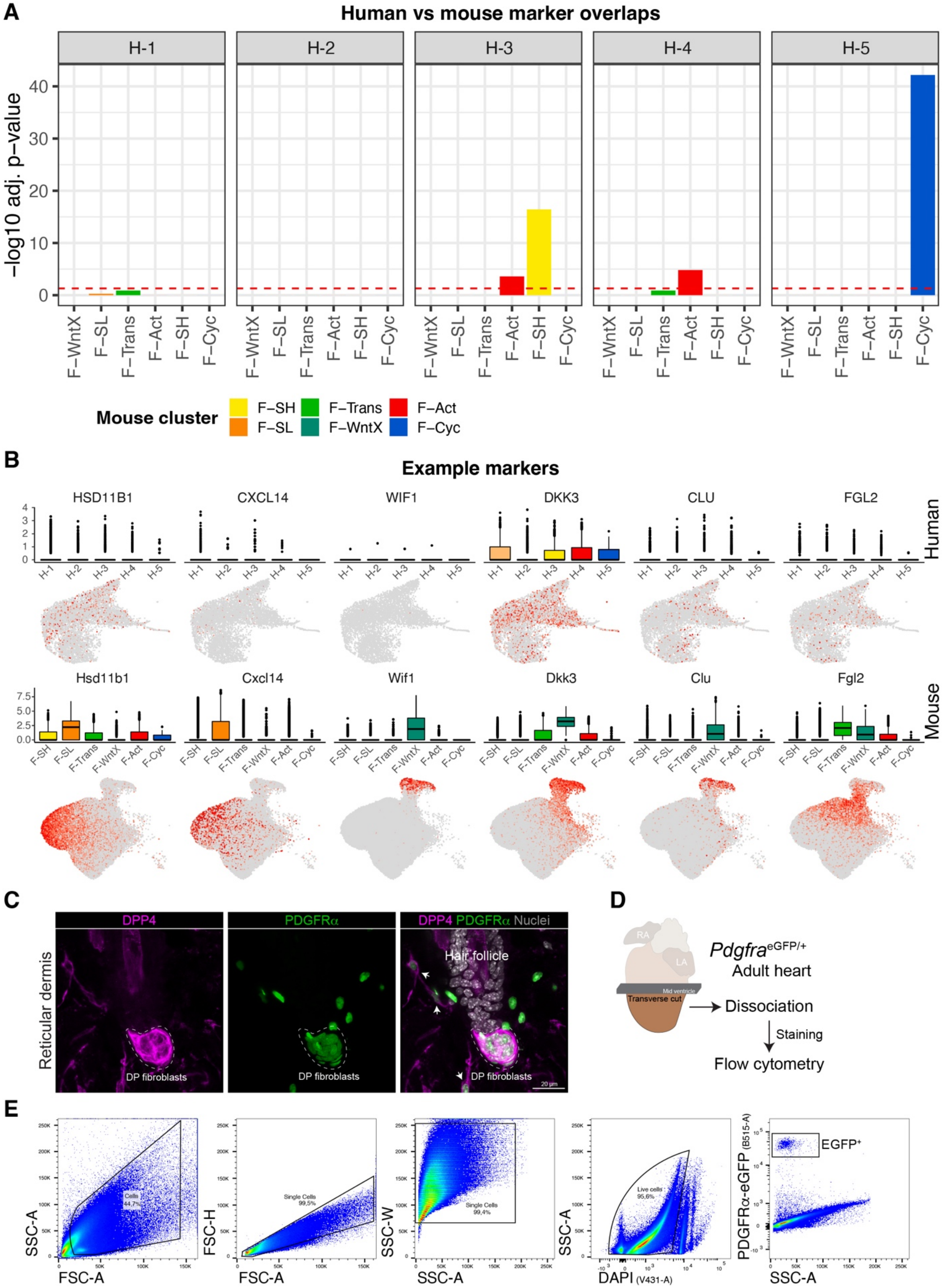

A
Human vs mouse marker overlaps
H-1
H-2
H-3
H-4
H-5
−log10 adj. p−value
0
10
20
30
40
F−WntX
F−SL
F−Trans
F−Act
F−SH
F−Cyc
Mouse cluster
F−SH
F−SL
F−Trans
F−WntX
F−Act
F−Cyc
B
Example markers
HSD11B1
CXCL14
WIF1
DKK3
CLU
FGL2
Human
Hsd11b1
Cxcl14
Wif1
Dkk3
Clu
Fgl2
Mouse
C
DPP4
PDGFRα
DPP4 PDGFRα Nuclei
Reticular dermis
Hair follicle
DP fibroblasts
20 μm
D
RA
LA
Mid ventricle
Transverse cut
Pdgfra eGFP/+
Adult heart
Dissociation
Staining
Flow cytometry
E
SSC-A
FSC-A
Cells 44,7%
FSC-H
Single Cells 99,5%
SSC-W
Single Cells 99,4%
Live cells 95,6%
DAPI (V431-A)
PDGFRα-eGFP (B515-A)
EGFP+

Fig. S14. Comparison of mouse with human fibroblast states. (A) Overlap of marker genes for each of the fibroblast clusters from healthy human hearts (*70*) compared to the fibroblast cell types defined in mouse. Indicated are adjusted *p*-values from Fisher's exact tests. (B) Example marker genes from key mouse fibroblast cell types as visualised in human (top) or mouse (bottom cells in healthy hearts. (C) 24 µm Z-stack high-resolution confocal images showing staining for DPP4 in the reticular dermis and hair follicle niche of adult *Pdgfra*$^{eGFP/+}$ knock-in mice. DP: dermal papilla (n=2). Scale bar, 20 µm. (D) Outline of ventricle isolation, tissue dissociation, antibody staining and later flow cytometry from male *Pdgfra*$^{eGFP/+}$ knock-in mice. (E) Representative flow cytometry and gating strategy of viable eGFP^{+} cardiac fibroblasts obtained from ventricles of male *Pdgfra*$^{eGFP/+}$ knock-in mice. Debris was removed by size and doublets were excluded. Live cells were selected by the absence of DAPI staining (n=3).

Figure S15

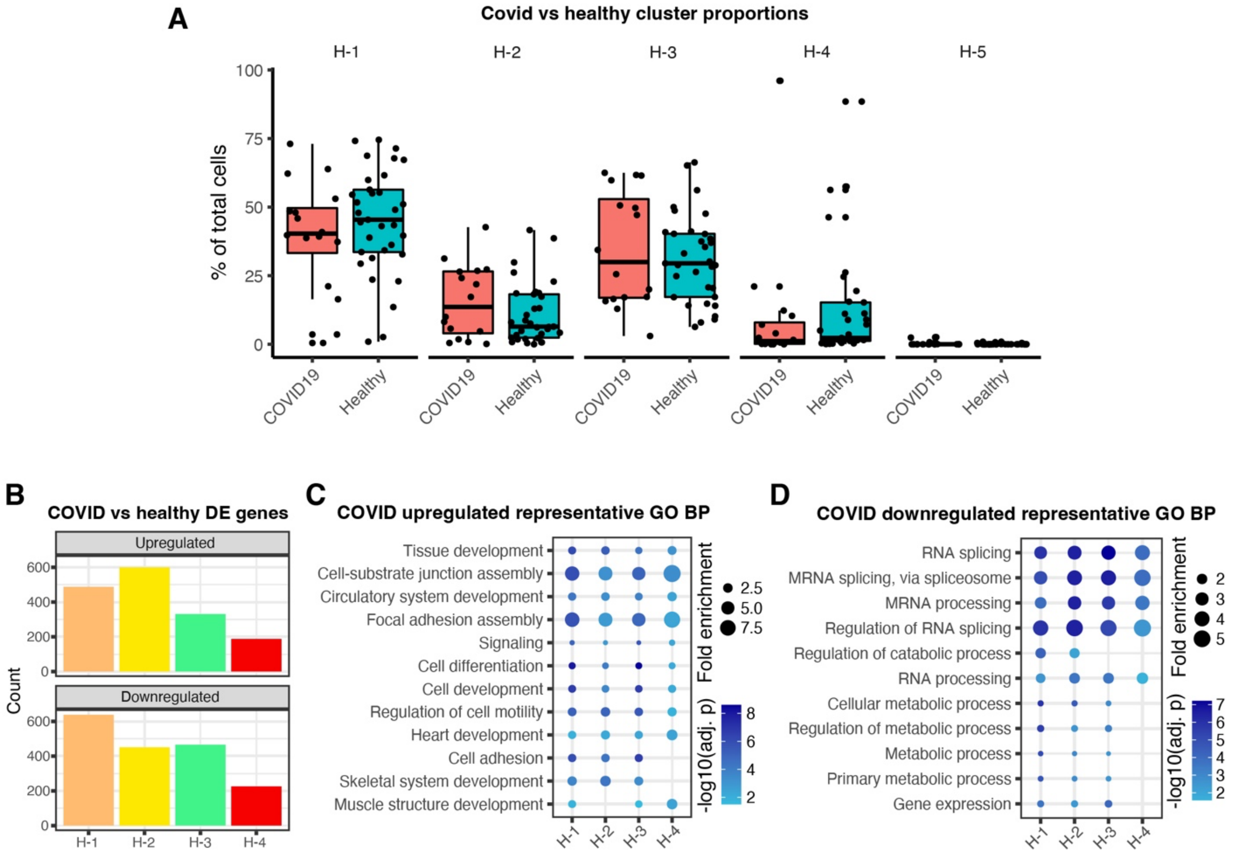

Fig. S15. Analysis of human CF subpopulation in COVID-19 infected versus control hearts. (A) Analysis of percentage of cells from uninfected or COVID-19 infected individuals predicted to correspond to the indicated human fibroblast cell type identified from healthy hearts in Sim and colleagues. (*70*). Shown are the percentage of cells per individual. (B) Counts of up- and downregulated DE genes between COVID and healthy cardiac fibroblasts. (C,D) Representative GO BP terms over-represented among (C) genes upregulated with COVID infection and (D) genes downregulated with COVID infection.

Figure S16

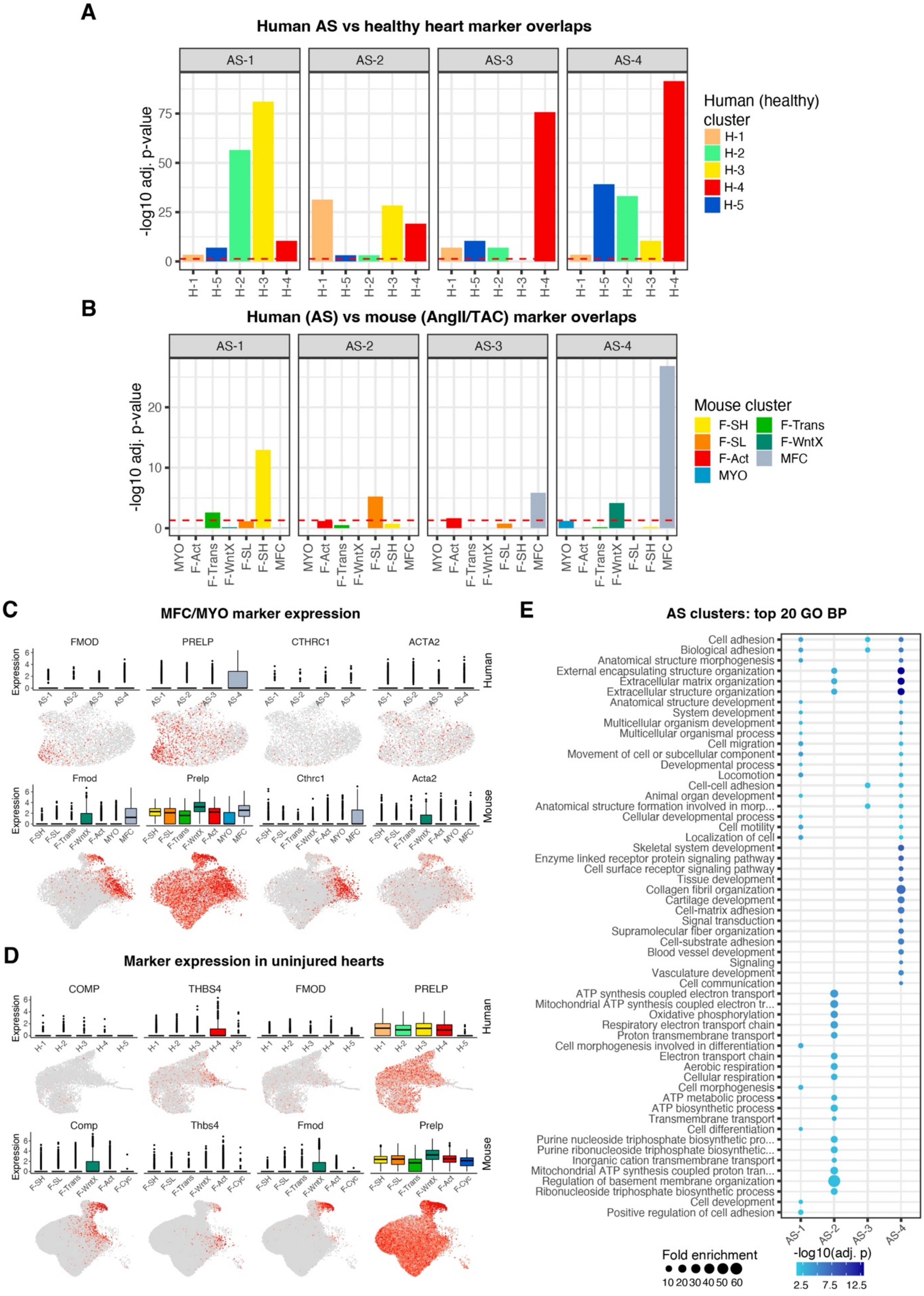
A
Human AS vs healthy heart marker overlaps
AS-1
AS-2
AS-3
AS-4
-log10 adj. p-value
Human (healthy) cluster
H-1
H-2
H-3
H-4
H-5
B
Human (AS) vs mouse (AngII/TAC) marker overlaps
Mouse cluster
F-SH
F-SL
F-Act
MYO
F-Trans
F-WntX
MFC
C
MFC/MYO marker expression
FMOD
PRELP
CTHRC1
ACTA2
Fmod
Prelp
Cthrc1
Acta2
Expression
Human
Mouse
D
Marker expression in uninjured hearts
COMP
THBS4
Comp
Thbs4
F-Cyc
E
AS clusters: top 20 GO BP
Cell adhesion
Biological adhesion
Anatomical structure morphogenesis
External encapsulating structure organization
Extracellular matrix organization
Extracellular structure organization
Anatomical structure development
System development
Multicellular organism development
Multicellular organismal process
Cell migration
Movement of cell or subcellular component
Developmental process
Locomotion
Cell-cell adhesion
Animal organ development
Anatomical structure formation involved in morp...
Cellular developmental process
Cell motility
Localization of cell
Skeletal system development
Enzyme linked receptor protein signaling pathway
Cell surface receptor signaling pathway
Tissue development
Collagen fibril organization
Cartilage development
Cell-matrix adhesion
Signal transduction
Supramolecular fiber organization
Cell-substrate adhesion
Blood vessel development
Signaling
Vasculature development
Cell communication
ATP synthesis coupled electron transport
Mitochondrial ATP synthesis coupled electron tr...
Oxidative phosphorylation
Respiratory electron transport chain
Proton transmembrane transport
Cell morphogenesis involved in differentiation
Electron transport chain
Aerobic respiration
Cellular respiration
Cell morphogenesis
ATP metabolic process
ATP biosynthetic process
Transmembrane transport
Cell differentiation
Purine nucleoside triphosphate biosynthetic pro...
Purine ribonucleoside triphosphate biosynthetic...
Inorganic cation transmembrane transport
Mitochondrial ATP synthesis coupled proton tran...
Regulation of basement membrane organization
Ribonucleoside triphosphate biosynthetic process
Cell development
Positive regulation of cell adhesion
Fold enrichment
10 20 30 40 50 60
-log10(adj. p)
2.5 7.5 12.5

Fig. S16. Comparison of fibroblast states in diseased mouse and human hearts. (A) Overlap of marker genes (MAST testing; $p_{adj} < 1\times10^{-05}$; log2-fold change > 0.5) from the cardiac fibroblast clusters identified in healthy human hearts from Sim and colleagues (*3*) with fibroblast clusters from aortic stenosis (AS) hearts (*22*). Indicated are adjusted *p*-values from Fisher's exact tests. (B) As for (A) but comparing marker genes from murine cardiac fibroblasts from AngII or TAC hearts to human cardiac fibroblasts from AS hearts. (C) Expression of select MYO/MFC markers in (top) diseased human cardiac fibroblasts or (bottom) mouse fibroblasts from hearts of AngII or TAC affected animals. (D) Expression of select markers for MFC or F-WntX in (top) healthy human hearts or (bottom) uninjured mouse hearts. (E) Top 20 GO Biological Process terms over-represented in marker genes for each of the four AS human cardiac fibroblast clusters.

Figure S17

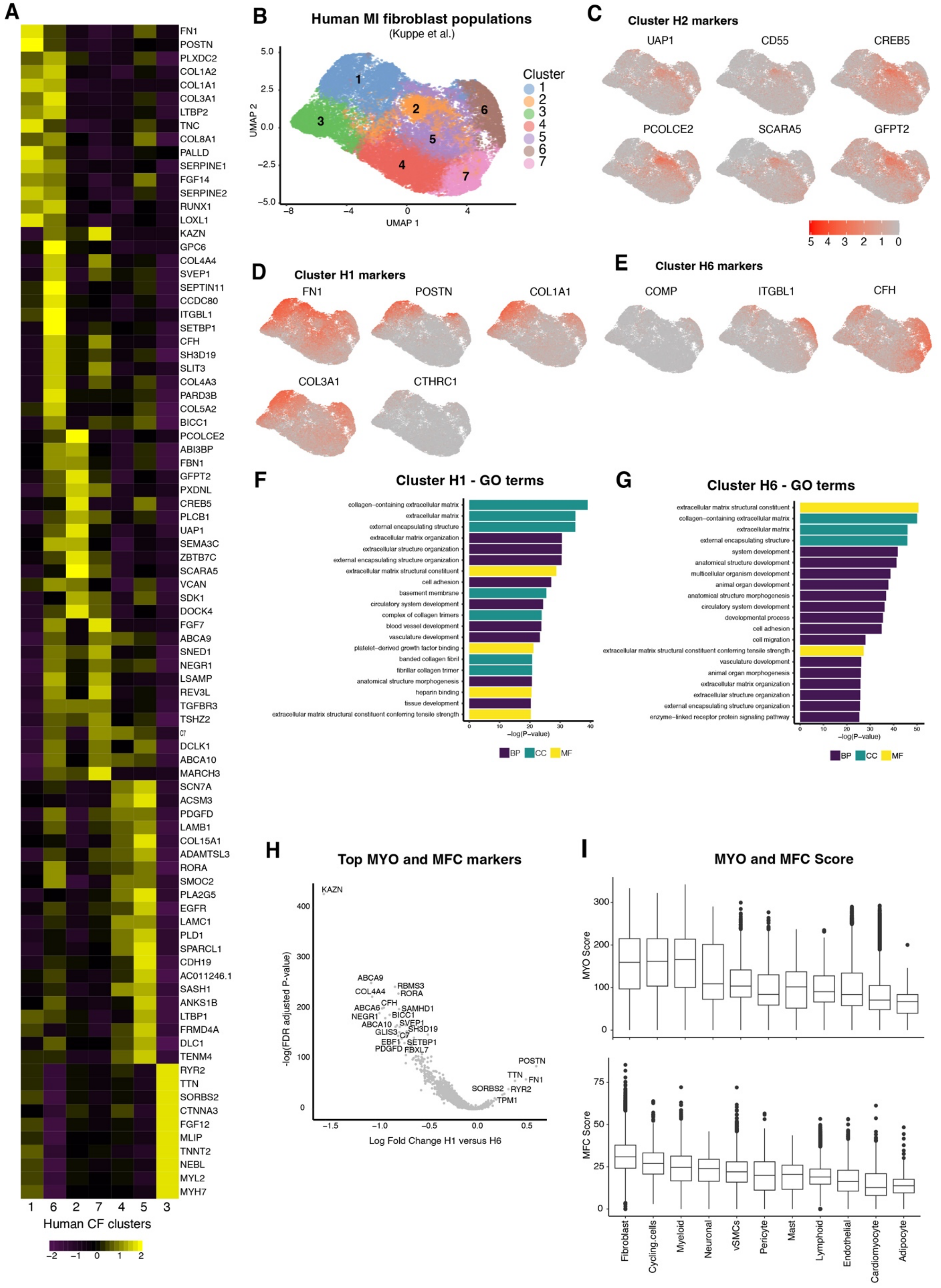
A
Human CF clusters
1 6 2 7 4 5 3
FN1
POSTN
PLXDC2
COL1A2
COL1A1
COL3A1
LTBP2
TNC
COL8A1
PALLD
SERPINE1
FGF14
SERPINE2
RUNX1
LOXL1
KAZN
GPC6
COL4A4
SVEP1
SEPTIN11
CCDC80
ITGBL1
SETBP1
CFH
SH3D19
SLIT3
COL4A3
PARD3B
COL5A2
BICC1
PCOLCE2
ABI3BP
FBN1
GFPT2
PXDNL
CREB5
PLCB1
UAP1
SEMA3C
ZBTB7C
SCARA5
VCAN
SDK1
DOCK4
FGF7
ABCA9
SNED1
NEGR1
LSAMP
REV3L
TGFBR3
TSHZ2
C7
DCLK1
ABCA10
MARCH3
SCN7A
ACSM3
PDGFD
LAMB1
COL15A1
ADAMTSL3
RORA
SMOC2
PLA2G5
EGFR
LAMC1
PLD1
SPARCL1
CDH19
AC011246.1
SASH1
ANKS1B
LTBP1
FRMD4A
DLC1
TENM4
RYR2
TTN
SORBS2
CTNNA3
FGF12
MLIP
TNNT2
NEBL
MYL2
MYH7
−2 −1 0 1 2
B
Human MI fibroblast populations
(Kuppe et al.)
UMAP 2
UMAP 1
Cluster
1
2
3
4
5
6
7
C
Cluster H2 markers
UAP1
CD55
CREB5
PCOLCE2
SCARA5
GFPT2
5 4 3 2 1 0
D
Cluster H1 markers
FN1
POSTN
COL1A1
COL3A1
CTHRC1
E
Cluster H6 markers
COMP
ITGBL1
CFH
F
Cluster H1 - GO terms
collagen−containing extracellular matrix
extracellular matrix
external encapsulating structure
extracellular matrix organization
extracellular structure organization
external encapsulating structure organization
extracellular matrix structural constituent
cell adhesion
basement membrane
circulatory system development
complex of collagen trimers
blood vessel development
vasculature development
platelet−derived growth factor binding
banded collagen fibril
fibrillar collagen trimer
anatomical structure morphogenesis
heparin binding
tissue development
extracellular matrix structural constituent conferring tensile strength
−log(P−value)
BP CC MF
G
Cluster H6 - GO terms
extracellular matrix structural constituent
collagen−containing extracellular matrix
extracellular matrix
external encapsulating structure
system development
anatomical structure development
multicellular organism development
animal organ development
anatomical structure morphogenesis
circulatory system development
developmental process
cell adhesion
cell migration
extracellular matrix structural constituent conferring tensile strength
vasculature development
animal organ morphogenesis
extracellular matrix organization
extracellular structure organization
external encapsulating structure organization
enzyme−linked receptor protein signaling pathway
−log(P−value)
BP CC MF
H
Top MYO and MFC markers
-log(FDR adjusted P-value)
Log Fold Change H1 versus H6
KAZN
ABCA9
RBMS3
RORA
COL4A4
CFH
ABCA6
SAMHD1
NEGR1
BICC1
ABCA10
SVEP1
GLIS3
SH3D19
C7
EBF1
SETBP1
PDGFD
FBXL7
POSTN
TTN
FN1
SORBS2
RYR2
TPM1
I
MYO and MFC Score
MYO Score
MFC Score
Fibroblast
Cycling.cells
Myeloid
Neuronal
vSMCs
Pericyte
Mast
Lymphoid
Endothelial
Cardiomyocyte
Adipocyte

Fig. S17. Analysis of cardiac fibroblasts from human MI hearts. (A) Heatmap of gene expression of human MI fibroblast populations split by unbiased clusters. (B) UMAP of snRNA-seq-resolved human MI fibroblast populations coloured by unbiased clusters. (C) UMAP as in panel A displaying gene expression for selected marker genes for H2. (D) As in panel B for selected marker genes for H1. (E) As in panel B for selected marker genes for H6. (F) Barplot displaying -log(P-value) for the top significantly enriched gene ontology terms associated with cluster H1. (G) As in panel E for cluster H6. (H) Volcano plot displaying log fold change of cluster H1 versus H6 against -log(FDR adjusted P-value). Top genes are labelled on both sides. (I) Boxplots displaying MYO and MFC scores calculated for scRNA-seq-resolved human cells, split by the cell type label. Cell types are sorted according to decreasing mean score for MYO and MFC respectively.

Figure S18

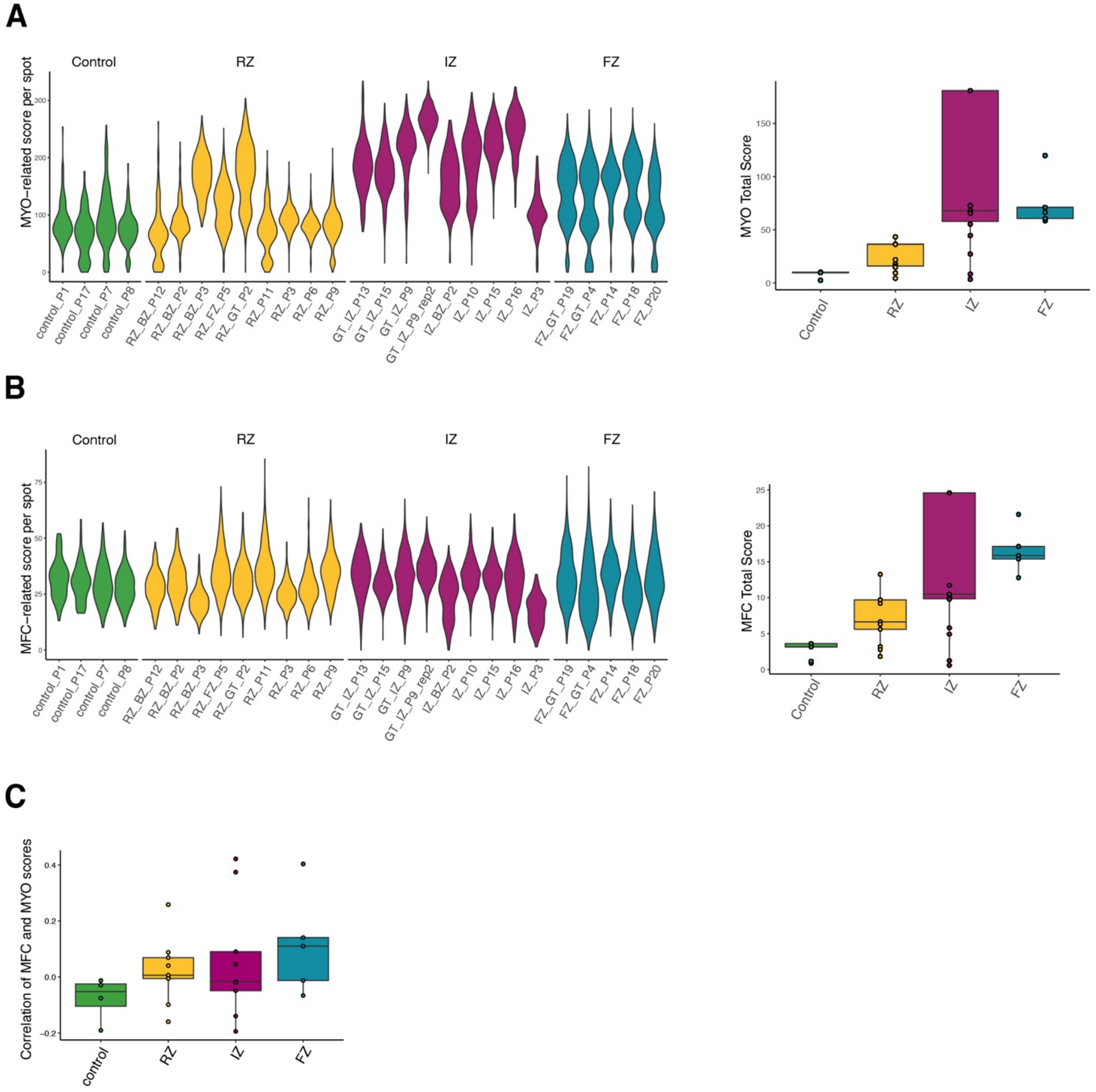

Fig. S18. MYO and MFC scores among clinical samples and heart anatomical regions. (A) Violin plots displaying MYO scores calculated over each Visium-resolved spot, split by patient sample and arranged according to sample type (left), and boxplots displaying total MYO score across entire Visium-resolved sample for each patient arranged by sample type (right). (B) As in panel A for MFC. (C) Boxplots indicating Pearson correlation of MYO and MFC scores for each Visium-resolved spot within each patient sample, arranged by sample type.

Figure S19

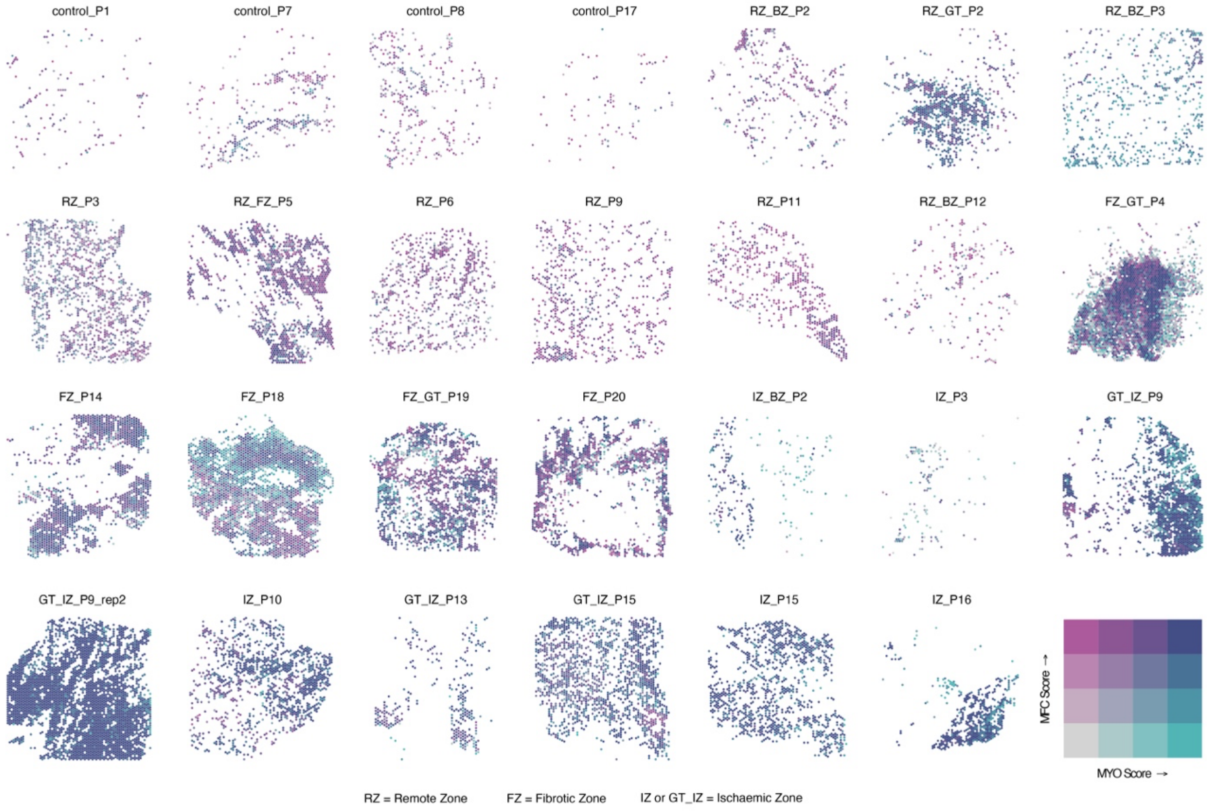

Fig. S19. MYO and MFC scores in clinical samples. Spatial coordinates of Visium-resolved spots in human MI samples, coloured using bivariate colour scheme corresponding to MFC and MYO scores. Only spots corresponding to majority Fibroblast percentage are displayed. Patient samples are arranged according to control, RZ, FZ, and IZ types by row from top to bottom.

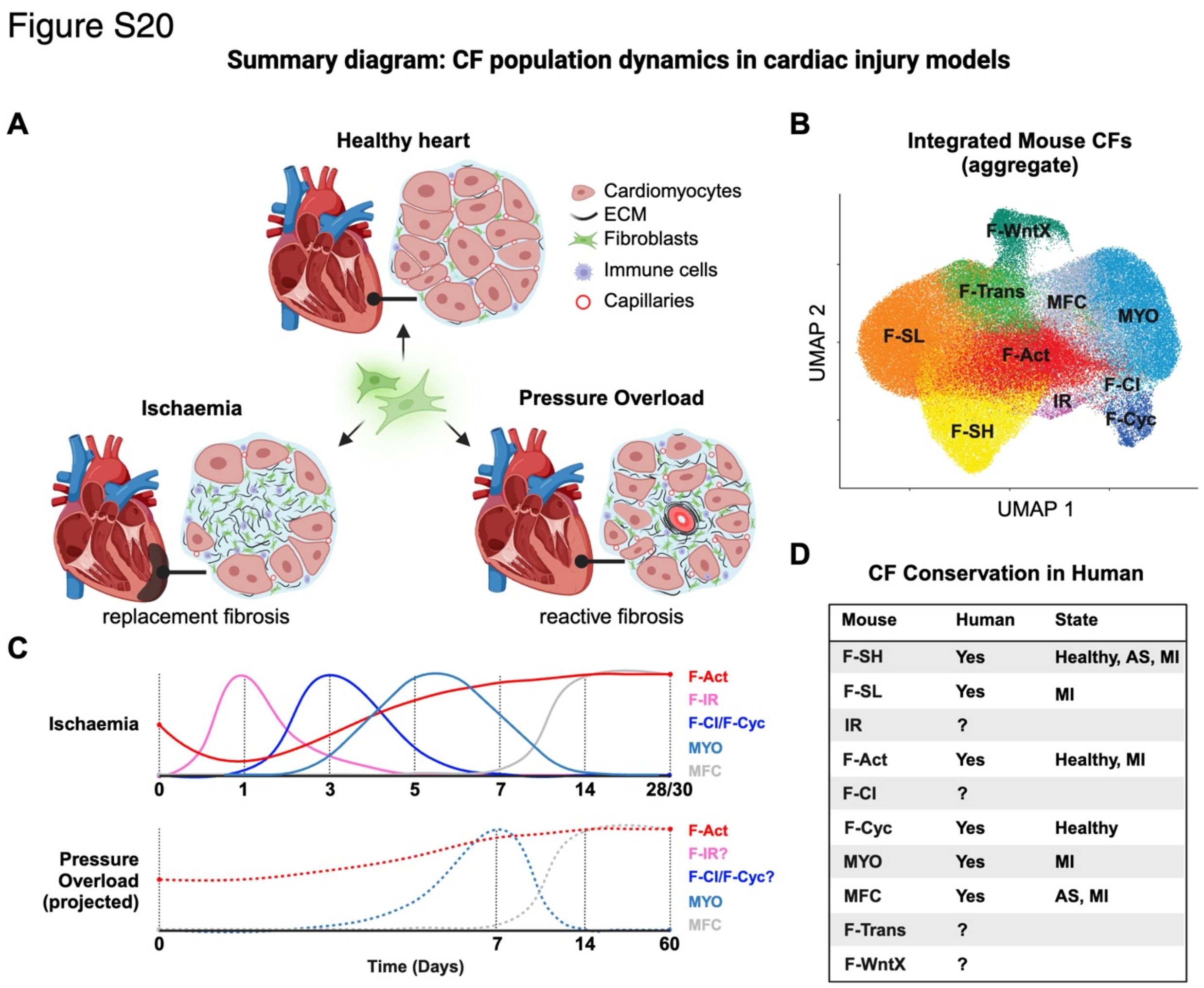

Figure S20. Summary diagram: CF population dynamics in cardiac injury models. (A) Schematic illustrating the two general categories of myocardial fibrosis - replacement fibrosis leading to scarring, as seen in MI and IRI, and reactive (interstitial and perivascular) fibrosis, as found in pressure overload. (B) UMAP plot showing an aggregate of integrated mouse CF data. (C) Projected CF population flux in ischemia versus pressure overload cardiac disease models. Note high homology between specific population involvement and their temporal dynamics. Dotted lines for pressure overload indicate that dynamics have been inferred from limited temporally resolved data. (D) Summary of evidence for conservation of mouse CF states in human hearts investigated in this study.

Supplemental auxiliary files:

Table S1. Differentially expressed genes in CF subsets.

Table S2. Differentially expressed genes between early CF subtypes.

Table S3. Differentially expressed genes within the MYO forming populations.

Table S4. Top 20 inferred transcription factor (TF) networks between identified populations in the integrated MI data.

Table S5. Differentially expressed genes between MYO and MFC and between MYO-1 and MYO-2.

Table S6. Differentially expressed genes between integrated cells from AngII/TAC and MI-days 5-7

Table S7. DE and DTU genes for MFC vs F-SH comparisons across MI-day 14, MI-days 28/30, AngII and TAC.

Table S8. DE and DTU genes for F-Act vs F-SH comparisons

Table S9. Differentially expressed genes and associated GO terms between HFpEF and normal chow mice at week 15 for fibroblast populations.

Table S10. Differentially expressed genes for human fibroblast clusters in healthy and AS hearts plus relative weights assigned to each gene for MYO and MFC identities for calculation of human MYO and MFC scores.

REFERENCES AND NOTES

1. A. Tanay, A. Regev, Scaling single-cell genomics from phenomenology to mechanism. *Nature* **541**, 331–338 (2017).

2. J. E. Rood, A. Maartens, A. Hupalowska, S. A. Teichmann, A. Regev, Impact of the human cell atlas on medicine. *Nat. Med.* **28**, 2486–2496 (2022).

3. J. Lim, V. Chin, K. Fairfax, C. Moutinho, D. Suan, H. Ji, J. E. Powell, Transitioning single-cell genomics into the clinic. *Nat. Rev. Genet.* **24**, 573–584 (2023).

4. N. G. Frangogiannis, Cardiac fibrosis. *Cardiovasc. Res.* **117**, 1450–1488 (2021).

5. J. J. H. Chong, V. Chandrakanthan, M. Xaymardan, N. S. Asli, J. Li, I. Ahmed, C. Heffernan, M. K. Menon, C. J. Scarlett, A. Rashidianfar, C. Biben, H. Zoellner, E. K. Colvin, J. E. Pimanda, A. V. Biankin, B. Zhou, W. T. Pu, O. W. J. Prall, R. P. Harvey, Adult cardiac-resident MSC-like stem cells with a proepicardial origin. *Cell Stem Cell* **9**, 527–540 (2011).

6. H. Soliman, B. Paylor, R. W. Scott, D. R. Lemos, C. K. Chang, M. Arostegui, M. Low, C. Lee, D. Fiore, P. Braghetta, V. Pospichalova, C. E. Barkauskas, V. Korinek, A. Rampazzo, K. MacLeod, T. M. Underhill, F. M. V. Rossi, Pathogenic potential of Hic1-expressing cardiac stromal progenitors. *Cell Stem Cell* **26**, 205–220.e8 (2020).

7. J. T. Kuwabara, A. Hara, S. Bhutada, G. S. Gojanovich, J. Chen, K. Hokutan, V. Shettigar, A. Y. Lee, L. P. DeAngelo, J. R. Heckl, J. R. Jahansooz, D. K. Tacdol, M. T. Ziolo, S. S. Apte, M. D. Tallquist, Consequences of PDGFRα^+ fibroblast reduction in adult murine hearts. *eLife* **11**,e69854 (2022).

8. V. Janbandhu, V. Tallapragada, R. Patrick, Y. Li, D. Abeygunawardena, D. T. Humphreys, E. M. M. A. Martin, A. O. Ward, O. Contreras, N. Farbehi, E. Yao, J. du, S. L. Dunwoodie, N. Bursac, R. P. Harvey, Hif-1a suppresses ROS-induced proliferation of cardiac fibroblasts following myocardial infarction. *Cell Stem Cell* **29**, 281–297.e12 (2022).

9. N. Farbehi, R. Patrick, A. Dorison, M. Xaymardan, V. Janbandhu, K. Wystub-Lis, J. W. K. Ho, R. E. Nordon, R. P. Harvey, Single-cell expression profiling reveals dynamic flux of cardiac stromal, vascular and immune cells in health and injury. *eLife* **8**, e43882 (2019).

10. E. Forte, D. A. Skelly, M. Chen, S. Daigle, K. A. Morelli, O. Hon, V. M. Philip, M. W. Costa, N. A. Rosenthal, M. B. Furtado, Dynamic interstitial cell response during myocardial infarction predicts resilience to rupture in genetically diverse mice. *Cell Rep.* **30**, 3149–3163.e6 (2020).

11. E. A. Rog-Zielinska, R. A. Norris, P. Kohl, R. Markwald, The living scar—Cardiac fibroblasts and the injured heart. *Trends Mol. Med.* **22**, 99–114 (2016).

12. M. D. Tallquist, J. D. Molkentin, Redefining the identity of cardiac fibroblasts. *Nat. Rev. Cardiol.* **14**, 484–491 (2017).

13. M. Pesce, G. N. Duda, G. Forte, H. Girao, A. Raya, P. Roca-Cusachs, J. P. G. Sluijter, C. Tschöpe, S. Van Linthout, Cardiac fibroblasts and mechanosensation in heart development, health and disease. *Nat. Rev. Cardiol.* **20**, 309–324 (2023).

14. H. E. Talbott, S. Mascharak, M. Griffin, D. C. Wan, M. T. Longaker, Wound healing, fibroblast heterogeneity, and fibrosis. *Cell Stem Cell* **29**, 1161–1180 (2022).

15. R. Schuster, J. S. Rockel, M. Kapoor, B. Hinz, The inflammatory speech of fibroblasts. *Immunol. Rev.* **302**, 126–146 (2021).

16. A. González, E. B. Schelbert, J. Diez, J. Butler, Myocardial interstitial fibrosis in heart failure: Biological and translational perspectives. *J. Am. Coll. Cardiol.* **71**, 1696–1706 (2018).

17. M. Walraven, B. Hinz, Therapeutic approaches to control tissue repair and fibrosis: Extracellular matrix as a game changer. *Matrix Biol.* **71-72**, 205–224 (2018).

18. E. Forte, M. Ramialison, H. T. Nim, M. Mara, J. Y. Li, R. Cohn, S. L. Daigle, S. Boyd, E. G. Stanley, A. G. Elefanty, J. T. Hinson, M. W. Costa, N. A. Rosenthal, M. B. Furtado, Adult mouse fibroblasts retain organ-specific transcriptomic identity. *eLife* **11**, e71008 (2022).

19. M. Litviňuková, C. Talavera-López, H. Maatz, D. Reichart, C. L. Worth, E. L. Lindberg, M. Kanda, K. Polanski, M. Heinig, M. Lee, E. R. Nadelmann, K. Roberts, L. Tuck, E. S. Fasouli, D. M. De Laughter, B. M. Donough, H. Wakimoto, J. M. Gorham, S. Samari, K. T. Mahbubani, K. Saeb-Parsy, G. Patone, J. J. Boyle, H. Zhang, H. Zhang, A. Viveiros, G. Y. Oudit, O. A. Bayraktar, J G Seidman, C. E. Seidman, M. Noseda, N. Hubner, S. A. Teichmann, Cells of the adult human heart. *Nature* **588**, 466–472 (2020).

20. M. Chaffin, I. Papangeli, B. Simonson, A. D. Akkad, M. C. Hill, A. Arduini, S. J. Fleming, M. Melanson, S. Hayat, M. Kost-Alimova, O. Atwa, J. Ye, K. C. Bedi Jr., M. Nahrendorf, V. K. Kaushik, C. M. Stegmann, K. B. Margulies, N. R. Tucker, P. T. Ellinor, Single-nucleus profiling of human dilated and hypertrophic cardiomyopathy. *Nature* **608**, 174–180 (2022).

21. C. Kuppe, R. O. Ramirez Flores, Z. Li, S. Hayat, R. T. Levinson, X. Liao, M. T. Hannani, J. Tanevski, F. Wünnemann, J. S. Nagai, M. Halder, D. Schumacher, S. Menzel, G. Schäfer, K. Hoeft, M. Cheng, S. Ziegler, X. Zhang, F. Peisker, N. Kaesler, T. Saritas, Y. Xu, A. Kassner, J. Gummert, M. Morshuis, J. Amrute, R. J. A. Veltrop, P. Boor, K. Klingel, L. W. van Laake, A. Vink, R. M. Hoogenboezem, E. M. J. Bindels, L. Schurgers, S. Sattler, D. Schapiro, R. K. Schneider, K. Lavine, H. Milting, I. G. Costa, J. Saez-Rodriguez, R. Kramann, Spatial multi-omic map of human myocardial infarction. *Nature* **608**, 766–777 (2022).

22. L. Nicin, S. M. Schroeter, S. F. Glaser, R. Schulze-Brüning, M. D. Pham, S. S. Hille, M. Yekelchyk, B. Kattih, W. T. Abplanalp, L. Tombor, O. J. Müller, T. Braun, B. Meder, C. Reich, M. Arsalan, T. Holubec, T. Walther, F. Emrich, J. Krishnan, A. M. Zeiher, D. John, S. Dimmeler, A human cell atlas of the pressure-induced hypertrophic heart. *Nat. Cardiovasc. Res.* **1**, 174–185 (2022).

23. W. T. Abplanalp, N. Tucker, S. Dimmeler, Single-cell technologies to decipher cardiovascular diseases. *Eur. Heart J.* **43**, 4536–4547 (2022).

24. A. M. A. Miranda, V. Janbandhu, H. Maatz, K. Kanemaru, J. Cranley, S. A. Teichmann, N. Hübner, M. D. Schneider, R. P. Harvey, M. Noseda, Single-cell transcriptomics for the assessment of cardiac disease. *Nat. Rev. Cardiol.* **20**, 289–308 (2023).

25. M. Peng, Y. Li, B. Wamsley, Y. Wei, K. Roeder, Integration and transfer learning of single-cell transcriptomes via cFIT. *Proc. Natl. Acad. Sci. U.S.A.* **118**, e2024383118 (2021).

26. T. Stuart, A. Butler, P. Hoffman, C. Hafemeister, E. Papalexi, W. M. Mauck III, Y. Hao, M. Stoeckius, P. Smibert, R. Satija, Comprehensive integration of single-cell data. *Cell* **177**, 1888–1902.e21 (2019).

27. J. F. Ouyang, U. S. Kamaraj, E. Y. Cao, O. J. L. Rackham, ShinyCell: Simple and sharable visualization of single-cell gene expression data. *Bioinformatics* **37**, 3374–3376 (2021).

28. A. Ruiz-Villalba, J. P. Romero, S. C. Hernández, A. Vilas-Zornoza, N. Fortelny, L. Castro-Labrador, P. San Martin-Uriz, E. Lorenzo-Vivas, P. García-Olloqui, M. Palacio, J. J. Gavira, G. Bastarrika, S. Janssens, M. Wu, E. Iglesias, G. Abizanda, X. M. de Morentin, M. Lasaga, N. Planell, C. Bock, D. Alignani, G. Medal, I. Prudovsky, Y. R. Jin, S. Ryzhov, H. Yin, B. Pelacho, D. Gomez-Cabrero, V. Lindner, D. Lara-Astiaso, F. Prósper, Single-cell RNA sequencing analysis reveals a crucial role for CTHRC1 (collagen triple helix repeat containing 1) cardiac fibroblasts after myocardial infarction. *Circulation* **142**, 1831–1847 (2020).

29. I. Korsunsky, N. Millard, J. Fan, K. Slowikowski, F. Zhang, K. Wei, Y. Baglaenko, M. Brenner, P. R. Loh, S. Raychaudhuri, Fast, sensitive and accurate integration of single-cell data with Harmony. *Nat. Methods* **16**, 1289–1296 (2019).

30. Y. Liu, T. Wang, B. Zhou, D. Zheng, Robust integration of multiple single-cell RNA sequencing datasets using a single reference space. *Nat. Biotechnol.* **39**, 877–884 (2021).

31. M. Noseda, M. Harada, S. McSweeney, T. Leja, E. Belian, D. J. Stuckey, M. S. Abreu Paiva, J. Habib, I. Macaulay, A. J. de Smith, F. al-Beidh, R. Sampson, R. T. Lumbers, P. Rao, S. E. Harding, A. I. F. Blakemore, S. Eirik Jacobsen, M. Barahona, M. D. Schneider, PDGFRα demarcates the cardiogenic clonogenic $Sca1^{+}$ stem/progenitor cell in adult murine myocardium. *Nat. Commun.* **6**, 6930 (2015).

32. R. Kramann, R. K. Schneider, D. P. DiRocco, F. Machado, S. Fleig, P. A. Bondzie, J. M. Henderson, B. L. Ebert, B. D. Humphreys, Perivascular $Gli1^{+}$ progenitors are key contributors to injury-induced organ fibrosis. *Cell Stem Cell* **16**, 51–66 (2015).

33. Y. Kfoury, D. T. Scadden, Mesenchymal cell contributions to the stem cell niche. *Cell Stem Cell* **16**, 239–253 (2015).

34. P. Cattaneo, D. Mukherjee, S. Spinozzi, L. Zhang, V. Larcher, W. B. Stallcup, H. Kataoka, J. Chen, S. Dimmeler, S. M. Evans, N. Guimarães-Camboa, Parallel lineage-tracing studies establish fibroblasts as the prevailing in vivo adipocyte progenitor. *Cell Rep.* **30**, 571–582.e2 (2020).

35. O. Contreras, F. M. V. Rossi, M. Theret, Origins, potency, and heterogeneity of skeletal muscle fibro-adipogenic progenitors-time for new definitions. *Skelet. Muscle* **11**, 16 (2021).

36. C. K. F. Chan, P. Lindau, W. Jiang, J. Y. Chen, L. F. Zhang, C.-C. Chen, J. Seita, D. Sahoo, J.-B. Kim, A. Lee, S. Park, D. Nag, Y. Gong, S. Kulkarni, C. A. Luppen, A. A. Theologis, D. C. Wan, A. De Boer, E. Y. Seo, J. D. Vincent-Tompkins, K. Loh, G. G. Walmsley, D. L. Kraft, J. C. Wu, M. T. Longaker, I. L. Weissman, Clonal precursor of bone, cartilage, and hematopoietic niche stromal cells. *Proc. Natl. Acad. Sci. U.S.A.* **110**, 12643–12648 (2013).

37. D. L. Worthley, M. Churchill, J. T. Compton, Y. Tailor, M. Rao, Y. Si, D. Levin, M. G. Schwartz, A. Uygur, Y. Hayakawa, S. Gross, B. W. Renz, W. Setlik, A. N. Martinez, X. Chen, S. Nizami, H. G. Lee, H. P. Kang, J. M. Caldwell, S. Asfaha, C. B. Westphalen, T. Graham, G. Jin, K. Nagar, H. Wang, M. A. Kheirbek, A. Kolhe, J. Carpenter, M. Glaire, A. Nair, S. Renders, N. Manieri, S. Muthupalani, J. G. Fox, M. Reichert, A. S. Giraud, R. F. Schwabe, J. P. Pradere, K. Walton, A. Prakash, D. Gumucio, A. K. Rustgi, T. S. Stappenbeck, R. A. Friedman, M. D. Gershon, P. Sims, T. Grikscheit, F. Y. Lee, G. Karsenty, S. Mukherjee, T. C. Wang, Gremlin 1 identifies a skeletal stem cell with bone, cartilage, and reticular stromal potential. *Cell* **160**, 269–284 (2015).

38. I. C. L. Pillai, S. Li, M. Romay, L. Lam, Y. Lu, J. Huang, N. Dillard, M. Zemanova, L. Rubbi, Y. Wang, J. Lee, M. Xia, O. Liang, Y. H. Xie, M. Pellegrini, A. J. Lusis, A. Deb, Cardiac fibroblasts adopt osteogenic fates and can be targeted to attenuate pathological heart calcification. *Cell Stem Cell* **20**, 218–232.e5 (2017).

39. A. Kohela, E. van Rooij, Fibro-fatty remodelling in arrhythmogenic cardiomyopathy. *Basic Res. Cardiol.* **117**, 22 (2022).

40. L. Muhl, G. Genové, S. Leptidis, J. Liu, L. He, G. Mocci, Y. Sun, S. Gustafsson, B. Buyandelger, I. V. Chivukula, Å. Segerstolpe, E. Raschperger, E. M. Hansson, J. L. M. Björkegren, X. R. Peng, M. Vanlandewijck, U. Lendahl, C. Betsholtz, Single-cell analysis uncovers fibroblast heterogeneity and criteria for fibroblast and mural cell identification and discrimination. *Nat. Commun.* **11**, 3953 (2020).

41. M. Nahrendorf, F. K. Swirski, E. Aikawa, L. Stangenberg, T. Wurdinger, J. L. Figueiredo, P. Libby, R. Weissleder, M. J. Pittet, The healing myocardium sequentially mobilizes two monocyte subsets with divergent and complementary functions. *J. Exp. Med.* **204**, 3037–3047 (2007).

42. G. D. Duerr, D. Dewald, E. J. Schmitz, L. Verfuerth, K. Keppel, C. Peigney, A. Ghanem, A. Welz, O. Dewald, Metallothioneins 1 and 2 modulate inflammation and support remodeling in ischemic cardiomyopathy in mice. *Mediators Inflamm.* **2016**, 7174127 (2016).

43. H. Abe, Y. Tanada, S. Omiya, M. N. Podaru, T. Murakawa, J. Ito, A. M. Shah, S. J. Conway, M. Ono, K. Otsu, NF-κB activation in cardiac fibroblasts results in the recruitment of inflammatory Ly6C^{hi} monocytes in pressure-overloaded hearts. *Sci. Signal.* **14**, eabe4932 (2021).

44. R. Patrick, D. T. Humphreys, V. Janbandhu, A. Oshlack, J. W. K. Ho, R. P. Harvey, K. K. Lo, Sierra: Discovery of differential transcript usage from polyA-captured single-cell RNA-seq data. *Genome Biol.* **21**, 167 (2020).

45. R. Sandberg, J. R. Neilson, A. Sarma, P. A. Sharp, C. B. Burge, Proliferating cells express mRNAs with shortened 3' untranslated regions and fewer microRNA target sites. *Science* **320**, 1643–1647 (2008).

46. X. Fu, H. Khalil, O. Kanisicak, J. G. Boyer, R. J. Vagnozzi, B. D. Maliken, M. A. Sargent, V. Prasad, I. Valiente-Alandi, B. C. Blaxall, J. D. Molkentin, Specialized fibroblast differentiated states underlie scar formation in the infarcted mouse heart. *J. Clin. Invest.* **128**, 2127–2143 (2018).

47. F. Peisker, M. Halder, J. Nagai, S. Ziegler, N. Kaesler, K. Hoeft, R. Li, E. M. J. Bindels, C. Kuppe, J. Moellmann, M. Lehrke, C. Stoppe, M. T. Schaub, R. K. Schneider, I. Costa, R. Kramann, Mapping the cardiac vascular niche in heart failure. *Nat. Commun.* **13**, 3027 (2022).

48. F. A. Wolf, F. K. Hamey, M. Plass, J. Solana, J. S. Dahlin, B. Göttgens, N. Rajewsky, L. Simon, F. J. Theis, PAGA: Graph abstraction reconciles clustering with trajectory inference through a topology preserving map of single cells. *Genome Biol.* **20**, 59 (2019).

49. J. Cao, M. Spielmann, X. Qiu, X. Huang, D. M. Ibrahim, A. J. Hill, F. Zhang, S. Mundlos, L. Christiansen, F. J. Steemers, C. Trapnell, J. Shendure, The single-cell transcriptional landscape of mammalian organogenesis. *Nature* **566**, 496–502 (2019).

50. G. Finak, A. McDavid, M. Yajima, J. Deng, V. Gersuk, A. K. Shalek, C. K. Slichter, H. W. Miller, M. J. McElrath, M. Prlic, P. S. Linsley, R. Gottardo, MAST: A flexible statistical framework for assessing transcriptional changes and characterizing heterogeneity in single-cell RNA sequencing data. *Genome Biol.* **16**, 278 (2015).

51. M. Alexanian, P. F. Przytycki, R. Micheletti, A. Padmanabhan, L. Ye, J. G. Travers, B. Gonzalez-Teran, A. C. Silva, Q. Duan, S. S. Ranade, F. Felix, R. Linares-Saldana, L. Li, C. Y. Lee, N. Sadagopan, A. Pelonero, Y. Huang, G. Andreoletti, R. Jain, T. A. McKinsey, M. G. Rosenfeld, C. A. Gifford, K. S. Pollard, S. M. Haldar, D. Srivastava, A transcriptional switch governs fibroblast activation in heart disease. *Nature* **595**, 438–443 (2021).

52. P. L. Roche, R. S. Nagalingam, R. A. Bagchi, N. Aroutiounova, B. M. J. Belisle, J. T. Wigle, M. P. Czubryt, Role of scleraxis in mechanical stretch-mediated regulation of cardiac myofibroblast phenotype. *Am. J. Physiol. Cell Physiol.* **311**, C297–C307 (2016).

53. P. Badia-I-Mompel, J. V. Santiago, J. Braunger, C. Geiss, D. Dimitrov, S. Müller-Dott, P. Taus, A. Dugourd, C. H. Holland, R. O. R. Flores, J. Saez-Rodriguez, decoupleR: Ensemble of computational methods to infer biological activities from omics data. *Bioinform. Adv.* **2**, vbac016 (2022).

54. W. Ni, A. A. Perez, S. Schreiner, C. M. Nicolet, P. J. Farnham, Characterization of the ZFX family of transcription factors that bind downstream of the start site of CpG island promoters. *Nucleic Acids Res.* **48**, 5986–6000 (2020).

55. X. Chen, H. Xu, P. Yuan, F. Fang, M. Huss, V. B. Vega, E. Wong, Y. L. Orlov, W. Zhang, J. Jiang, Y. H. Loh, H. C. Yeo, Z. X. Yeo, V. Narang, K. R. Govindarajan, B. Leong, A. Shahab, Y. Ruan, G.

Bourque, W. K. Sung, N. D. Clarke, C. L. Wei, H. H. Ng, Integration of external signaling pathways with the core transcriptional network in embryonic stem cells. *Cell* **133**, 1106–1117 (2008).

56. A. Chaves-Sanjuan, N. Gnesutta, A. Gobbini, D. Martignago, A. Bernardini, F. Fornara, R. Mantovani, M. Nardini, Structural determinants for NF-Y subunit organization and NF-Y/DNA association in plants. *Plant J.* **105**, 49–61 (2021).

57. D. Dolfini, R. Gatta, R. Mantovani, NF-Y and the transcriptional activation of CCAAT promoters. *Crit. Rev. Biochem. Mol. Biol.* **47**, 29–49 (2012).

58. N. Ding, R. T. Yu, N. Subramaniam, M. H. Sherman, C. Wilson, R. Rao, M. Leblanc, S. Coulter, M. He, C. Scott, S. L. Lau, A. R. Atkins, G. D. Barish, J. E. Gunton, C. Liddle, M. Downes, R. M. Evans, A vitamin D receptor/SMAD genomic circuit gates hepatic fibrotic response. *Cell* **153**, 601–613 (2013).

59. D. Palioura, A. Lazou, K. Drosatos, Krüppel-like factor (KLF)5: An emerging foe of cardiovascular health. *J. Mol. Cell. Cardiol.* **163**, 56–66 (2022).

60. Q. Zhang, L. Wang, S. Wang, H. Cheng, L. Xu, G. Pei, Y. Wang, C. Fu, Y. Jiang, C. He, Q. Wei, Signaling pathways and targeted therapy for myocardial infarction. *Signal. Transduct. Target. Ther.* **7**, 78 (2022).

61. L. Aryan, D. Younessi, M. Zargari, S. Banerjee, J. Agopian, S. Rahman, R. Borna, G. Ruffenach, S. Umar, M. Eghbali, The role of estrogen receptors in cardiovascular disease. *Int. J. Mol. Sci.* **21**, 4314 (2020).

62. J. Hesse, C. Owenier, T. Lautwein, R. Zalfen, J. F. Weber, Z. Ding, C. Alter, A. Lang, M. Grandoch, N. Gerdes, J. W. Fischer, G. W. Klau, C. Dieterich, K. Köhrer, J. Schrader, Single-cell transcriptomics defines heterogeneity of epicardial cells and fibroblasts within the infarcted murine heart. *eLife* **10**, e65921 (2021).

63. M. A. McLellan, D. A. Skelly, M. S. I. Dona, G. T. Squiers, G. E. Farrugia, T. L. Gaynor, C. D. Cohen, R. Pandey, H. Diep, A. Vinh, N. A. Rosenthal, A. R. Pinto, High-resolution transcriptomic profiling of the heart during chronic stress reveals cellular drivers of cardiac fibrosis and hypertrophy. *Circulation* **142**, 1448–1463 (2020).

64. O. Stenina-Adognravi, E. F. Plow, Thrombospondin-4 in tissue remodeling. *Matrix Biol.* **75-76**, 300–313 (2019).

65. C. L. Zhang, Q. Zhao, H. Liang, X. Qiao, J. Y. Wang, D. Wu, L. L. Wu, L. Li, Cartilage intermediate layer protein-1 alleviates pressure overload-induced cardiac fibrosis via interfering TGF-β1 signaling. *J. Mol. Cell. Cardiol.* **116**, 135–144 (2018).

66. K. Schimmel, K. Ichimura, S. Reddy, F. Haddad, E. Spiekerkoetter, Cardiac fibrosis in the pressure overloaded left and right ventricle as a therapeutic target. *Front. Cardiovasc. Med.* **9**, 886553 (2022).

67. S. R. Ali, S. Ranjbarvaziri, M. Talkhabi, P. Zhao, A. Subat, A. Hojjat, P. Kamran, A. M. S. Müller, K. S. Volz, Z. Tang, K. Red-Horse, R. Ardehali, Developmental heterogeneity of cardiac fibroblasts does not predict pathological proliferation and activation. *Circ. Res.* **115**, 625–635 (2014).

68. H. Khalil, O. Kanisicak, V. Prasad, R. N. Correll, X. Fu, T. Schips, R. J. Vagnozzi, R. Liu, T. Huynh, S. J. Lee, J. Karch, J. D. Molkentin, Fibroblast-specific TGF-β–Smad2/3 signaling underlies cardiac fibrosis. *J. Clin. Invest.* **127**, 3770–3783 (2017).

69. T. Moore-Morris, N. Guimarães-Camboa, I. Banerjee, A. C. Zambon, T. Kisseleva, A. Velayoudon, W. B. Stallcup, Y. Gu, N. D. Dalton, M. Cedenilla, R. Gomez-Amaro, B. Zhou, D. A. Brenner, K. L. Peterson, J. Chen, S. M. Evans, Resident fibroblast lineages mediate pressure overload-induced cardiac fibrosis. *J. Clin. Invest.* **124**, 2921–2934 (2014).

70. R. S. Nagalingam, S. Chattopadhyaya, D. S. al-Hattab, D. Y. C. Cheung, L. Y. Schwartz, S. Jana, N. Aroutiounova, D. A. Ledingham, T. L. Moffatt, N. M. Landry, R. A. Bagchi, I. M. C. Dixon, J. T. Wigle, G. Y. Oudit, Z. Kassiri, D. S. Jassal, M. P. Czubryt, Scleraxis and fibrosis in the pressure-overloaded heart. *Eur. Heart J.* **43**, 4739–4750 (2022).

71. N. Ngwenyama, K. Kaur, D. Bugg, B. Theall, M. Aronovitz, R. Berland, S. Panagiotidou, C. Genco, M. A. Perrin, J. Davis, P. Alcaide, Antigen presentation by cardiac fibroblasts promotes cardiac dysfunction. *Nat. Cardiovasc. Res.* **1**, 761–774 (2022).

72. L. Alex, I. Russo, V. Holoborodko, N. G. Frangogiannis, Characterization of a mouse model of obesity-related fibrotic cardiomyopathy that recapitulates features of human heart failure with preserved ejection fraction. *Am. J. Physiol. Heart Circ. Physiol.* **315**, H934–H949 (2018).

73. D. Mahajan, S. Kancharla, P. Kolli, A. K. Sharma, S. Singh, S. Kumar, A. K. Mohanty, M. K. Jena, Role of fibulins in embryonic stage development and their involvement in various diseases. *Biomolecules* **11**, 685 (2021).

74. T. Sagae, M. Yokogawa, R. Sawazaki, Y. Ishii, N. Hosoda, S. I. Hoshino, S. Imai, I. Shimada, M. Osawa, Paip2A inhibits translation by competitively binding to the RNA recognition motifs of PABPC1 and promoting its dissociation from the poly(A) tail. *J. Biol. Chem.* **298**, 101844 (2022).

75. S. Chorghade, J. Seimetz, R. Emmons, J. Yang, S. M. Bresson, M. D. Lisio, G. Parise, N. K. Conrad, A. Kalsotra, Poly(A) tail length regulates PABPC1 expression to tune translation in the heart. *eLife* **6**, e24139 (2017).

76. L. S. Kilian, J. Voran, D. Frank, A. Y. Rangrez, RhoA: A dubious molecule in cardiac pathophysiology. *J. Biomed. Sci.* **28**, 33 (2021).

77. J. Roh, J. A. Hill, A. Singh, M. Valero-Munoz, F. Sam, Heart failure with preserved ejection fraction: Heterogeneous syndrome, diverse preclinical models. *Circ. Res.* **130**, 1906–1925 (2022).

78. G. G. Schiattarella, F. Altamirano, D. Tong, K. M. French, E. Villalobos, S. Y. Kim, X. Luo, N. Jiang, H. I. May, Z. V. Wang, T. M. Hill, P. P. A. Mammen, J. Huang, D. I. Lee, V. S. Hahn, K. Sharma, D. A. Kass, S. Lavandero, T. G. Gillette, J. A. Hill, Nitrosative stress drives heart failure with preserved ejection fraction. *Nature* **568**, 351–356 (2019).

79. G. G. Schiattarella, F. Altamirano, S. Y. Kim, D. Tong, A. Ferdous, H. Piristine, S. Dasgupta, X. Wang, K. M. French, E. Villalobos, S. B. Spurgin, M. Waldman, N. Jiang, H. I. May, T. M. Hill, Y. Luo, H. Yoo, V. G. Zaha, S. Lavandero, T. G. Gillette, J. A. Hill, Xbp1s-FoxO1 axis governs lipid accumulation and contractile performance in heart failure with preserved ejection fraction. *Nat. Commun.* **12**, 1684 (2021).

80. C. B. Sim, B. Phipson, M. Ziemann, H. Rafehi, R. J. Mills, K. I. Watt, K. D. Abu-Bonsrah, R. K. R. Kalathur, H. K. Voges, D. T. Dinh, M. ter Huurne, C. J. Vivien, A. Kaspi, H. Kaipananickal, A. Hidalgo, L. M. D. Delbridge, R. L. Robker, P. Gregorevic, C. G. dos Remedios, S. Lal, A. T. Piers, I. E. Konstantinov, D. A. Elliott, A. el-Osta, A. Oshlack, J. E. Hudson, E. R. Porrello, Sex-specific control of human heart maturation by the progesterone receptor. *Circulation* **143**, 1614–1628 (2021).

81. L. Wang, Y. Yang, H. Ma, Y. Xie, J. Xu, D. Near, H. Wang, T. Garbutt, Y. Li, J. Liu, L. Qian, Single cell dual-omics reveals the transcriptomic and epigenomic diversity of cardiac non-myocytes. *Cardiovasc. Res.* **118**, 1548–1563 (2022).

82. M. B. Buechler, R. N. Pradhan, A. T. Krishnamurty, C. Cox, A. K. Calviello, A. W. Wang, Y. A. Yang, L. Tam, R. Caothien, M. Roose-Girma, Z. Modrusan, J. R. Arron, R. Bourgon, S. Müller, S. J. Turley, Cross-tissue organization of the fibroblast lineage. *Nature* **593**, 575–579 (2021).

83. D. Merrick, A. Sakers, Z. Irgebay, C. Okada, C. Calvert, M. P. Morley, I. Percec, P. Seale, Identification of a mesenchymal progenitor cell hierarchy in adipose tissue. *Science* **364**, eaav2501 (2019).

84. R. R. Driskell, F. M. Watt, Understanding fibroblast heterogeneity in the skin. *Trends Cell Biol.* **25**, 92–99 (2015).

85. W. Haussner, A. P. DeRosa, D. Haussner, J. Tran, J. Torres-Lavoro, J. Kamler, K. Shah, COVID-19 associated myocarditis: A systematic review. *Am. J. Emerg. Med.* **51**, 150–155 (2022).

86. M. I. Brener, M. L. Hulke, N. Fukuma, S. Golob, R. S. Zilinyi, Z. Zhou, C. Tzimas, I. Russo, C. McGroder, R. D. Pfeiffer, A. Chong, G. Zhang, D. Burkhoff, M. B. Leon, M. S. Maurer, J. W. Moses, A. C. Uhlemann, H. Hibshoosh, N. Uriel, M. J. Szabolcs, B. Redfors, C. C. Marboe, M. R. Baldwin, N. R. Tucker, E. J. Tsai, Clinico-histopathologic and single-nuclei RNA-sequencing insights into cardiac injury and microthrombi in critical COVID-19. *JCI Insight* **7**, e154633 (2022).

87. X. Zou, K. Chen, J. Zou, P. Han, J. Hao, Z. Han, Single-cell RNA-seq data analysis on the receptor ACE2 expression reveals the potential risk of different human organs vulnerable to 2019-nCoV infection. *Front. Med.* **14**, 185–192 (2020).

88. T. M. Delorey, C. G. K. Ziegler, G. Heimberg, R. Normand, Y. Yang, Å. Segerstolpe, D. Abbondanza, S. J. Fleming, A. Subramanian, D. T. Montoro, K. A. Jagadeesh, K. K. Dey, P. Sen, M. Slyper, Y. H. Pita-Juárez, D. Phillips, J. Biermann, Z. Bloom-Ackermann, N. Barkas, A. Ganna, J. Gomez, J. C. Melms, I. Katsyv, E. Normandin, P. Naderi, Y. V. Popov, S. S. Raju, S. Niezen, L. T. Y. Tsai, K. J. Siddle, M. Sud, V. M. Tran, S. K. Vellarikkal, Y. Wang, L. Amir-Zilberstein, D. S. Atri, J. Beechem, O. R. Brook, J. Chen, P. Divakar, P. Dorceus, J. M. Engreitz, A. Essene, D. M. Fitzgerald, R. Fropf, S. Gazal, J. Gould, J. Grzyb, T. Harvey, J. Hecht, T. Hether, J. Jané-Valbuena, M. Leney-Greene, H. Ma, C. McCabe, D. E. McLoughlin, E. M. Miller, C. Muus, M. Niemi, R. Padera, L. Pan, D. Pant, C. Pe'er, J. Pfiffner-Borges, C. J. Pinto, J. Plaisted, J. Reeves, M. Ross, M. Rudy, E. H. Rueckert, M. Siciliano, A. Sturm, E. Todres, A. Waghray, S. Warren, S. Zhang, D. R. Zollinger, L. Cosimi, R. M. Gupta, N. Hacohen, H. Hibshoosh, W. Hide, A. L. Price, J. Rajagopal, P. R. Tata, S. Riedel, G. Szabo, T. L. Tickle, P. T. Ellinor, D. Hung, P. C. Sabeti, R. Novak, R. Rogers, D. E. Ingber, Z. G. Jiang, D. Juric, M. Babadi, S. L. Farhi, B. Izar, J. R. Stone, I. S. Vlachos, I. H. Solomon, O. Ashenberg, C. B. M. Porter, B. Li, A. K. Shalek, A. C. Villani, O. Rozenblatt-Rosen, A. Regev, COVID-19 tissue atlases reveal SARS-CoV-2 pathology and cellular targets. *Nature* **595**, 107–113 (2021).

89. C. Wang, L. Chen, Y. Chen, W. Jia, X. Cai, Y. Liu, F. Ji, P. Xiong, A. Liang, R. Liu, Y. Guan, Z. Cheng, Y. Weng, W. Wang, Y. Duan, D. Kuang, S. Xu, H. Cai, Q. Xia, D. Yang, M. W. Wang, X. Yang, J. Zhang, C. Cheng, L. Liu, Z. Liu, R. Liang, G. Wang, Z. Li, H. Xia, T. Xia, Abnormal global alternative RNA splicing in COVID-19 patients. *PLOS Genet.* **18**, e1010137 (2022).

90. A. K. Banerjee, M. R. Blanco, E. A. Bruce, D. D. Honson, L. M. Chen, A. Chow, P. Bhat, N. Ollikainen, S. A. Quinodoz, C. Loney, J. Thai, Z. D. Miller, A. E. Lin, M. M. Schmidt, D. G. Stewart, D. Goldfarb, G. de Lorenzo, S. J. Rihn, R. M. Voorhees, J. W. Botten, D. Majumdar, M. Guttman, SARS-CoV-2 disrupts splicing, translation, and protein trafficking to suppress host defenses. *Cell* **183**, 1325–1339.e21 (2020).

91. L. Zhuang, L. Lu, R. Zhang, K. Chen, X. Yan, Comprehensive integration of single-cell transcriptional profiling reveals the heterogeneities of non-cardiomyocytes in healthy and ischemic hearts. *Front. Cardiovasc. Med.* **7**, 615161 (2020).

92. K. Kanemaru, J. Cranley, D. Muraro, A. M. A. Miranda, S. Y. Ho, A. Wilbrey-Clark, J. Patrick Pett, K. Polanski, L. Richardson, M. Litvinukova, N. Kumasaka, Y. Qin, Z. Jablonska, C. I. Semprich, L. Mach, M. Dabrowska, N. Richoz, L. Bolt, L. Mamanova, R. Kapuge, S. N. Barnett, S. Perera, C. Talavera-López, I. Mulas, K. T. Mahbubani, L. Tuck, L. Wang, M. M. Huang, M. Prete, S. Pritchard, J. Dark, K. Saeb-Parsy, M. Patel, M. R. Clatworthy, N. Hübner, R. A. Chowdhury, M. Noseda, S. A. Teichmann, Spatially resolved multiomics of human cardiac niches. *Nature* **619**, 801–810 (2023).

93. K. M. Sitnik, K. Wendland, H. Weishaupt, H. Uronen-Hansson, A. J. White, G. Anderson, K. Kotarsky, W. W. Agace, Context-dependent development of lymphoid stroma from adult $CD34^+$ adventitial progenitors. *Cell Rep.* **14**, 2375–2388 (2016).

94. K. R. Stenmark, M. E. Yeager, K. C. el Kasmi, E. Nozik-Grayck, E. V. Gerasimovskaya, M. Li, S. R. Riddle, M. G. Frid, The adventitia: Essential regulator of vascular wall structure and function. *Annu. Rev. Physiol.* **75**, 23–47 (2013).

95. M. Corselli, C. W. Chen, B. Sun, S. Yap, J. P. Rubin, B. Péault, The tunica adventitia of human arteries and veins as a source of mesenchymal stem cells. *Stem Cells Dev.* **21**, 1299–1308 (2012).

96. M. W. Dahlgren, S. W. Jones, K. M. Cautivo, A. Dubinin, J. F. Ortiz-Carpena, S. Farhat, K. S. Yu, K. Lee, C. Wang, A. V. Molofsky, A. D. Tward, M. F. Krummel, T. Peng, A. B. Molofsky, Adventitial stromal cells define group 2 innate lymphoid cell tissue niches. *Immunity* **50**, 707–722.e6 (2019).

97. D. Park, J. A. Spencer, B. I. Koh, T. Kobayashi, J. Fujisaki, T. L. Clemens, C. P. Lin, H. M. Kronenberg, D. T. Scadden, Endogenous bone marrow MSCs are dynamic, fate-restricted participants in bone maintenance and regeneration. *Cell Stem Cell* **10**, 259–272 (2012).

98. V. W. Yu, D. T. Scadden, Heterogeneity of the bone marrow niche. *Curr. Opin. Hematol.* **23**, 331–338 (2016).

99. W. Gu, Z. Ni, Y. Q. Tan, J. Deng, S. J. Zhang, Z. C. Lv, X. J. Wang, T. Chen, Z. Zhang, Y. Hu, Z. C. Jing, Q. Xu, Adventitial cell atlas of wt (wild type) and ApoE (apolipoprotein E)-deficient mice defined by single-cell RNA sequencing. *Arterioscler. Thromb. Vasc. Biol.* **39**, 1055–1071 (2019).

100. G. Díaz-Araya, R. Vivar, C. Humeres, P. Boza, S. Bolivar, C. Muñoz, Cardiac fibroblasts as sentinel cells in cardiac tissue: Receptors, signaling pathways and cellular functions. *Pharmacol. Res.* **101**, 30–40 (2015).

101. J. T. Rodgers, M. D. Schroeder, C. Ma, T. A. Rando, HGFA is an injury-regulated systemic factor that induces the transition of stem cells into GAlert. *Cell Rep.* **19**, 479–486 (2017).

102. N. G. Frangogiannis, The inflammatory response in myocardial injury, repair, and remodelling. *Nat. Rev. Cardiol.* **11**, 255–265 (2014).

103. M. J. Ivey, J. T. Kuwabara, J. T. Pai, R. E. Moore, Z. Sun, M. D. Tallquist, Resident fibroblast expansion during cardiac growth and remodeling. *J. Mol. Cell. Cardiol.* **114**, 161–174 (2018).

104. D. Jeong, M. A. Lee, Y. Li, D. K. Yang, C. Kho, J. G. Oh, G. Hong, A. Lee, M. H. Song, T. J. LaRocca, J. Chen, L. Liang, S. Mitsuyama, V. D'Escamard, J. C. Kovacic, T. H. Kwak, R. J. Hajjar, W. J. Park, Matricellular protein CCN5 reverses established cardiac fibrosis. *J. Am. Coll. Cardiol.* **67**, 1556–1568 (2016).

105. A. Hillsley, M. S. Santoso, S. M. Engels, K. N. Halwachs, L. M. Contreras, A. M. Rosales, A strategy to quantify myofibroblast activation on a continuous spectrum. *Sci. Rep.* **12**, 12239 (2022).

106. T. B. Layton, L. Williams, H. Colin-York, F. E. McCann, M. Cabrita, M. Feldmann, C. Brown, W. Xie, M. Fritzsche, D. Furniss, J. Nanchahal, Single cell force profiling of human myofibroblasts reveals a biophysical spectrum of cell states. *Biol. Open* **9**, bio049809 (2020).

107. C. Humeres, A. V. Shinde, A. Hanna, L. Alex, S. C. Hernández, R. Li, B. Chen, S. J. Conway, N. G. Frangogiannis, Smad7 effects on TGF-β and ErbB2 restrain myofibroblast activation and protect from postinfarction heart failure. *J. Clin. Invest.* **132**, e146926 (2022).

108. R. Vivar, C. Humeres, R. Anfossi, S. Bolivar, M. Catalán, J. Hill, S. Lavandero, G. Diaz-Araya, Role of FoxO3a as a negative regulator of the cardiac myofibroblast conversion induced by TGF-β1. *Biochim. Biophys. Acta Mol. Cell Res.* **1867**, 118695 (2020).

109. B. Hinz, D. Lagares, Evasion of apoptosis by myofibroblasts: A hallmark of fibrotic diseases. *Nat. Rev. Rheumatol.* **16**, 11–31 (2020).

110. J. D. Lanzer, L. M. Wienecke, R. O. Ramirez-Flores, M. M. Zylla, N. Hartmann, F. Sicklinger, J.-H. Schultz, N. Frey, J. Saez-Rodriguez, F. Leuschner, Single-cell transcriptomics reveal distinctive patterns of fibroblast activation in murine heart failure with preserved ejection fraction. bioRxiv 2023.05.09.539983 [Preprint] (2023). https://doi.org/10.1101/2023.05.09.539983.

111. D. Tong, G. G. Schiattarella, N. Jiang, F. Altamirano, P. A. Szweda, A. Elnwasany, D. I. Lee, H. Yoo, D. A. Kass, L. I. Szweda, S. Lavandero, E. Verdin, T. G. Gillette, J. A. Hill, NAD^+ repletion reverses heart failure with preserved ejection fraction. *Circ. Res.* **128**, 1629–1641 (2021).

112. M. Abdellatif, V. Trummer-Herbst, F. Koser, S. Durand, R. Adão, F. Vasques-Nóvoa, J. K. Freundt, J. Voglhuber, M. R. Pricolo, M. Kasa, C. Türk, F. Aprahamian, E. Herrero-Galán, S. J. Hofer, T. Pendl, L. Rech, J. Kargl, N. Anto-Michel, S. Ljubojevic-Holzer, J. Schipke, C. Brandenberger, M. Auer, R. Schreiber, C. N. Koyani, A. Heinemann, A. Zirlik, A. Schmidt, D. von Lewinski, D. Scherr, P. P. Rainer, J. von Maltzahn, C. Mühlfeld, M. Krüger, S. Frank, F. Madeo, T. Eisenberg, A. Prokesch, A. F. Leite-Moreira, A. P. Lourenço, J. Alegre-Cebollada, S. Kiechl, W. A. Linke, G. Kroemer, S. Sedej, Nicotinamide for the treatment of heart failure with preserved ejection fraction. *Sci. Transl. Med.* **13**, eabd7064 (2021).

113. E. Boileau, X. Li, I. S. Naarmann-de Vries, C. Becker, R. Casper, J. Altmüller, F. Leuschner, C. Dieterich, Full-length spatial transcriptomics reveals the unexplored isoform diversity of the myocardium post-MI. *Front. Genet.* **13**, 912572 (2022).

114. S. L. Wolock, R. Lopez, A. M. Klein, Scrublet: Computational identification of cell doublets in single-cell transcriptomic data. *Cell Syst.* **8**, 281–291.e9 (2019).

115. Z. Gu, R. Eils, M. Schlesner, Complex heatmaps reveal patterns and correlations in multidimensional genomic data. *Bioinformatics* **32**, 2847–2849 (2016).

116. M. N. Wright, A. Ziegler, ranger: A fast implementation of random forests for high dimensional data in C++ and R. *J. Stat. Softw.* **77**, 1–17 (2017).

117. F. A. Wolf, P. Angerer, F. J. Theis, SCANPY: Large-scale single-cell gene expression data analysis. *Genome Biol.* **19**, 15 (2018).

118. M. I. Love, W. Huber, S. Anders, Moderated estimation of fold change and dispersion for RNA-seq data with DESeq2. *Genome Biol.* **15**, 550 (2014).

119. B. Klaus, K. Strimmer, *fdrtool: Estimation of (Local) False Discovery Rates and Higher Criticism* (The Comprehensive R Archive Network, 2021).

120. D. Risso, J. Ngai, T. P. Speed, S. Dudoit, Normalization of RNA-seq data using factor analysis of control genes or samples. *Nat. Biotechnol.* **32**, 896–902 (2014).

121. A. Brionne, A. Juanchich, C. Hennequet-Antier, ViSEAGO: A Bioconductor package for clustering biological functions using Gene Ontology and semantic similarity. *BioData Min.* **12**, 16 (2019).

122. S. Durinck, Y. Moreau, A. Kasprzyk, S. Davis, B. de Moor, A. Brazma, W. Huber, BioMart and Bioconductor: A powerful link between biological databases and microarray data analysis. *Bioinformatics* **21**, 3439–3440 (2005).

123. A. Alexa, J. Rahnenfuhrer, *topGO: Enrichment Analysis for Gene Ontology* (Bioconductor, 2022).

124. L. Garcia-Alonso, C. H. Holland, M. M. Ibrahim, D. Turei, J. Saez-Rodriguez, Benchmark and integration of resources for the estimation of human transcription factor activities. *Genome Res.* **29**, 1363–1375 (2019).

125. R. Kolde, *pheatmap: Pretty Heatmaps* (The Comprehensive R Archive Network, 2019).

126. B. Phipson, C. B. Sim, E. R. Porrello, A. W. Hewitt, J. Powell, A. Oshlack, propeller: Testing for differences in cell type proportions in single cell data. *Bioinformatics* **38**, 4720–4726 (2022).

127. D. W. McKellar, L. D. Walter, L. T. Song, M. Mantri, M. F. Z. Wang, I. de Vlaminck, B. D. Cosgrove, Large-scale integration of single-cell transcriptomic data captures transitional progenitor states in mouse skeletal muscle regeneration. *Commun. Biol.* **4**, 1280 (2021).

128. L. Ting, R. Rad, S. P. Gygi, W. Haas, MS3 eliminates ratio distortion in isobaric multiplexed quantitative proteomics. *Nat. Methods* **8**, 937–940 (2011).

129. S. E. Iismaa, M. Li, S. Kesteven, J. Wu, A. Y. Chan, S. R. Holman, J. W. Calvert, A. U. Haq, A. M. Nicks, N. Naqvi, A. Husain, M. P. Feneley, R. M. Graham. Cardiac hypertrophy limits infarct expansion after myocardial infarction in mice. *Sci. Rep.* **8**, 6114 (2018).

130. A. O. Ward, V. Janbandhu, G. Chapman, S. L. Dunwoodie, R. P. Harvey. An image analysis protocol using cellprofiler for automated quantification of post-ischemic cardiac parameters. *STAR Protoc.* **3**, 101097 (2022).

131. O. Contreras, R. P. Harvey. Single-cell transcriptome dynamics of the autotaxin-lysophosphatidic acid axis during muscle regeneration reveal proliferative effects in mesenchymal fibro-adipogenic progenitors. *Front. Cell Dev. Biol.* **11**, 1017660 (2023).
